# A Human Stem Cell Model of Fabry Disease Implicates LIMP-2 Accumulation in Cardiomyocyte Pathology

**DOI:** 10.1016/j.stemcr.2019.07.004

**Published:** 2019-08-01

**Authors:** Matthew J. Birket, Sophie Raibaud, Miriam Lettieri, Antony D. Adamson, Valerie Letang, Pauline Cervello, Nicolas Redon, Gwenaelle Ret, Sandra Viale, Bing Wang, Bruno Biton, Jean-Claude Guillemot, Vincent Mikol, John P. Leonard, Neil A. Hanley, Cecile Orsini, Jean-Michel Itier

**Affiliations:** 1 Sanofi, Translational Sciences Unit, Sanofi, 13 quai Jules Guesdes, 94400 Vitry-sur-Seine, France; 2 Faculty of Biology, Medicine and Health, Manchester Academic Health Sciences Centre, The University of Manchester, Oxford Road, Manchester M13 9PT, UK; 3 Endocrinology Department, Manchester University NHS Foundation Trust, Grafton Street, Manchester M13 9WU, UK; 4 Sanofi, Translational Sciences Unit, Avenue Pierre Brossolette, 91380 Chilly-Mazarin, France; 5 Sanofi, GBD-Analytical R&D, 211 Second Avenue, Waltham, MA 02451, USA; 6 Sanofi, Rare Disease Science Unit, 153 Second Avenue, Waltham, MA 02451, USA

**Keywords:** Fabry disease, iPSC, cardiomyocyte, heart, lysosome, proteomics, secretome, biomarkers, LIMP-2, maturation

## Abstract

Here, we have used patient-derived induced pluripotent stem cell (iPSC) and gene-editing technology to study the cardiac-related molecular and functional consequences of mutations in *GLA* causing the lysosomal storage disorder Fabry disease (FD), for which heart dysfunction is a major cause of mortality. Our *in vitro* model recapitulated clinical data with FD cardiomyocytes accumulating GL-3 and displaying an increased excitability, with altered electrophysiology and calcium handling. Quantitative proteomics enabled the identification of >5,500 proteins in the cardiomyocyte proteome and secretome, and revealed accumulation of the lysosomal protein LIMP-2 and secretion of cathepsin F and HSPA2/HSP70-2 in FD. Genetic correction reversed these changes. Overexpression of LIMP-2 directly induced the secretion of cathepsin F and HSPA2/HSP70-2, implying causative relationship, and led to massive vacuole accumulation. In summary, our study has revealed potential new cardiac biomarkers for FD, and provides valuable mechanistic insight into the earliest pathological events in FD cardiomyocytes.

## Highlights

• Quantitative proteomics of Fabry disease iPSC cardiomyocytes and secretome
• LIMP-2, a lysosomal protein, accumulates with α-gal A deficiency in cardiomyocytes
• LIMP-2 promotes secretion of cathepsin F and HSPA2/HSP70-2
• Gene correction of Fabry disease iPSCs reverts the cardiomyocyte phenotype

## Graphical Abstract

**Figure:**
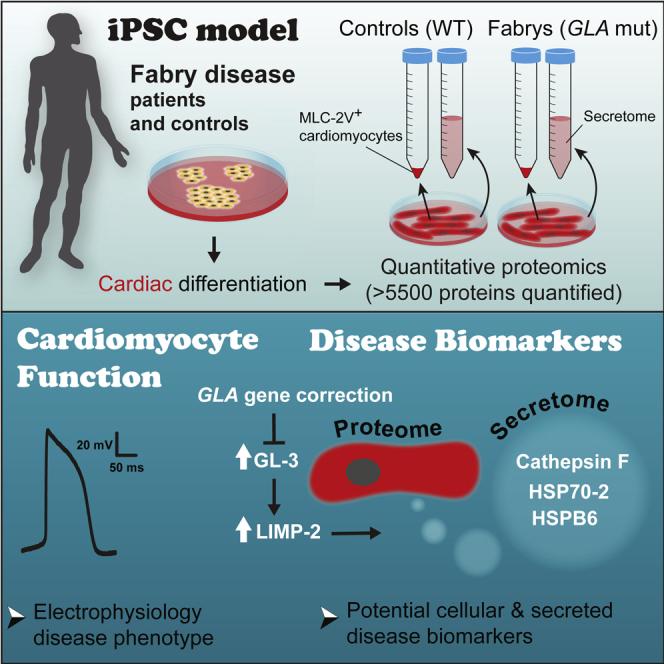

## Teaser

In this article, using an iPSC model, gene editing, and quantitative proteomics, Birket and colleagues gain unique insight into the molecular and functional consequences of heart disease-associated *GLA* mutations in human cardiomyocytes. They identified a panel of cell and secreted biomarkers, the discovery of which may have significant therapeutic relevance for Fabry disease and other lysosomal storage disorders.

## Introduction

Fabry disease (FD) is an inherited lysosomal storage disorder (LSD) caused by mutations in the *GLA* gene on the X chromosome, leading to the deficiency of α-galactosidase A (α-gal A). FD is rare, but LSDs as a group have an incidence of approximately 1:5,000–1:10,000, and may share overlapping pathological mechanisms (Ferraz et al., 2014, Platt, 2014). FD is characterized by the progressive intracellular accumulation of globotriaosylceramide (GL-3) throughout the body, particularly in the vascular tree, nervous system, kidney, and heart (Nagueh, 2014). Most patients develop cardiac involvement, with common manifestations including left ventricular hypertrophy, arrhythmias and conduction disturbances (Linhart and Elliott, 2007). With disease progression, extensive myocardial fibrosis and impaired left ventricular function can develop, and cardiac disease has become the main cause of mortality in FD (Mehta et al., 2009). Enzyme replacement therapy has provided a major therapeutic advance. However, in the heart, clearance of GL-3 from cardiomyocytes (CMs) has not been demonstrated, in contrast to the robust clearance from endothelial cells (Eng et al., 2001, Thurberg et al., 2009).

A major obstacle for advancing therapy for patients with FD is the knowledge gap between the direct molecular consequences of α-gal A deficiency in CMs and the cascade of events driving disease in the heart; the inaccessibility of CMs from patients precludes adequate investigation of these events, especially at early stages. Whether intrinsic myocardial dysfunction drives disease progression and is a primary cause of the electrophysiological and contractile disturbances remains unclear, but is supported by the following evidence: electrocardiogram abnormalities such as a shorter P wave duration, PR interval, and QRS complex indicate an acceleration in both atrial and ventricular conduction in early-stage FD (Namdar et al., 2011), implying that GL-3 accumulation may alter cellular conductive properties; and mechanical defects have been reported in a rare example where patient CMs have been available for *in vitro* investigation (Chimenti et al., 2008).

Recently, our group reported the generation of induced pluripotent stem cells (iPSCs) from patients with FD carrying non-sense mutations in *GLA* (Itier et al., 2014). This model presents the possibility, for the first time, to systematically study the consequences of α-gal A deficiency in CMs at the molecular and functional level and to identify new disease biomarkers. Biomarkers could provide valuable direction for mechanistic investigation and help elucidate the pathological cascades in FD; systemic biomarkers could have further utility for monitoring disease status, response to therapy, and for identifying patients at high risk of cardiac complications.

Using a carefully standardized iPSC cardiac differentiation model, here we provide quantitative data at proteome scale on the cellular phenotype of CMs carrying *GLA* mutations (Fabry CMs). We have identified functional differences in these cells that are consistent with clinical data, and discovered several novel cellular and secreted protein biomarkers. The accumulation of LIMP-2, a lysosomal protein with suggested roles in heart disease, was a robust consequence of α-gal A deficiency, and could drive protein secretion, implying a fundamental role in FD pathology. These new data support the exciting potential of this model for delivering translational benefit for patients with FD.

## Results

### Functional and Structural Characterization of Control and Fabry iPSC-Derived CMs Reveals Increased Excitability in Fabry CMs

iPSCs derived from two patients with FD carrying mutations in *GLA* (Fabry1, c.458G > A; and Fabry2, c.658C > T) that cause classic FD associated with <1% normal α-gal A activity (Altarescu et al., 2001, Maki et al., 2004) were subjected to a cardiac differentiation protocol in monolayer format along with three unrelated wild-type controls.

**Figure 1:**
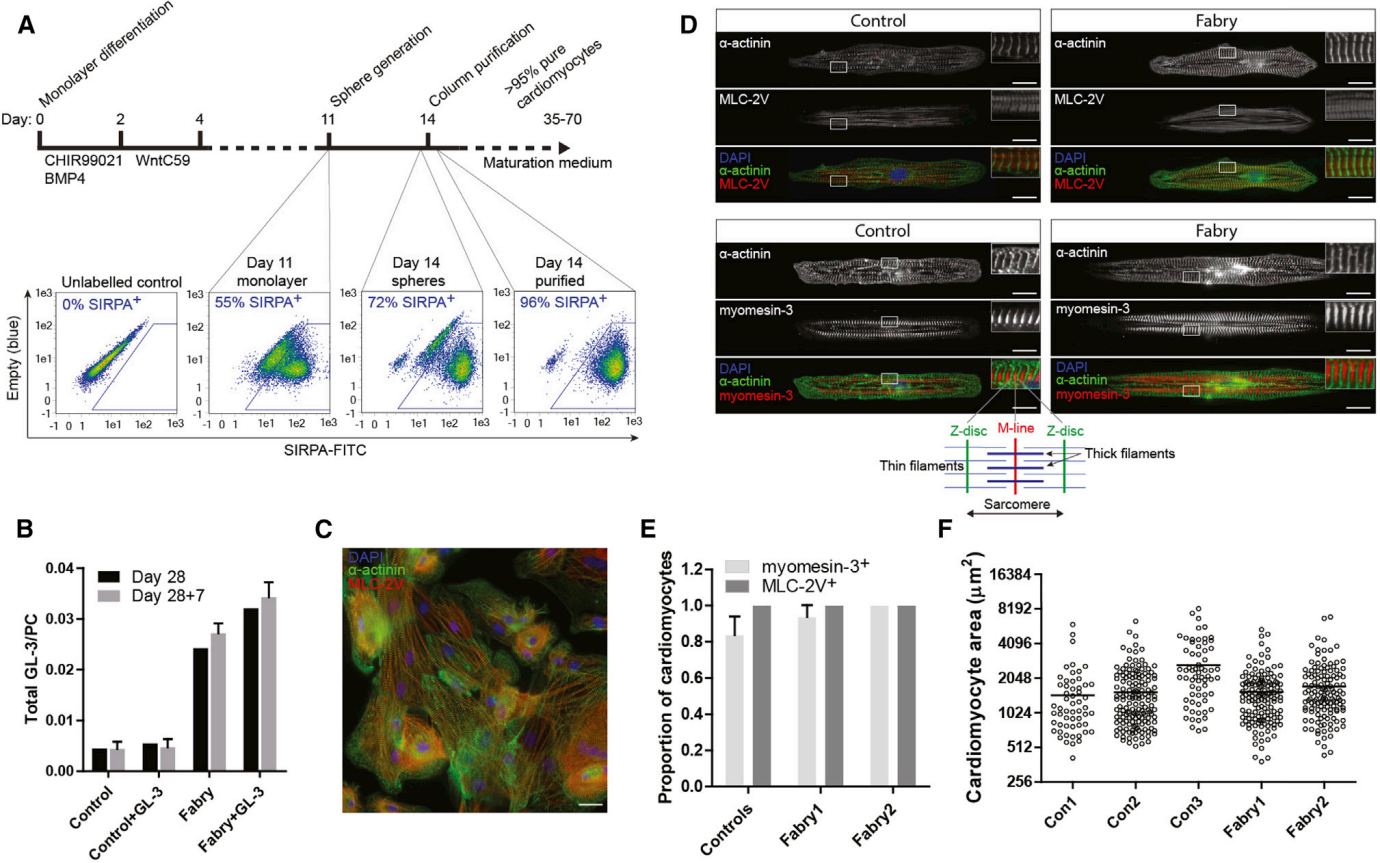
CM Generation from Control and Fabry iPSCs, and Their Structural Characterization (A) Cardiomyocyte differentiation and purification protocol, with FACS plots of SIRPA immunostaining (typical examples) in a differentiated population at day 11, after sphere generation, and again after column purification at day 14. (B) Levels of GL-3, normalized to phosphatidylcholine (PC) content, in control and Fabry CMs at day 28 cultured ±1 μM exogenous GL-3, and in the same populations 7 days later with the exogenous GL-3 removed (day 28 + 7). Bars represent mean ± SD from two independent experiments/differentiations. (C) Typical immunostaining of α-actinin and MLC-2V in a purified population of iPSC-CMs returned to monolayer culture. (D) Typical immunostaining of α-actinin (Z-disc), MLC-2V, and myomesin-3 (M-line) of single control and Fabry1 CMs aligned on micro-patterned glass. The schematic below highlights the sarcomeric structural organization evident from this labeling. (E) The total proportion of single cells expressing myomesin-3 or MLC-2V in an organized pattern. Note that all measured cells expressed MLC-2V. Bars represent mean ± SD from three independent experiments/differentiations (>30 cells quantified per experiment). (F) Single CM 2D area measured using fixed immunolabelled cells. The horizontal line signifies the mean. Data were collected over three independent experiments/differentiations. Scale bar, 20 μm. See also Figure S1.

Robust CM generation (>40% SIRPA^+^) was achieved for all control and disease lines using small molecules targeting the WNT pathway, combined with early BMP4 addition. CMs were enriched using a suspension culture step (Nguyen et al., 2014), and then purified by magnetic column/antibody-bead conjugates and returned to a monolayer; final purity was >95% by SIRPA labeling (Figure 1A; Video S1). α-Gal A deficiency was confirmed in Fabry CMs: by day 28 GL-3 levels were ∼5 times the control level and Fabry CMs were unable to clear exogenous GL-3 as control cells could (Figure 1B). Somewhat surprisingly, all CMs developed strong MLC-2V expression, indicating a universal ventricular-like identity (Figures 1C–1E). As further evidence of authentic CM differentiation, M lines (marked by myomesin-3) were frequently present alternating with Z bands (marked by α-actinin) when imaged on micro-patterned lines (Figure 1D), with this myomesin-3 labeling pattern evident in >80% of control CMs and >90% of Fabry CMs (Figure 1E). CM hypertrophy, a late cardiac phenotype in Fabry patients, was not evident in Fabry CMs at day 45 by 2D single-cell area (Figure 1F) or capacitance (Figure S1).

Video S1. Typical Contracting Spheres Generated Using the Cardiac Differentiation Protocol, and Then a Typical Purified Cardiomyocyte Population after Column Purification

**Figure 2:**
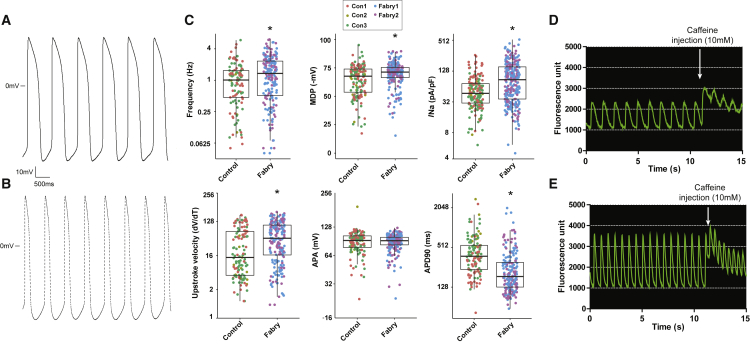
Electrophysiology and Calcium-Handling Measurements in Control and Fabry CMs (A and B) Typical action potential (AP) traces in control (A) and Fabry CMs (B). (C) Quantification of electrophysiological parameters in spontaneously active cells. Points represent individual cells from three control lines and two Fabry lines. Boxes indicate the median ± quartiles and whiskers show the range minus outliers. MDP, maximum diastolic potential; *I*_Na_, peak sodium current density; APA, action potential amplitude; and APD90, action potential duration at 90% repolarization. Statistical significance was calculated using an unpaired t test, ^∗^p < 0.05. (D and E) Typical calcium transients (Cal-520 fluorescence) and response to caffeine injection (where indicated) in control (D) and Fabry CMs (E). Data were collected over four independent experiments/differentiations for electrophysiology and two for calcium imaging. See also Figure S1.

To gain insight into the functional consequences of *GLA* mutations and the propensity for increased conduction and arrhythmias in patients, electrophysiological and calcium-handling measurements were performed on spontaneously active CMs. Results are shown in Figure 2 and Tables S1 and S2. Fabry CMs displayed evidence of increased excitability, with an increased upstroke velocity (dV/dT max) and a slightly higher spontaneous action potential (AP) frequency coupled to a shorter AP duration (Figures 2A–2C). The maximum diastolic potential (MDP) was higher. Importantly, the peak sodium current density (*I*_Na_) was also increased in Fabry CMs; a frequency- and MDP-independent parameter. Consistent with the AP data, calcium transients in Fabry CMs had increased amplitude (ΔF/F), increased rising slope (30%–70%), and decreased peak width duration (corrected for frequency) (Figures 2D and 2E; Table S2). An increased sarcoplasmic reticulum (SR) capacity in Fabry CMs was indicated by the greater caffeine-induced amplitude.

Overall, these data demonstrate that our panel of control and Fabry iPSC lines generate CMs with comparably well-developed structural properties, but Fabry CMs lack α-gal A activity, accumulate GL-3, and develop an increased electrical excitability and a gain of function in calcium handling. This *in vitro* model is therefore a powerful platform for identifying early FD-related biomarkers driven by the primary dysfunction in these cells.

### Identification of Fabry Disease Protein Biomarkers in CMs including LIMP-2

**Figure 3:**
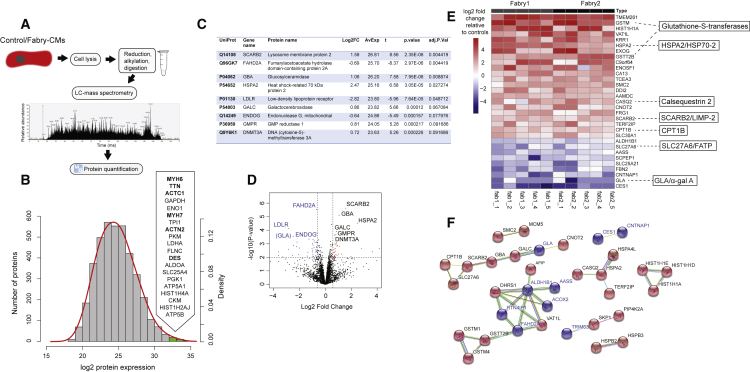
Proteomic Analysis of Control and Fabry CMs (A) Schematic showing the workflow performed for proteome analysis using liquid chromatography-mass spectrometry. (B) Histogram showing the distribution of all detected proteins by their average expression level across all samples (control and Fabry). The red line shows the fitted normal density curve. The top 20 most highly expressed proteins (green bars) are listed in order, with sarcomeric proteins highlighted in bold. (C) “TopTable” output from limma of differentially expressed proteins. Log2FC, log2 fold-change of Fabry samples compared with controls; AvExp, average expression across all samples as log2 value; t, t statistic; adj.P.Val, p value with Benjamini-Hochberg adjustment. (D) Volcano plot showing the results of the limma analysis with significantly upregulated proteins in black and downregulated proteins highlighted in blue. GLA is also highlighted for reference. (E) Heatmap showing protein expression relative to controls in five lysates from each Fabry line, from independent differentiations, for proteins ≥2-fold changed up or down by average in both lines. Some particularly interesting candidates are highlighted to the right. (F) Results of STRING analysis showing potential protein-protein interactions, as run on the 72 differentially expressed proteins in Table S4. Up- and downregulated proteins in Fabry CMs are shown in red and blue, respectively. See also Figure S2.

To perform a global unbiased biomarker discovery screen, purified CMs (day 35) from our panel of iPSCs were used for liquid chromatography-mass spectrometry (Figure 3A). More than 4,400 proteins were detected (≥1 unique peptide each; false discovery rate <1%) in a screen of three control lines in duplicate and the two Fabry lines in triplicate, with individual protein levels measured over ∼5 orders of magnitude (Figure 3B; complete data in Table S3). As confirmation of the accuracy of CM differentiation and cell purity across the tested samples, five of the six most highly abundant proteins (by average across all samples) were cardiac sarcomere components: myosin heavy chain 6 (MYH6), titin (TTN), cardiac actin (ACTC1), myosin MYH7, and α-actinin (ACTN2) (Figure 3B). Moreover, the levels of these and other sarcomeric proteins were not significantly different between control and Fabry samples (Figures S2A and S2B). To assess differential expression, we first performed an analysis with limma (Ritchie et al., 2015) using a control versus Fabry model. At a cutoff of ≥0.5 log2 fold-change and adjusted p value <0.1 (Benjamini-Hochberg) we identified nine differentially expressed proteins (Figure 3C). Compared with controls, lysosomal-specific proteins including lysosomal membrane protein 2 (SCARB2/LIMP-2), glucocerebrosidase (GBA), and galactocerebrosidase (GALC) were upregulated in Fabry CMs; also upregulated were heat shock-related 70 kDa protein 2 (HSPA2/HSP70-2), GMP reductase 1 (GMPR), and DNA-methyltransferase 3A (DNMT3A). Fumarylacetoacetate hydrolase domain-containing protein 2A (FADH2A), low-density lipoprotein receptor, and mitochondrial endonuclease G (ENDOG) were among the downregulated proteins. ENDOG was previously shown to regulate mitochondrial function, lipid metabolism, and hypertrophy in CMs (McDermott-Roe et al., 2011). Loading with the cationic fluorescent dye TMRM indicated that Fabry CMs may indeed have a lower mitochondrial membrane potential than controls (Figure S2C), which could indicate a mitochondrial functional deficiency, although mtDNA-encoded proteins were not significantly decreased at day 35 (Figure S2D). The enrichment of lysosomal-associated proteins in this list is significant (Fisher's exact test, p < 0.01) when considering that only 179 of these 4,464 proteins have been associated with lysosomes in the literature (Brozzi et al., 2013). Interestingly, LIMP-2 is the lysosomal trafficking receptor for GBA, but published data suggest that it may have additional roles in the heart and in lysosomal biogenesis, making it a particularly interesting functional candidate (Reczek et al., 2007, Schroen et al., 2007).

To increase the power of this approach, and overcome limitations in parallel sample processing by mass spectrometry, we examined internal ratio differences in protein levels against the control mean so that the number of replicates could be increased. Data from a smaller-scale mass spectrometry run were added (Table S3), giving five independent replicates per group in total. A total of 4,915 proteins were detected in the second run, giving a combined 5,178 proteins in total, 4,201 proteins being common to both runs. This list of 4,201 proteins was filtered for proteins with ≥0.5 log2 fold-change for both Fabry lines individually against controls and an adjusted p value <0.05 (one-sample t test; μ = 0), which identified 53 up- and 19 downregulated proteins (Table S4). Seven of the nine previously identified candidates also qualified by these criteria (SCARB2/LIMP-2, GBA, GALC, HSPA2/HSP70-2, DNMT3A, FADH2A, and ENDOG), further enhancing their validity as Fabry biomarkers. Figure 3D shows the proteins with ≥2 fold-change in a heatmap. A decrease in GLA/α-gal A was revealed in this list, peptides for which were almost completely absent in Fabry CMs as expected. Genome ontology showed that the “glycosylceramide catabolic process” was the most significantly enriched biological process, and that “lysosomal lumen” the most significant cellular component from this list of 72 proteins (Tables S5 and S6). However, many other non-lysosomal proteins also emerged, including three glutathione S-transferases and three additional heat shock proteins (HSPs), which were all increased in Fabry CMs (GSTM1, 7.9-fold; GSTM4, 1.5-fold; GSTT2B, 5.1-fold; HSPB2, 1.4-fold; HSPB3, 1.7-fold; and HSPA4L, 2.1-fold). The SR calcium-buffering protein, calsequestrin 2 (CASQ2), and the mitochondrial fatty acid transporter, carnitine O-palmitoyltransferase 1 (CPT1B), were also increased by 3.0- and 2.4-fold, respectively. Protein-protein interaction (PPI) network analysis using STRING identified 34 edges (putative PPIs) between these 72 differentially expressed proteins (PPI enrichment p value <0.01) (Figure 3F).

**Figure 4:**
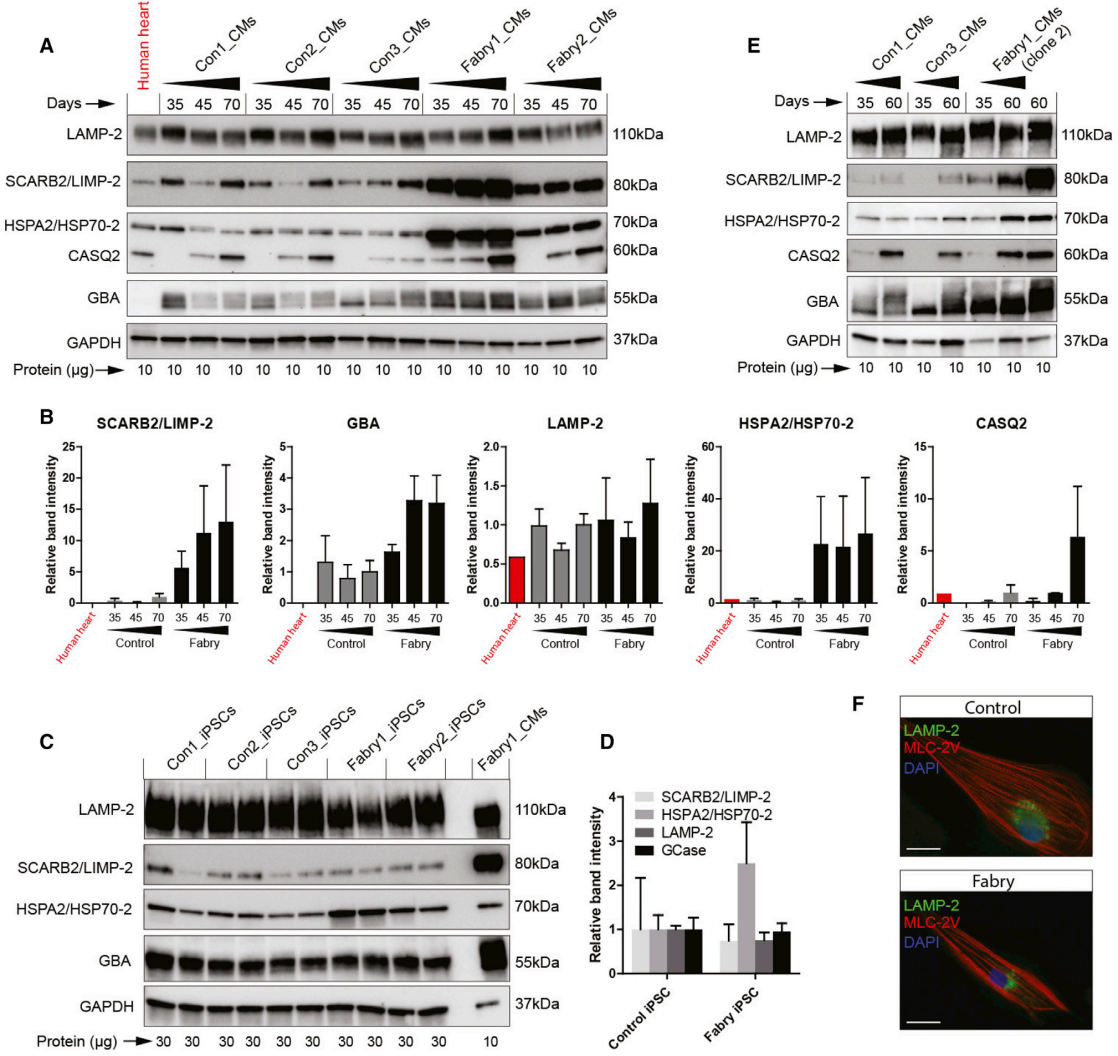
Validation of Candidate Fabry Protein Biomarkers by Western Blot (A) Western blots of control and Fabry CMs taken at three time points from three differentiations. Protein from a human heart lysate was loaded in the first lane. (B) Quantification of western blots in (A) grouped as control and Fabry, calculated as relative band density normalized to GAPDH levels. (C) Western blots of undifferentiated control and Fabry iPSCs with independent duplicates. A Fabry CM population is shown in the far right lane as reference. (D) Quantification of the iPSC western blots in (C), calculated as relative band density normalized to GAPDH levels. (E) Western blots showing the levels of the candidate Fabry biomarkers in CMs derived from a second clone of Fabry1 iPSCs at two time points of differentiation. (F) Immunocytochemistry showing typical LAMP-2 and MLC-2V staining in control and Fabry CMs. Bar data indicate the mean ± SD. Scale bar, 20 μm. See also Figure S3.

To validate a subset of these potential Fabry biomarkers with interesting roles in lysosomes, heat shock response, and calcium handling, western blots were performed using independent cell lysates from CMs at 35–70 days of differentiation, along with normal human heart tissue sample for reference. The later time points were included to assess the impact of continued cell maturation and pathogenesis. An upregulation of SCARB2/LIMP-2, GBA, and HSPA2/HSP70-2 was observed in both Fabry lines beyond 35 days of differentiation (Figures 4A and 4B). LIMP-2 and GBA were not increased in Fabry iPSCs before differentiation (Figures 4C and 4D), whereas HSP70-2 already showed increased abundance, most notably in Fabry1 iPSCs. However, another iPSC clone from this patient with lower baseline HSP70-2 levels (Figure S3A) still showed an increase relative to controls after differentiation (Figures 4E and S3B). Elevated levels of LIMP-2 and GBA in Fabry CMs were not explained by increased lysosomal biogenesis, as LAMP-2 was not increased and lysosomes were not more numerous by immunocytochemistry (Figures 4A, 4B, and 4F). CASQ2 expression was clearly maturation dependent, but when detectable at later time points was increased in Fabry CMs, consistent with the enhanced calcium handling of Fabry CMs shown above. Overall, our western blot data strongly support the quantitative accuracy of the proteomics data.

### Secretome Analysis Identifies Secreted Fabry Biomarkers including Cathepsin F

**Figure 5:**
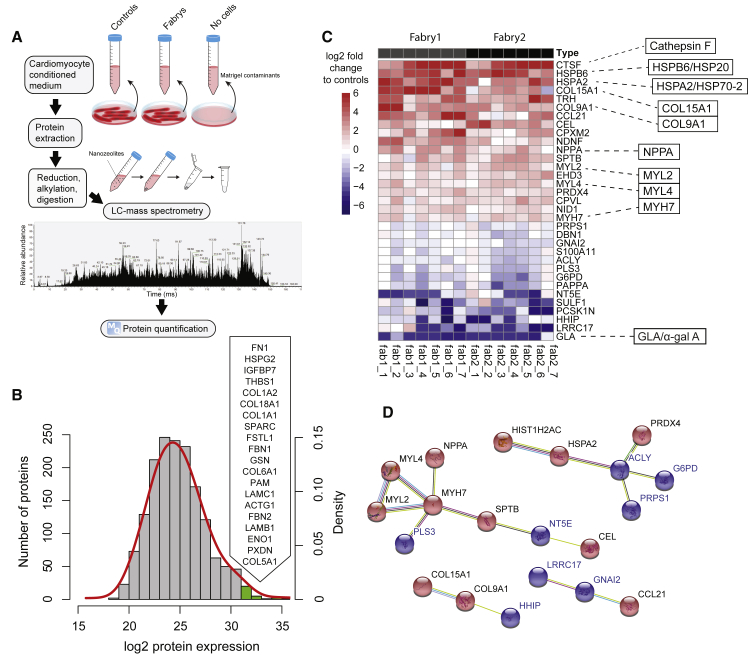
Secretome Analysis of Control and Fabry CMs (A) Schematic showing the workflow for protein isolation and secretome analysis using liquid chromatography-mass spectrometry from CM-conditioned medium. (B) Histogram showing the distribution of all detected proteins by their average expression level across all samples (control and Fabry). The red line shows the fitted normal density curve. The top 20 most highly expressed proteins (green bars) are listed in order. (C) Heatmap showing protein expression relative to controls in seven lysates from each Fabry line, from independent differentiations, of proteins >1 log2 fold changed up or down in both lines by average. Some particularly interesting candidates are highlighted to the right. (D) Results of STRING analysis showing potential protein-protein interactions, as run on the 34 differentially expressed proteins in Table S7. Up- and downregulated proteins in Fabry CMs are shown in red and blue, respectively. See also Figure S4.

To identify biomarkers differentially secreted by Fabry CMs, mass spectrometry was performed on proteins isolated from CM-conditioned medium (Figure 5A). A total of seven samples from each Fabry line were compared with samples from four control lines across three mass spectrometry runs; each sample from an independent differentiation and experiment. After subtraction of potential matrix-derived (Matrigel) contaminants, a total of 3,013 proteins were identified (≥1 unique peptide each; false discovery rate <1%), which spanned ∼5 orders of magnitude in abundance like the cell proteome (Figure 5B; complete data in Table S3). A list of the top 20 most highly abundant proteins across all control and Fabry secretomes is shown in Figure 5B, and was made up largely of extracellular matrix and associated proteins. Proteins were searched by databases for known plasma proteins (Nanjappa et al., 2014), and proteins with a signal peptide destined toward the secretory pathway (Petersen et al., 2011), using the Human Protein Atlas (Uhlén et al., 2015). The total secretome was enriched for plasma proteins (50% versus 35% in the proteome) and proteins with signal peptides (24% versus 9% in the proteome). A total of 718 proteins were uniquely identified in the secretome, 58% of which had a signal peptide. Enrichment was also seen for known cardiac secreted proteins such as atrial natriuretic peptide (NPPA) and natriuretic peptide B, which were both at levels above the 90th percentile in the secretome but barely detectable in the cell proteome. Together, these observations provide high confidence that this is a bona fide CM secretome.

To identify differentially abundant proteins between control and Fabry secretomes, the same multi-experiment filtering approach was performed as for the proteome: ≥0.5 log2 fold-change for both Fabry lines individually against controls and an adjusted p value <0.05 (one-sample t test; μ = 0). This identified 20 up- and 14 downregulated proteins (Table S7). Figure 5C shows the proteins with ≥2 fold-change in a heatmap. Importantly, the most downregulated protein in Fabry CM secretomes was GLA/α-gal A, which interestingly was more easily detectable in control secretomes than in the cell proteomes. The most highly overrepresented protein in Fabry CM secretomes was the lysosomal cysteine protease cathepsin F (CTSF), which was increased by 30-fold. Also highly overrepresented was the heart-enriched and cardioprotective HSPs, HSPB6/HSP20 (22-fold) (Fan and Kranias, 2011) and HSP70-2 (18-fold). HSP70-2 is therefore not only elevated in Fabry CMs but also secreted at higher levels. Other notable proteins that were increased included the known heart disease biomarker NPPA (2.7-fold); three cardiac myosins (MYL2, MYL4, and MYH7); and two collagen molecules (COL15A1 and COL9A1). PPI network analysis of the differentially expressed secretome proteins identified several putative interactions (Figure 5D), and combining this with the cell proteome list generated a large network with 82 edges (PPI enrichment p < 0.01), with ATP citrate synthase (ACLY) forming a putative hub node (Figure S4). ACLY is responsible for production of extramitochondrial acetyl-coA and has important roles in nutrient catabolism and lipid biosynthesis (Pinkosky et al., 2017).

### *GLA* Mutation Correction and Gene Knockout Support the Validity of Fabry Biomakers

**Figure 6:**
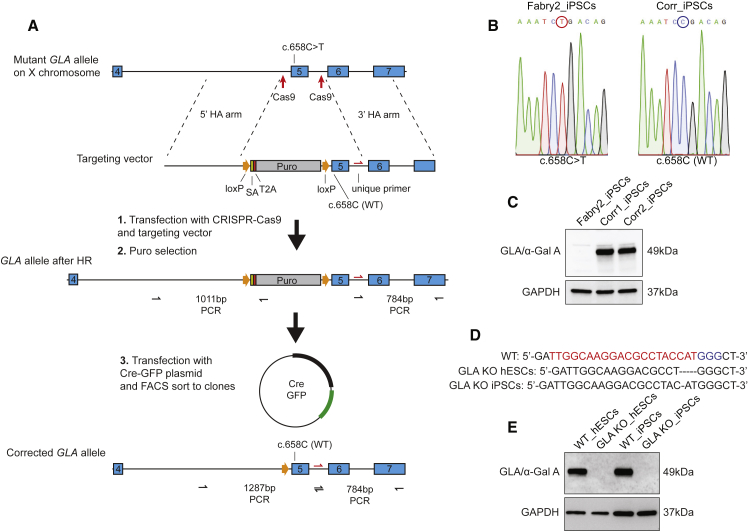
Genetic Correction of *GLA* Point Mutation and Generation of *GLA* Knockouts (A) Schematic showing the strategy used to correct the *GLA* c.658C > T mutation and the three main steps: CRISPR-mediated knockin, selection, and removal of selection. Successful homologous recombination and Cre-mediated excision of the selection cassette could be confirmed using the PCR amplicons shown. (B) Sequence chromatograms across nucleotide c.658 showing successful correction of the mutation. (C) Western blot showing restoration of α-gal A expression in two sequence-corrected clones. (D) In-del mutations in *GLA* introduced into wild-type hESCs and iPSCs for gene knockout. The red and blue nucleotides signify the crRNA and PAM sequences, respectively. (E) Western blot showing complete knockout of α-gal A expression as a result of the mutations in (D).

To assess the impact of α-gal A deficiency in CMs more directly, we took two strategies. Firstly, to correct the *GLA* point mutation in one of the Fabry iPSC lines, and secondly to generate in-del mutations in *GLA* in wild-type hESC/iPSC lines to create gene knockouts. To correct the c.658C > T mutation in Fabry2 iPSCs we used a selection strategy (Figure 6A). Because *GLA* is expressed in hPSCs we incorporated a splice acceptor and puromycin resistance gene, together with wild-type exon 5, into the *GLA* gene, so that successfully targeted cells could be selected. After Cre-mediated excision of the selection cassette and cell cloning, full-length wild-type *GLA* expression was restored in all analyzed clones (Figure 6B). Two were expanded and the restoration of α-gal A protein expression was confirmed (Figure 6C). *GLA*/α-gal A knockout clones were successfully derived from MAN13 hESCs (c.121_125delTACCA) and con3 iPSCs (c.123delC) (Figures 6D and 6E).

**Figure 7:**
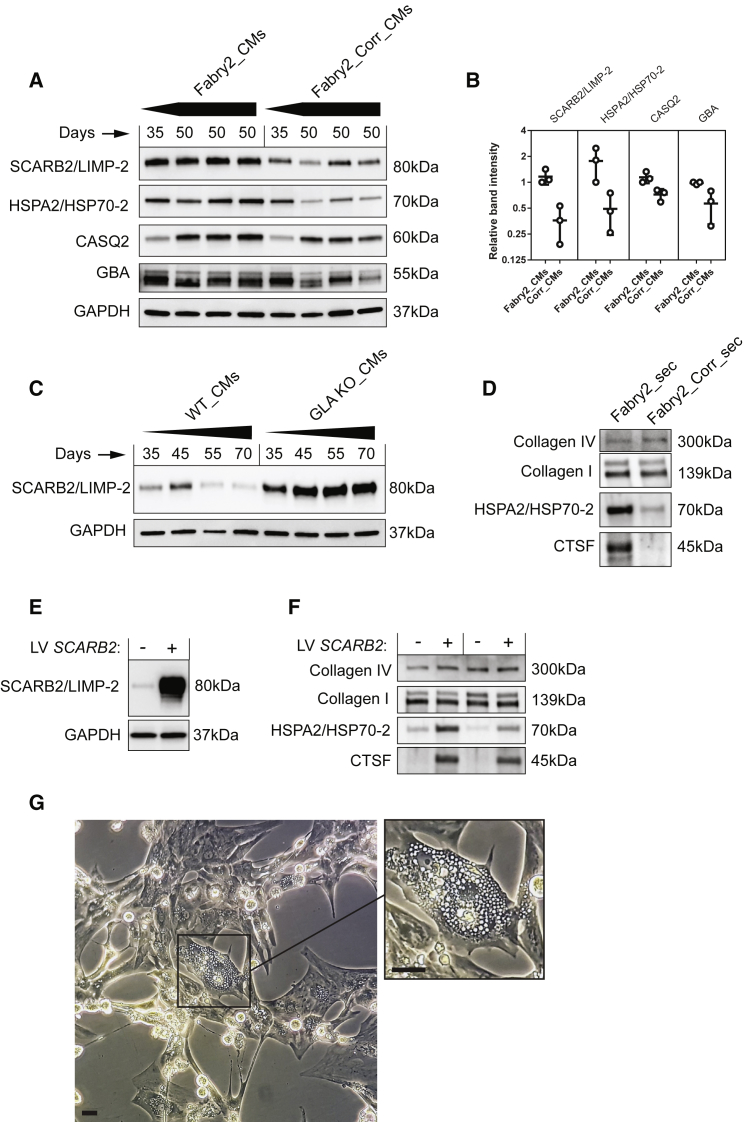
Validation of Biomarkers Using Isogenic Controls and Impact of LIMP-2 Accumulation (A) Western blots of Fabry and gene-corrected CMs taken at two points from four independent differentiations. (B) Quantification of western blots in (A). Error bars show SD. (C) Western blot of wild-type CMs derived from hESCs and *GLA* knockouts taken at different points from four independent differentiations. (D) Western blot of secretome samples from Fabry- and corrected CM-conditioned medium. (E) Western blot showing overexpression of LIMP-2 in wild-type CMs by lentivirus. (F) Western blot of secretome samples from two independent experiments showing the impact of LIMP-2 overexpression on protein secretion. (G) Image of purified CMs overexpressing LIMP-2 by lentivirus. The high magnification image highlights the ubiquitous vacuoles in one particular cell. Scale bar, 100 μm. See also Figure S5.

Following cardiac differentiation of these isogenic lines, the four protein biomarkers previously validated by western blot were assessed over time. Correction of the *GLA* c.658C > T mutation diminished the levels of all these markers when quantified at day 50 (Figures 7A and 7B). In hESC-CMs, *GLA*/α-gal A knockout cells showed a similar marker profile to the Fabry CMs (Figures S5A and S5B), with LIMP-2 accumulation being the most dramatic change (Figure 7C). LIMP-2 accumulation was further confirmed in iPSC-CMs with *GLA* knockout (Figure S5C), suggesting that this is a robust response to α-gal A deficiency in CMs. *SCARB2* mRNA levels were unchanged, demonstrating that accumulation is not due to increased gene expression (Figure S5D). Secretion of CTSF and HSP70-2 were assessed by western blotting and were both found to be markedly decreased after gene correction (Figure 7D).

### LIMP-2 Accumulation Drives the Secretion of Cathepsin F and HSP70-2

As LIMP-2 is known to have an important role in endolysosome biogenesis, we investigated whether LIMP-2 accumulation alone could drive the secretion of these proteins. Measurements after LIMP-2 overexpression in wild-type CMs or 293FT cells confirmed this hypothesis (Figures 7E, 7F, and S5). Collagen I and IV levels were unaffected and no increased cell death was observed. Light microscopy revealed an increase in vacuole formation in LIMP-2 overexpressing CMs, a feature of late-stage Fabry cells (Figure 7G and Video S2) (Chimenti et al., 2008), although these were not observed in the unmanipulated Fabry CMs within the studied time frame.

Video S2. Purified Cardiomyocytes Overexpressing LIMP-2 as in Figure 7

## Discussion

Many disease phenotypes are cell-type specific and must be investigated in the appropriate cellular context. Here, we used patient-derived iPSCs to study the consequences of *GLA* mutations in CMs as a model of FD. Although Fabry CMs were comparable with controls in their size and structural maturity, developing uniform MLC-2V expression and sarcomere M lines, they accumulated GL-3 and showed evidence of increased electrophysiological excitability. Moreover, a proteomics-based screen identified FD-specific changes in specific proteins involved in lysosomal function and heat shock response, presenting potential new disease biomarkers. The accumulation of LIMP-2 was the most significant difference and was found to directly promote the secretion of the lysosomal protease CTSF and HSP70-2, suggesting that α-gal A deficiency causes major changes to endolysosomal pathways. Our data support the concept that this hPSC model can reveal important early molecular changes in FD.

Heart disease is the main cause of death in patients with FD, with the accumulation of lysosomal GL-3 in the heart frequently resulting in the progressive development of hypertrophy and arrhythmia. Increased conduction velocity in atrial and ventricular CMs has been proposed as one possible cause of arrhythmic risk in FD (Namdar, 2016). Ion handling in endothelial cells and neurons has been reported to be affected by GL-3 accumulation (Choi et al., 2014, Choi et al., 2015, Park et al., 2011); however, data on Fabry CMs were lacking. Peak sodium current density is an important determinant of myocardial conduction velocity (King et al., 2013). Here, we show that Fabry CMs display an enhanced *I*_Na_ relative to controls, and an increased dV/dT max, suggesting enhanced sodium channel function, as well as displaying a higher spontaneous AP frequency and a shorter AP duration. Enhanced function was equally observed at the level of calcium handling. Imbalanced glycosphingolipid levels may therefore lead to alterations in ion channel expression, or more likely to changes in their trafficking or turnover, resulting in direct effects on CM electrophysiology, with potential implications for arrhythmia.

Human PSC technology makes it possible to study heart diseases in the appropriate cells at a genome- and proteome-wide level. However, in contrast to transcriptomic data on PSC CMs, which are now ubiquitous, there has been limited published proteome-level data on purified populations of these cells. The composition of the CM secretome is even less well defined, even though cardiac-derived secreted proteins, or cardiomyokines, may have important roles in cell-cell communication in heart disease (Doroudgar and Glembotski, 2011), and are of special interest concerning the potential paracrine therapeutic effects of human PSC CMs after transplantation into the diseased heart (Tachibana et al., 2017). In this study, using well-characterized and carefully purified iPSC-CMs, we report the quantification by mass spectrometry of >5,000 cellular proteins, and a further ∼700 proteins uniquely identified in their secretomes. These datasets will be valuable resources for the research community and benchmarks for future studies.

Several of the proteins that we found to be differentially expressed/abundant in Fabry CMs or in the extracellular space have known roles in other LSDs (including Gaucher disease, SCARB2/LIMP-2 and GBA; Krabbe disease, GALC; and ceroid lipofuscinosis, CTSF), but had never been implicated in FD. Our data also implicate HSPs in the cellular response to α-gal A deficiency in CMs, specifically family members HSPB2, HSPB3, HSPA4L, HSPB6/HSP20, and HPS70-2, which may have cardioprotective roles. HSPs are a group of chaperones important for maintaining normal cell metabolism and function under stress. HSP70-2, a member of the highly conserved HSP70 family (Daugaard et al., 2007), was increased in Fabry CMs, and was also secreted at higher levels. Interestingly, HSP70 treatment has been shown to reverse lysosomal pathology in models of LSDs including the murine model of FD (*Gla*^−/–^) (Kirkegaard et al., 2016). Whereas HSP70-2 has been suggested to lack the lysosome stabilization activity of HSP70, recombinant HSP70-2 was also reported to be similarly taken up into the lysosomal lumen (Kirkegaard et al., 2010). Also increased in Fabry secretomes was HSPB6/HSP20, which is a cardioprotective and secreted HSP known to be upregulated in CMs by stress (Chu et al., 2004, Martin et al., 2014, Zhang et al., 2012). Overall, our data support a possible role for these HSPs in an adaptive response to α-gal A deficiency and as potential new biomarkers in FD.

In addition to specific HSPs, proteomics analysis of the Fabry CM secretome identified CTSF as being highly enriched. The secretion of cathepsins has been documented previously, and these proteins may have important extracellular roles (Fonovic and Turk, 2014, Öörni et al., 2004). Enhanced secretion may be due to impaired lysosomal targeting, lysosomal leakage, or increased active secretion in Fabry CMs. Of relevance, certain cathepsins have been shown to be upregulated in plasma of patients with the LSD Gaucher disease (Moran et al., 2000). Although mechanisms for the active secretion of CTSF, HSPB6, and HSP70-2 have not yet been specifically defined, extensive work has been published describing the active secretion of HSP70 in response to heat shock, which may be of important relevance (Mambula and Calderwood, 2006). Specifically, HSP70 release has been shown to involve transit through an endolysosomal compartment containing cathepsin D, and can be inhibited by lysosomotropic agents, similar to interleukin-1β (Andrei et al., 1999). Enhanced exocytosis of HSP- and CTSF-containing vesicles in Fabry CMs is therefore an interesting possibility.

LIMP-2 was the most significant biomarker in our proteome analysis of Fabry CMs, and was found to accumulate through a post-transcriptional mechanism. LIMP-2 is the lysosomal trafficking receptor for GBA; however, LIMP-2 has also been shown at intercalated disc structures in the heart, and its expression in CMs found to correlate with mechanical stress and to play a crucial role in CM hypertrophic response (Schroen et al., 2007). Of further relevance to FD, LIMP-2 has been shown to be a powerful regulator of endosome and lysosome biogenesis and capable to inducing the formation of intracellular vacuoles (Kuronita et al., 2002). Similar structures are widely reported in Fabry cells *in situ*, although typically enclosing concentric lamellar structures (Pieroni et al., 2003). Although the Fabry CMs in this study did not clearly developed such vacuoles, other publications have noted these *in vitro* (Itier et al., 2014, Kuramoto et al., 2018). Nevertheless, overexpression of LIMP-2 clearly induced this phenotype.

With an apparent role in endolysosomal organization, we tested whether LIMP-2 accumulation might be a primary mediator of HSP70-2 and CTSF secretion. Our data support this idea, as secretion of both proteins could be enhanced by overexpressing LIMP-2 in wild-type CMs. Whether this has an adaptive role in FD in potentially aiding the clearance of GL-3, or has pathological consequences, will be important to investigate. In CMs, any changes in trafficking or vesicle secretion/exocytosis could have significant consequences for ion channel density at the plasma membrane, and therefore CM electrophysiology. Recent data suggesting that exosome production might be increased in *GLA* knockout CMs also merit further study in this context (Song et al., 2019).

In the observed experimental time frame of the current study, CM hypertrophy was not observed. This is contrary to what has been reported by some (Chien et al., 2016, Chou et al., 2017), but not all studies investigating FD iPSC-derived CMs (Kuramoto et al., 2018). By contrast, the accumulation of GL-3 has been universally reported. It will therefore be important to investigate by what mechanism this primary dysfunction can lead to hypertrophy, and whether LIMP-2, which has shown to be essential for adaptive hypertrophy in a rat model of hypertension, plays a role in this (Schroen et al., 2007). The isogenic lines and genetic tools presented here will facilitate these future investigations.

In summary, our study has shown the power of the iPSC model to reveal early functional changes and the development of a distinctive biomarker expression profile in FD CMs. These biomarkers may be of utility in drug screening and in elucidating the earliest pathological events and cascades in FD cells. Quantification in patient plasma and urine samples will be an important next step toward validating their relevance in patients. Heterogeneity is a major feature of FD, and personalized risk stratification for arrhythmias in patients, for example, through biomarker profiling, might help cardiologists judge the need for cardioverter defibrillator implantation. Inclusion of patients with FD in large-scale sequencing efforts, such as the UK's 100,000 Genomes Project (Genomics England, 2017), will provide further opportunity for stratification by enabling the study of genetic variation in the putative genes and pathways implicated in the heart, correlated to clinical status. The wealth of new data reported here may have implications beyond FD, given that some of the principal candidates, including LIMP-2, are suggested to be involved in other heart diseases and may therefore represent common players acting in CM pathology. A better understanding of these mechanisms will no doubt accelerate the development of more effective and increasingly personalized therapies for patients.

## Experimental Procedures

See Supplemental Information for full experimental procedures.

### hPSC Culture and Cardiac Differentiation

The derivation of iPSC lines from two male patients with FD carrying non-sense mutations in *GLA* was described previously (Itier et al., 2014). The patient fibroblasts were obtained from the Coriell Institute for Medical Research. In this article, clone GM00107 (c.485G > A; W162X) is referred to as Fabry1 and clone GM00881 (c.658C > T; R220X) as Fabry2. Four healthy male control iPSC lines were also derived internally from skin fibroblasts using the same protocol. MAN13 hESCs were kindly provided by the Kimber lab (Ye et al., 2017). MAN13 hESCs are a UK Stem Cell Bank registered line and this study was performed in full compliance with the laws, regulations, and procedures that govern hESC use in the UK. The study conducted is in full compliance with Sanofi guidelines on the use of hESCs. All experiments involving hESCs were performed at The University of Manchester. hPSCs were maintained in mTeSR medium (Stem Cell Technologies). Cardiac differentiation was induced as follows: 10 ng/mL BMP4 + 4 μM CHIR991021 (Miltenyi Biotec) for 48 h and then 2 μM Wnt C-59 (Selleckchem) for 48 h. At day 11, cells were transferred to suspension for 3 days. For suspension culture and plating, cells were maintained in CM medium. See Supplemental Information. CMs were purified using a MACS column-based PSC-Derived Cardiomyocyte Isolation Kit (Miltenyi, no. 130-110-188) and replated on Matrigel-coated plastic. After 2 days the medium was changed to a maturation-promoting formula containing 5 nM T3 + 5 nM dexamethasone + 20 μM palmitic acid (conjugated to fatty acid-free BSA) + 0.25% FBS, as supported by our previous work (Birket et al., 2015). CMs were analyzed between days 35 and 70 of differentiation.

### CRISPR-Cas9 Gene Editing

The *GLA* c.658C > T mutation was corrected by delivering ribonucleoprotein (RNP) complexes of CRISPR-Cas9 targeting sequences flanking exon 5, along with a targeting vector into Fabry2 iPSCs by nucleofection. RNP complexes were made by complexing cr:tracRNA duplex (IDT) with S.p. Cas9 Nuclease V3 (IDT). The targeting vector incorporated a 5′ homology arm of 801 bp and a 3′ homology arm of 750 bp flanking corrected exon 5 (pUC57-5′H-loxP-SA-2A-Puro-loxP-correctExon-3′H). After 48 h of electroporation, iPSCs were selected with puromycin for 48 h, then electroporated with a Cre expression vector (pCAG-Cre-IRES2-GFP; Addgene plasmid no. 26646), FACS sorted for GFP expression, and directly cloned. Expanded clones were screened by PCR, taking advantage of a unique intronic primer site introduced 3′ of exon 5, and sequenced. All analyzed clones were correctly targeted.

To generate *GLA* knockout hPSC lines, MAN13 hESCs and con 3 iPSCs were electroporated as above using a crRNA sequence targeting exon 1 (5′ TTGGCAAGGACGCCTACCAT 3′), which was found to induce in-del mutations with 60%–80% efficiency. Cells were cloned and screened by sequencing. Clones with frameshift mutations and undetectable α-gal A expression by western blot were used for subsequent experiments: MAN13 hESCs (*GLA* c.121_125delTACCA) and con3_iPSCs (*GLA* c. 123delC).

### Immunocytochemistry and Cell Size Determination

Dissociated iPSC-CMs differentiated for ∼40 days were seeded at single-cell density on glass coverslips micro-patterned with 20-μm-wide gelatin lines as described previously (Birket et al., 2015). Four days later cells were fixed, permeablized, and labeled. Antibodies were as follows: anti-α-actinin (Sigma; A7811), anti-MLC-2V (Proteintech; 10906-1-AP), and anti-myomesin-3 (Proteintech; 17692-1-AP). Individual separated CMs were identified based on α-actinin labeling and then MLC-2V or myomesin-3 labeling was also imaged and recorded. Cell area was calculated using a thresholding mask based on α-actinin labeling.

### Electrophysiology and Calcium Imaging

Dissociated iPSC-CMs differentiated for 30 days were seeded at single-cell density on fibronectin-coated glass coverslips and used for measurement 3–8 days later. Patch-clamp experiments were performed at 37°C and cells were continuously perfused with Tyrode's buffer. Only spontaneously active single cells were measured. Calcium imaging was performed on monolayers of iPSC-CMs using the Cal-520 assay as outlined in the product information sheet provided with the Hamamatsu FDSS *μCell* (Hamamatsu Photonics, Hamamatsu, Japan). Assay buffer was made from Hank's balanced saline solution buffered with 20 mM HEPES. The caffeine response was assessed by injecting 10 μL of caffeine at 50 mM resulting in a final concentration of 10 mM. Fluorescence measurements are represented as relative fluorescence unit. For each experiment, spontaneous calcium transients were analyzed over 2 min. Frequency, basal fluorescence (F), amplitude (ΔF/F), peak width at 90% of recovery (PWD-90%), corrected peak width (corrected PWD-90%), calculated by Fridericia's formula and rising slope (30%–70%) were measured (exemplified in Figure S1).

### Proteomics

See Supplemental Information.

### Western Blot

Ten or 30 μg of cell lysate, or secretome protein isolate, was loaded onto a 4%–20% SDS-PAGE gel (Bio-Rad, no. 5678094) and transferred to a PVDF membrane using a Trans-Blot Turbo Transfer Pack (Bio-Rad, no. 170–4157). Membranes were blocked in 5% milk in TBS-T and incubated in primary antibody overnight at 4°C. Antibodies and their dilutions were as follows: Anti-LIMP-2 (Abcam, ab176317; 1:1,000), anti-HSP70-2 (Abcam, ab108416; 1:1,000), anti-calsequestrin 2 (Proteintech, 18422-1-AP; 1:1,000), anti-GCase (internal; 1:500), anti-LAMP-2 (Santa-Cruz, Sc-18822; 1:500), anti-GLA (Abcam, ab168341; 1:1,000), anti-CTSF (Abcam, ab200650; 1:500), anti-collagen I (SouthernBiotech, 1310-01; 1:1,000), anti-collagen IV (Novocastra, NCL-COLL-IV; 1:1,000), anti-GAPDH (Abcam; 1:3,000). Horseradish peroxidase (HRP)-conjugated secondary antibodies were as follows: anti-mouse IgG(H + L) HRP (Thermo Fisher Scientific, no. 32230; 1:10,000) and anti-rabbit IgG(H + L) HRP (Thermo Fisher Scientific, no. 32460; 1:10,000).

### Lentiviral Transduction

*SCARB2* was overexpressed by lentivirus produced using a pLenti CMV/TO Puro DEST vector (Addgene plasmid no. 17452). The empty vector, or vector with a GFP insert, were used as controls. Lentiviral particles were concentrated using ultracentrifugation and used to transduce CMs at the time of their purification on day 14. Transduced cells were selected with 0.75 μg/mL puromycin and then cultured as normal. Conditioned medium was collected 5–8 days after transduction of CMs or 293FT cells.

## Author Contributions

Conceptualization, M.J.B., J.M.I., and C.O.; Investigation, M.J.B., S.R., A.D.A., V.L., P.C., N.R., and B.B.; Writing – Original Draft, M.J.B.; Writing – Review & Editing, M.J.B., N.A.H., C.O., and J.M.I.; Resources, M.L., G.R., S.V., B.W., and N.A.H.; Supervision, B.B., J.C.G., V.M., J.P.L., N.A.H., C.O., and J.M.I.

## Supplemental Information

Document S1. Supplemental Experimental Procedures, Figures S1–S5, and Tables S1, S2, and S4–S7

Table S3. Complete Cardiomyocyte Proteome and Secretome Data in Excel Format Tab 1 (“Exp1_proteome”) shows data from the first cell proteomics experiment and tab 2 (“Exp2_proteome”) shows data from the second experiment. Tab 3 (“Exp1_secretome”) shows data from the first secretome experiment, tab 4 (“Exp2_secretome”) shows data from the second secretome experiment, and tab 5 (“Exp3_secretome”) shows data from the third secretome experiment. Protein levels are shown as log2 values. AvExp, the mean expression across all samples in the run. For sample names: con, control lines, and fab, Fabry lines, and the number before the underscore indicates the cell line and the number after indicates the biological replicate.

Document S2. Article plus Supplemental Information
