# Supplementary material for: A Human Stem Cell Model of Fabry Disease Implicates LIMP-2 Accumulation in Cardiomyocyte Pathology
Source: Stem Cell Reports. 2019 Aug 1;13(2):380–93. doi: 10.1016/j.stemcr.2019.07.004 (PMC6700557; doi:10.1016/j.stemcr.2019.07.004)
Supplement: Document S1. Supplemental Experimental Procedures, Figures S1–S5, and Tables S1, S2, and S4–S7 [file mmc1.pdf]

Stem Cell Reports, Volume *13*

Supplemental Information

A Human Stem Cell Model of Fabry Disease Implicates LIMP-2 Accumulation in Cardiomyocyte Pathology

Matthew J. Birket, Sophie Raibaud, Miriam Lettieri, Antony D. Adamson, Valerie Letang, Pauline Cervello, Nicolas Redon, Gwenaelle Ret, Sandra Viale, Bing Wang, Bruno Biton, Jean-Claude Guillemot, Vincent Mikol, John P. Leonard, Neil A. Hanley, Cecile Orsini, and Jean-Michel Itier

Figure S1

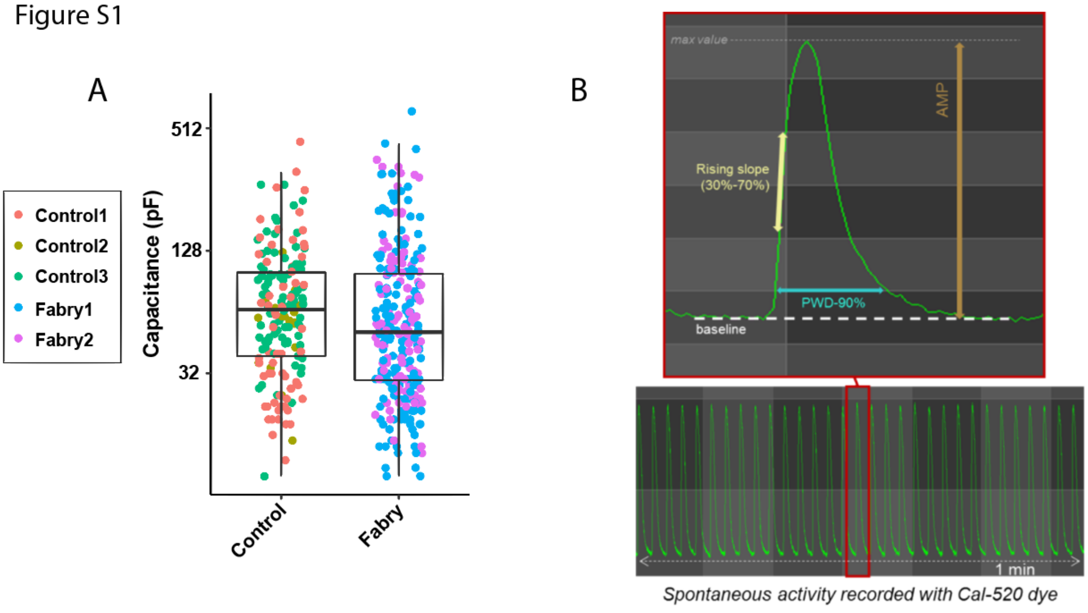
A
Control1
Control2
Control3
Fabry1
Fabry2
Capacitance (pF)
512
128
32
Control
Fabry
B
max value
AMP
Rising slope
(30%-70%)
PWD-90%
baseline
1 min
Spontaneous activity recorded with Cal-520 dye

Figure S2

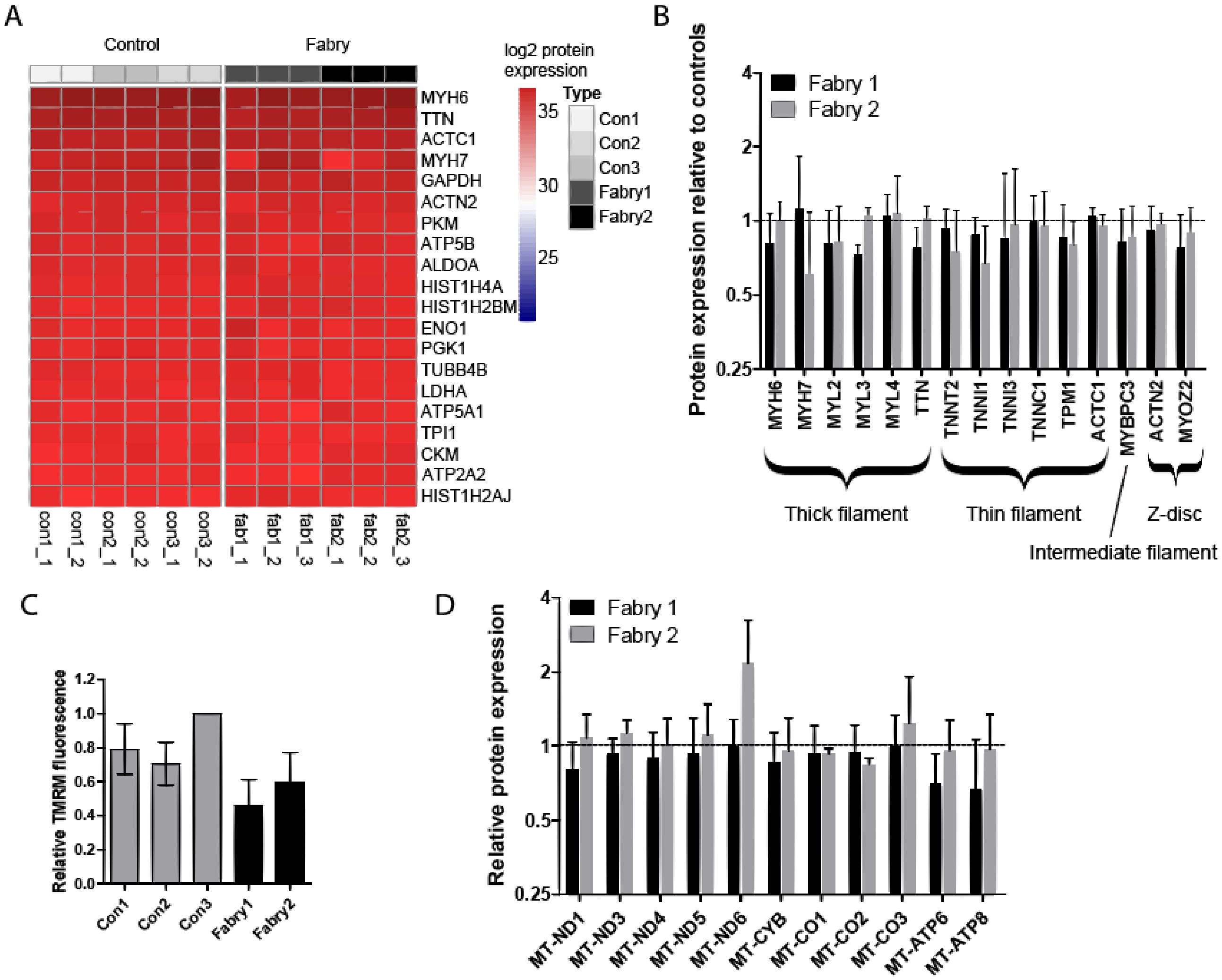
A
Control
Fabry
log2 protein expression
35
30
25
Type
Con1
Con2
Con3
Fabry1
Fabry2
MYH6
TTN
ACTC1
MYH7
GAPDH
ACTN2
PKM
ATP5B
ALDOA
HIST1H4A
HIST1H2BM
ENO1
PGK1
TUBB4B
LDHA
ATP5A1
TPI1
CKM
ATP2A2
HIST1H2AJ
con1_1
con1_2
con2_1
con2_2
con3_1
con3_2
fab1_1
fab1_2
fab1_3
fab2_1
fab2_2
fab2_3
B
Protein expression relative to controls
Fabry 1
Fabry 2
4
2
1
0.5
0.25
MYH6
MYH7
MYL2
MYL3
MYL4
TTN
TNNT2
TNNI1
TNNI3
TNNC1
TPM1
ACTC1
MYBPC3
ACTN2
MYOZ2
Thick filament
Thin filament
Z-disc
Intermediate filament
C
Relative TMRM fluorescence
1.2
1.0
0.8
0.6
0.4
0.2
0.0
Con1
Con2
Con3
Fabry1
Fabry2
D
Relative protein expression
Fabry 1
Fabry 2
4
2
1
0.5
0.25
MT-ND1
MT-ND3
MT-ND4
MT-ND5
MT-ND6
MT-CYB
MT-CO1
MT-CO2
MT-CO3
MT-ATP6
MT-ATP8

Figure S3

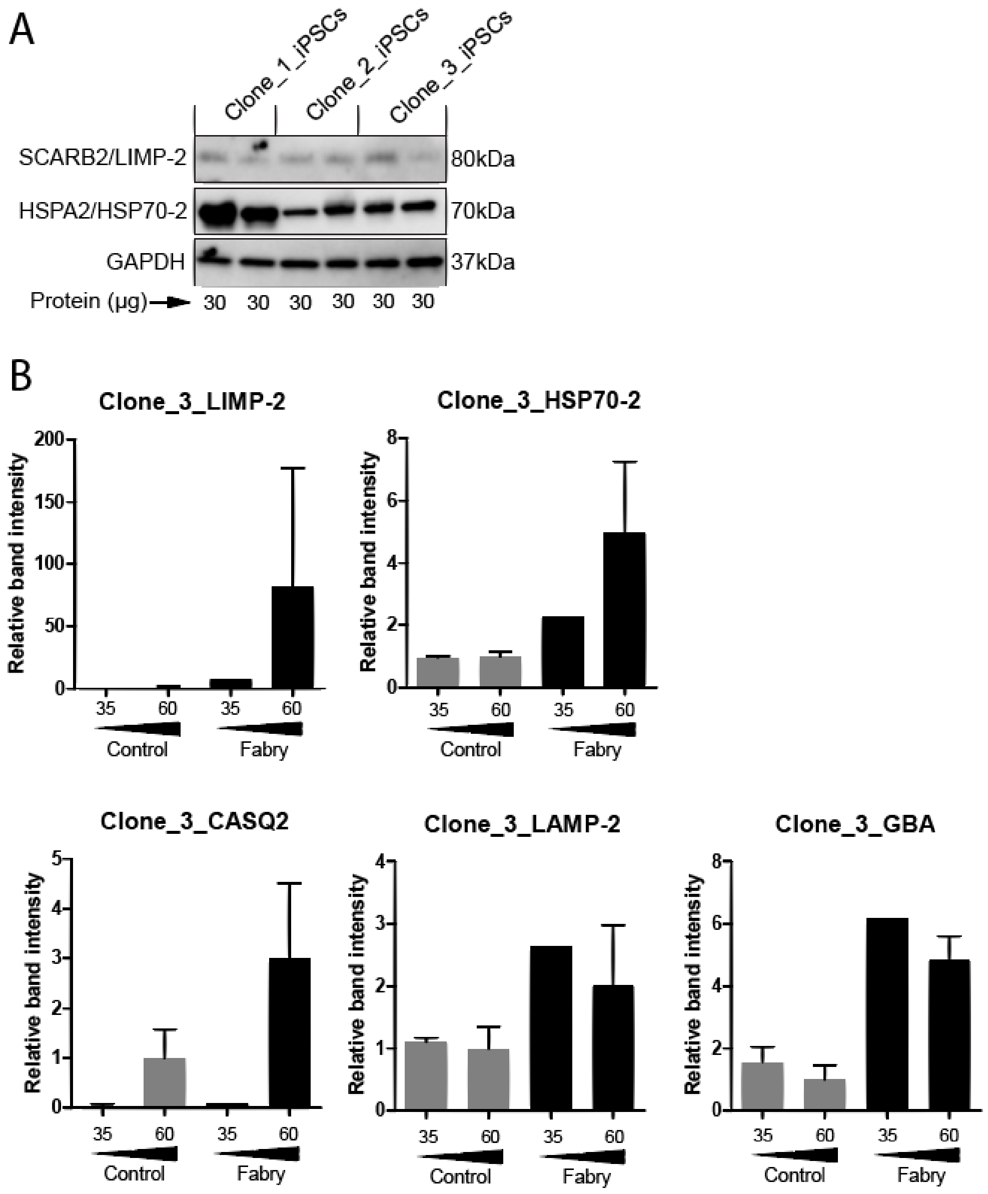
A
Clone_1_iPSCs
Clone_2_iPSCs
Clone_3_iPSCs
SCARB2/LIMP-2
HSPA2/HSP70-2
GAPDH
80kDa
70kDa
37kDa
Protein (µg)
30 30 30 30 30 30
B
Clone_3_LIMP-2
Clone_3_HSP70-2
Clone_3_CASQ2
Clone_3_LAMP-2
Clone_3_GBA
Relative band intensity
35 60 35 60
Control
Fabry

Figure S4

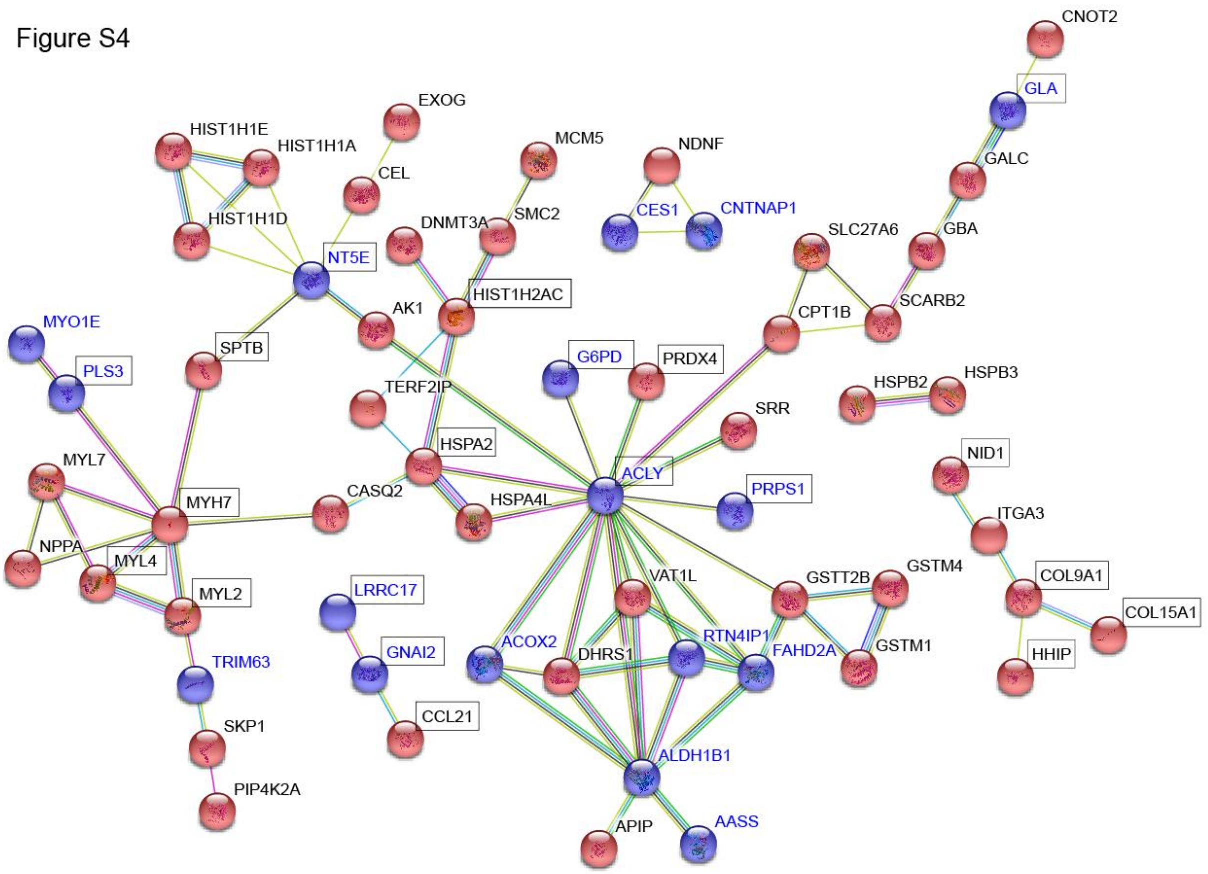
CNOT2
GLA
GALC
GBA
SCARB2
SLC27A6
CPT1B
EXOG
HIST1H1E
HIST1H1A
HIST1H1D
CEL
NT5E
MCM5
NDNF
CES1
CNTNAP1
SMC2
DNMT3A
HIST1H2AC
AK1
MYO1E
PLS3
SPTB
TERF2IP
G6PD
PRDX4
SRR
HSPB2
HSPB3
HSPA2
ACLY
PRPS1
NID1
ITGA3
MYL7
MYH7
CASQ2
HSPA4L
NPPA
MYL4
MYL2
VAT1L
GSTT2B
GSTM4
COL9A1
COL15A1
LRRC17
GNAI2
ACOX2
DHRS1
RTN4IP1
FAHD2A
GSTM1
HHIP
TRIM63
SKP1
CCL21
ALDH1B1
PIP4K2A
APIP
AASS

Figure S5

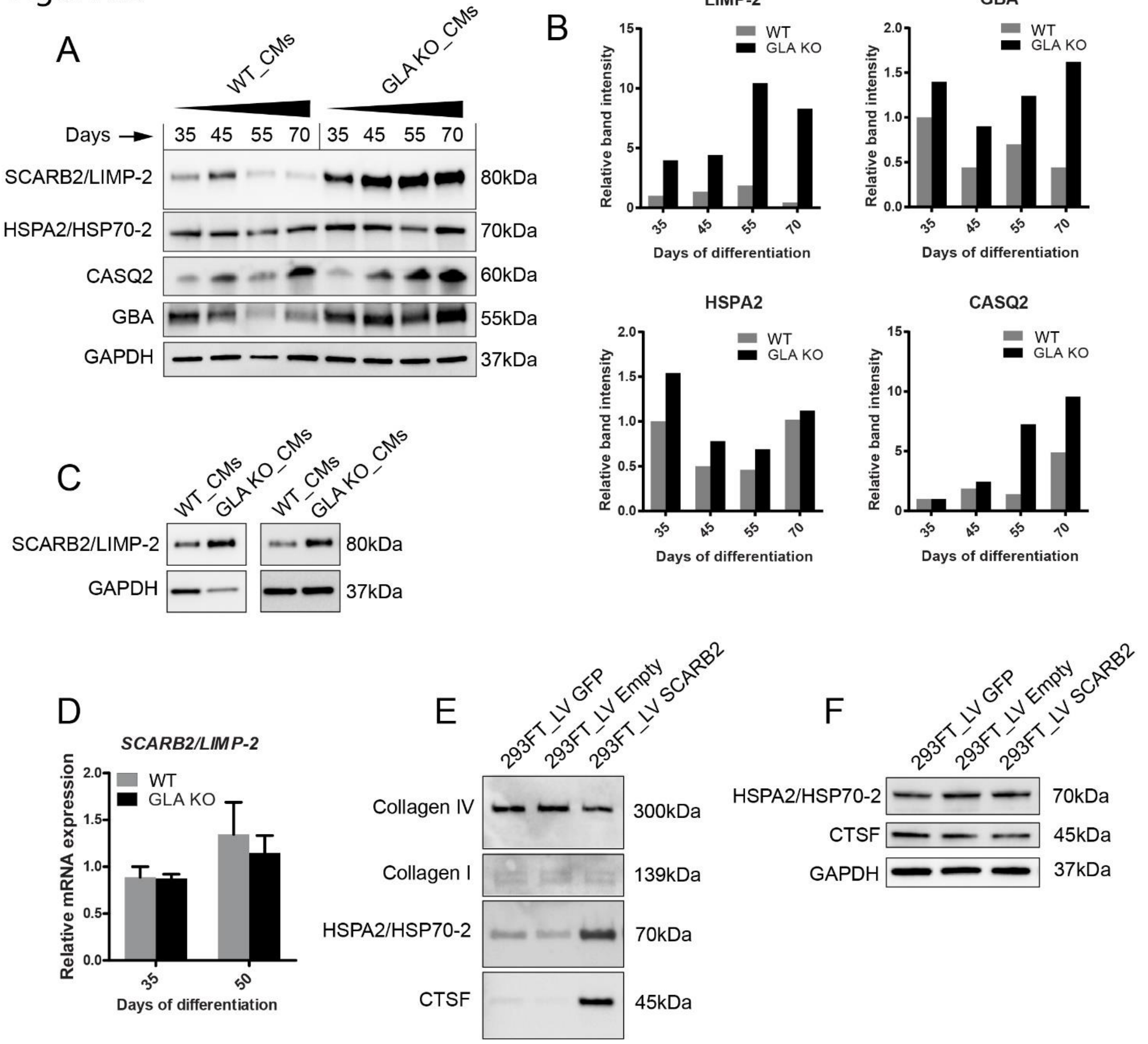
A
WT_CMs
GLA KO_CMs
Days
35 45 55 70 35 45 55 70
SCARB2/LIMP-2
80kDa
HSPA2/HSP70-2
70kDa
CASQ2
60kDa
GBA
55kDa
GAPDH
37kDa
B
LIMP-2
GBA
HSPA2
CASQ2
WT
GLA KO
Relative band intensity
Days of differentiation
C
WT_CMs
GLA KO_CMs
SCARB2/LIMP-2
80kDa
GAPDH
37kDa
D
SCARB2/LIMP-2
WT
GLA KO
Relative mRNA expression
Days of differentiation
E
293FT_LV GFP
293FT_LV Empty
293FT_LV SCARB2
Collagen IV
300kDa
Collagen I
139kDa
HSPA2/HSP70-2
70kDa
CTSF
45kDa
F
293FT_LV GFP
293FT_LV Empty
293FT_LV SCARB2
HSPA2/HSP70-2
70kDa
CTSF
45kDa
GAPDH
37kDa

Figure S1. Cardiomyocyte capacitance and calcium transient measurements. A) Capacitance measurements of single cardiomyocytes from 3 control and 2 Fabry iPSC lines. Boxes indicate the median ± quartiles and whiskers show the range minus outliers. B) Example Cal-520 imaging measurements and quantification of calcium transient parameters. PWD-90% = peak width at 90% of recovery, and AMP = amplitude (ΔF/F).

Figure S2. Extended proteome and functional analysis. A) Heatmap of the top 20 most highly expressed proteins by average abundance. B) Relative levels of sarcomeric proteins in Fabry-CMs compared to controls. The sarcomeric location of each protein is highlighted below. C) Normalized TMRM fluorescence after probe equilibration. D) Relative levels of mtDNA-encoded proteins in Fabry-CMs compared to controls. Bars show mean ± s.d. for 5 independent biological replicates for each cell line.

Figure S3. Extended Western blots and analyses. A) Western blots of three different iPSC clones derived from Fabry1 patient cells. Clone 1 is used throughout the rest of the study. B) Quantification of Western blots in **Figure 4E** as control and Fabry ordered by differentiation time, calculated as relative band density normalized to GAPDH levels. Each lysate was produced from an independent experiment. Bars show mean ± s.d.

Figure S4. Results of STRING analysis, showing potential protein-protein-interactions, as run on the 113 differentially expressed cellular and secreted proteins from **Table S3** and **Table S4**. Up- and Down-regulated proteins in Fabry-CMs are shown in red and blue respectively. Proteins differentially expressed in the secretome are highlighted in squares.

Figure S5. Extended Western blots from *GLA* KO hPSC-CMs and LIMP-2 overexpressing cells. A) Western blots of MAN13 control (WT_CMs) and paired *GLA* knockout (GLA KO_CMs) hESC-derived CMs taken at four timepoints from a single differentiation. B) Quantification of Western blots in ‘A’. C) Western blots of con3 (WT_CMs) and paired *GLA* knockout iPSC-derived CMs (day 35) from two separate experiments. D) *SCARB2* mRNA expression in MAN13 control and paired *GLA* knockout hESC-derived CMs, taken at two timepoints through a single differentiation. E) Western blot of secretome samples from two independent experiments/transductions showing the impact of LIMP-2 overexpression on protein secretion by 293FT cells. F) Western blot of 293FT cells showing no impact of LIMP-2 overexpression on cellular marker expression.

Table S1.

	Controls (n=136)	Fabry (n=213)
**MDP (mV)**	-63.6±1.2	-69.1±0.8*
**dV/dt_{max} (V/s)**	41.6±4.5	64.6±4.1*
**APA (mV)**	89.9±2.1	88.9±1.3
**APD_{90} (ms)**	476±35	276±19*
**Frequency (Hz)**	1.2±0.1	1.6±0.1*
**Capacitance (Cm)**	80.3±4.8	79.3±4.9
***I*Na (pA/pF)**	60.3±3.5	108.7±5.5*

Table S2.

	Controls (n = 8)	Fabry (n = 8)
**Frequency (Hz)**	0.8±0.1	0.9±0.2
**Peak width duration 90% (ms)**	830±8	581±7
**Corrected peak width duration 90% (ms)**	777±4	556±3
**Rising slope (30-70%)**	20±2	57±2
**Baseline fluorescence (F)**	995±7	917±7
**Amplitude (ΔF/*F*)**	2.2±0.1	3.2±0.4
**Caffeine-induced amplitude (ΔF/*F*)**	2.7±0.4	3.9±0.4

Table S4.

Uniprot	Protein name	Gene name	Log2 FC Fabry1	Log2 FC Fabry2	AvExp	p.value	adj.P.Val
Q96GE9	Transmembrane protein 261	TMEM261	4.17	4.10	21.11	1.18E-08	4.97E-05
P09488	Glutathione S-transferase Mu 1	GSTM1	3.84	4.05	21.24	9.44E-07	0.000991
Q02539	Histone H1.1	HIST1H1A	3.97	2.67	22.36	8.16E-05	0.009957
Q9HCJ6	Synaptic vesicle membrane protein VAT-1 homolog-like	VAT1L	2.73	2.91	23.57	3.65E-05	0.006139
Q13601	KRR1 small subunit processome component homolog	KRR1	2.68	2.92	20.41	7.67E-06	0.002479
P54652	Heat shock-related 70 kDa protein 2	HSPA2	3.33	2.09	25.79	1.58E-06	0.001325
Q9Y2C4	Nuclease EXOG, mitochondrial	EXOG	2.32	3.09	21.59	0.000557	0.022275
P0CG30	Glutathione S-transferase theta-2B	GSTT2B	2.03	3.10	24.49	2.87E-06	0.001471
Q5T6V5	UPF0553 protein C9orf64	C9orf64	2.77	2.17	22.45	0.000777	0.025588
Q7L5Y1	Mitochondrial enolase superfamily member 1	ENOSF1	2.49	2.06	19.92	0.001314	0.033058
Q8N1Q1	Carbonic anhydrase 13	CA13	2.07	1.86	22.39	1.79E-05	0.003694
O75764	Transcription elongation factor A protein 3	TCEA3	1.46	2.04	21.14	5.55E-05	0.007746
O95347	Structural maintenance of chromosomes protein 2	SMC2	1.58	1.93	20.44	1.90E-05	0.003694
Q5TDH0	Protein DDI1 homolog 2	DDI2	1.65	1.71	23.28	0.000422	0.019693
Q9H7C9	Mth938 domain-containing protein	AAMDC	1.60	1.48	21.99	3.49E-07	0.000732
O14958	Calsequestrin-2	CASQ2	1.14	1.91	27.71	0.002653	0.045901
Q9NZN8	CCR4-NOT transcription complex subunit 2	CNOT2	1.30	1.49	20.41	0.002426	0.044918
Q14331	Protein FRG1	FRG1	1.55	1.14	20.32	0.00116	0.030633
Q14108	Lysosome membrane protein 2	SCARB2	1.48	1.21	27.31	4.90E-06	0.001915
Q9NYB0	Telomeric repeat-binding factor 2-interacting protein 1	TERF2IP	1.25	1.43	20.24	0.002794	0.046567
Q96LJ7	Dehydrogenase/reductase SDR family member 1	DHRS1	1.68	0.94	21.09	0.000228	0.014663
Q92523	Carnitine O-palmitoyltransferase 1, muscle isoform	CPT1B	1.27	1.12	25.82	1.70E-05	0.003694
Q9UBX1	Cathepsin F	CTSF	0.77	1.61	20.80	0.001077	0.029489
Q9Y6M5	Zinc transporter 1	SLC30A1	1.09	1.23	21.43	0.002451	0.044918
P16402	Histone H1.3	HIST1H1D	1.49	0.75	23.62	0.000982	0.028905
O95757	Heat shock 70 kDa protein 4L	HSPA4L	1.53	0.57	23.73	0.000372	0.018275
P33992	DNA replication licensing factor MCM5	MCM5	1.40	0.61	25.43	0.002254	0.043628
P05114	Non-histone chromosomal protein HMG-14	HMGN1	1.24	0.71	19.43	0.000352	0.017998
P46937	Transcriptional coactivator YAP1	YAP1	0.87	1.06	22.17	0.000112	0.010898
Q53HC9	Protein TSSC1	TSSC1	1.05	0.88	21.42	0.002837	0.046689
Q9GZT4	Serine racemase	SRR	0.99	0.83	24.84	3.05E-05	0.005329
P54252	Ataxin-3	ATXN3	0.87	0.89	19.88	1.93E-05	0.003694
P29558	RNA-binding motif, single-stranded-interacting protein 1	RBMS1	1.00	0.76	21.49	0.000193	0.013737
Q01449	Myosin regulatory light chain 2, atrial isoform	MYL7	1.03	0.71	27.95	0.001927	0.040819
P04062	Glucosylceramidase	GBA	0.69	0.96	26.67	8.77E-05	0.010235

Q96FV2	Secernin-2	SCRN2	0.79	0.78	24.37	0.000151	0.012343
Q9H008	Phospholysine phosphohistidine inorganic pyrophosphate phosphatase	LHPP	0.92	0.65	23.22	0.002051	0.041287
P00167	Cytochrome b5	CYB5A	0.79	0.74	24.41	0.000157	0.012431
Q12988	Heat shock protein beta-3	HSPB3	0.88	0.63	24.91	0.00238	0.044421
Q96E39	RNA binding motif protein, X-linked-like-1	RBMXL1	0.87	0.64	23.17	0.000735	0.024936
P10412	Histone H1.4	HIST1H1E	0.98	0.50	27.81	0.001939	0.040819
Q9HA64	Ketosamine-3-kinase	FN3KRP	0.84	0.64	23.16	0.00023	0.014663
Q8TE77	Protein phosphatase Slingshot homolog 3	SSH3	0.81	0.67	20.37	3.00E-05	0.005329
Q05084	Islet cell autoantigen 1	ICA1	0.66	0.80	21.97	0.00014	0.012009
Q03013	Glutathione S-transferase Mu 4	GSTM4	0.58	0.88	25.07	0.000715	0.024806
P48426	Phosphatidylinositol 5-phosphate 4-kinase type-2 alpha	PIP4K2A	0.78	0.64	23.31	4.79E-05	0.006934
P63208	S-phase kinase-associated protein 1	SKP1	0.66	0.73	22.41	0.000235	0.014746
P54803	Galactocerebrosidase	GALC	0.77	0.62	24.16	0.000121	0.011511
Q9HBK9	Arsenite methyltransferase	AS3MT	0.65	0.73	24.81	2.48E-06	0.001471
Q9Y6K1	DNA (cytosine-5)-methyltransferase 3A	DNMT3A	0.65	0.51	23.83	0.000525	0.021418
Q7Z7K0	COX assembly mitochondrial protein homolog	CMC1	0.57	0.57	22.56	0.002471	0.044918
P00568	Adenylate kinase isoenzyme 1	AK1	0.51	0.60	28.90	0.000445	0.020309
Q16082	Heat shock protein beta-2	HSPB2	0.52	0.53	26.85	0.000173	0.012774
Q9BXW7	Cat eye syndrome critical region protein 5	CECR5	-0.54	-0.50	27.00	0.001837	0.040402
Q14249	Endonuclease G, mitochondrial	ENDOG	-0.72	-0.51	24.95	0.000108	0.010898
Q12965	Unconventional myosin-Ie	MYO1E	-0.54	-0.71	22.16	5.72E-05	0.007746
Q99424	Peroxisomal acyl-coenzyme A oxidase 2	ACOX2	-0.69	-0.64	22.05	0.000266	0.015963
Q86VU5	Catechol O-methyltransferase domain-containing protein 1	COMTD1	-0.58	-0.85	22.47	9.51E-05	0.010575
Q8WWV3	Reticulon-4-interacting protein 1, mitochondrial	RTN4IP1	-0.61	-0.87	24.57	3.15E-06	0.001471
Q969Q1	E3 ubiquitin-protein ligase TRIM63	TRIM63	-0.52	-1.01	22.53	0.000344	0.017859
**Q96GX9**	Methylthioribulose-1-phosphate dehydratase	APIP	-0.82	-0.73	23.54	0.000461	0.020309
**Q96GK7**	Fumarylacetoacetate hydrolase domain-containing protein 2A	FAHD2A	-0.73	-0.82	26.08	6.23E-07	0.000872
**P26006**	Integrin alpha-3	ITGA3	-0.69	-1.17	24.53	0.000469	0.020309
**P30837**	Aldehyde dehydrogenase X, mitochondrial	ALDH1B1	-1.34	-1.03	26.61	6.70E-06	0.002345
**Q9Y2P4**	Long-chain fatty acid transport protein 6	SLC27A6	-1.44	-1.17	26.53	0.001958	0.040819
**Q9UDR5**	Alpha-aminoadipic semialdehyde synthase, mitochondrial	AASS	-1.64	-1.09	25.38	0.00017	0.012774
**Q9HB40**	Retinoid-inducible serine carboxypeptidase	SCPEP1	-1.95	-1.16	21.23	0.000731	0.024936
**Q9BQT8**	Mitochondrial 2-oxodicarboxylate carrier	SLC25A21	-1.59	-1.76	20.43	0.000468	0.020309
**P35556**	Fibrillin-2	FBN2	-1.88	-1.61	25.84	0.000866	0.026645
**P78357**	Contactin-associated protein 1	CNTNAP1	-2.41	-1.35	24.00	0.000744	0.024993
**P06280**	Alpha-galactosidase A	GLA	-2.13	-2.59	19.60	0.000324	0.017347
**P23141**	Liver carboxylesterase 1	CES1	-3.68	-3.27	20.87	2.70E-06	0.001471

Table S5.

GO biological process complete	Homo sapiens - REFLIST (21042)	Fabry_set (72)	Fabry_set (expected)	Fabry_set (over/under)	Fabry_set (fold Enrichment)	Fabry_set (P-value)
glycosylceramide catabolic process (GO:0046477)	6	3	0.02	+	> 100	1.18E-02
metabolic process (GO:0008152)	9949	54	34.04	+	1.59	1.35E-02
Unclassified (UNCLASSIFIED)	3561	2	12.18	-	< 0.2	0.00E+00

Table S6.

GO cellular component complete	Homo sapiens - REFLIST (21042)	Fabry_set (72)	Fabry_set (expected)	Fabry_set (over/under)	Fabry_set (fold Enrichment)	Fabry_set (P-value)
lysosomal lumen (GO:0043202)	93	5	0.32	+	15.71	2.48E-02
mitochondrion (GO:0005739)	1731	19	5.92	+	3.21	5.35E-03
cytoplasm (GO:0005737)	11364	57	38.88	+	1.47	1.08E-02
intracellular part (GO:0044424)	14239	64	48.72	+	1.31	3.57E-02
Unclassified (UNCLASSIFIED)	2449	3	8.38	-	0.36	0.00E+00

Table S7.

UniProt	Protein name	Gene name	Log2 FC Fabry1	Fabry2	AvExp	p_value	adj.P.Val
**Q9UBX1**	Cathepsin F	CTSF	5.08	4.79	25.06	5.29E-07	0.000436
**O14558**	Heat shock protein beta-6	HSPB6	4.81	4.16	21.48	7.44E-07	0.000436
**P54652**	Heat shock-related 70 kDa protein 2	HSPA2	5.11	3.17	21.57	1.37E-06	0.000483
**P39059**	Collagen alpha-1(XV) chain	COL15A1	5.98	1.47	22.52	0.001189	0.043607
**P20396**	Pro-thyrotropin-releasing hormone	TRH	3.07	2.96	28.36	1.48E-05	0.004347
**P20849**	Collagen alpha-1(IX) chain	COL9A1	3.65	2.25	25.93	2.51E-05	0.006315
**O00585**	C-C motif chemokine 21	CCL21	4.32	1.37	23.98	0.000866	0.033867
**P19835**	Bile salt-activated lipase	CEL	1.14	3.12	29.08	0.000226	0.018114
**Q8N436**	Inactive carboxypeptidase-like protein X2	CPXM2	2.80	1.39	26.21	0.000931	0.035611
**Q8TB73**	Protein NDNF	NDNF	2.92	1.04	26.90	0.000128	0.017251
**Q93077**	Histone H2A type 1-C	HIST1H2AC	2.33	1.46	22.96	0.000563	0.029984
**P01160**	Natriuretic peptides A	NPPA	2.09	0.98	30.49	0.000167	0.017251
**P11277**	Spectrin beta chain, erythrocytic	SPTB	1.19	1.70	24.13	0.000487	0.028591
**P10916**	Myosin regulatory light chain 2, ventricular/cardiac muscle isoform	MYL2	0.97	1.38	28.25	0.000206	0.017892
**Q9NZN3**	EH domain-containing protein 3	EHD3	0.75	1.40	23.77	0.000267	0.01947
**P12829**	Myosin light chain 4	MYL4	0.97	1.13	29.87	0.000158	0.017251
**Q13162**	Peroxiredoxin-4	PRDX4	1.16	0.83	23.27	1.32E-06	0.000483
**Q9H3G5**	Probable serine carboxypeptidase CPVL	CPVL	0.66	1.31	27.91	0.000574	0.029984
**P14543**	Nidogen-1	NID1	1.11	0.57	30.64	0.000175	0.017251
**P12883**	Myosin-7	MYH7	0.70	0.92	30.83	0.001367	0.047176
**P60891**	Ribose-phosphate pyrophosphokinase 1	PRPS1	-0.78	-0.62	23.82	0.001496	0.049663
**Q16643**	Drebrin	DBN1	-0.53	-0.95	24.70	0.001313	0.046801
**P04899**	Guanine nucleotide-binding protein G(i) subunit alpha-2	GNAI2	-0.63	-0.86	23.88	4.26E-05	0.009374
**P31949**	Protein S100-A11	S100A11	-0.87	-0.94	27.48	0.000772	0.031604
**P53396**	ATP-citrate synthase	ACLY	-0.75	-1.10	26.19	0.000211	0.017892
**P13797**	Plastin-3	PLS3	-0.97	-1.24	27.69	0.000975	0.036496
**P11413**	Glucose-6-phosphate 1-dehydrogenase	G6PD	-1.59	-1.60	25.12	0.000286	0.01947
**Q13219**	Pappalysin-1	PAPPA	-1.33	-1.86	27.65	0.000417	0.025335
**P21589**	5-nucleotidase	NT5E	-3.50	-1.88	21.19	0.000687	0.031
**Q8IWU6**	Extracellular sulfatase Sulf-1	SULF1	-3.00	-2.44	22.75	0.000715	0.031478
**Q9UHG2**	ProSAAS	PCSK1N	-1.91	-3.58	20.59	0.000176	0.017251
**Q96QV1**	Hedgehog-interacting protein	HHIP	-2.47	-3.05	22.14	0.000684	0.031
**Q8N6Y2**	Leucine-rich repeat-containing protein 17	LRRC17	-2.45	-4.07	22.08	0.000283	0.01947
**P06280**	Alpha-galactosidase A	GLA	-4.52	-4.27	20.51	5.39E-09	9.48E-06

Table S1. Action potential parameters and sodium current of single spontaneously active cardiomyocytes. Data are mean ± SEM; n = number of cells; MDP = maximal diastolic potential; dV/dt_{max} = maximal upstroke velocity; APA = action potential amplitude; APD_{90} = action potential duration at 90% repolarization; *I*Na = peak sodium current normalized by capacitance. * $p < 0.05$ unpaired t-test.

Table S2. Calcium transient parameters and response to caffeine. Data are mean ± SEM; n = number of experimental wells measured.

Table S3. Complete cardiomyocyte proteome and secretome data in excel format. Tab 1 (‘Exp1_proteome’) shows data from the first cell proteomics experiment and tab 2 (‘Exp2_proteome’) shows data from the second experiment. Tab 3 (‘Exp1_secretome’) shows data from the first secretome experiment, tab 4 (‘Exp2_secretome’) shows data from the second secretome experiment and tab 5 (‘Exp3_secretome’) shows data from the third secretome experiment. Protein levels are shown as log2 values. AvExp = the mean expression across all samples in the run. For sample names, con = control lines, and fab = Fabry lines, and the number before the underscore indicates the cell line and the number after indicates the biological replicate.

Table S4. Differentially expressed proteins from complete cardiomyocyte proteome data identified using a filtering approach. Log2FC = log2 fold-change of Fabry samples (mean of 5 biological replicates for each line) compared to controls; AvExp = average expression across all samples as log2 value; t = t statistic; adj.P.Val = p value with Benjamini-Hochberg adjustment.

Table S5. Gene ontology enrichment analysis 2: cellular component overrepresentation test results. The 72 differentially expressed proteins from **Table S4** were analysed using PANTHER (Mi et al., 2017) to test for cellular component enrichment against the whole genome.

Table S6. Gene ontology (GO) enrichment analysis 1: biological process overrepresentation test results. The 72 differentially expressed proteins from **Table S4** were analysed using PANTHER (Mi et al., 2017) to test for process enrichment against the whole genome.

Table S7. Differentially expressed proteins from complete cardiomyocyte secretome data identified using a filtering approach. Log2FC = log2 fold-change of Fabry samples compared to controls (mean of 7 biological replicates for each line); AvExp = average expression across all samples as log2 value; t = t statistic; adj.P.Val = p value with Benjamini-Hochberg adjustment.

Extended Experimental Procedures

hPSC culture and cardiac differentiation

The derivation of iPSC lines from two male Fabry disease patients carrying non-sense mutations in *GLA* was previously described (Itier et al., 2014). The patient fibroblasts were obtained from the Coriell Institute for Medical Research. In this article, clone GM00107 (c.485G>A; W162X) is referred to as Fabry 1 and clone GM00881 (c.658C>T; R220X) as Fabry 2. Four healthy male control iPSC lines were also derived internally from skin fibroblasts using the same protocol. MAN13 hESCs were kindly provided by the Kimber lab (Ye et al., 2017). MAN13 hESCs are a UK Stem Cell Bank registered line and this study was performed in full compliance with the laws, regulations and procedures that govern hESC use in the UK. The study conducted is in full compliance with Sanofi guidelines on the use of hESCs. hPSC lines were maintained in mTeSR medium (Stem Cell Technologies), on Matrigel (Corning) and passaged weekly using ReLeSR (Stem Cell Technologies). For cardiac differentiation cultures were expanded for 6 days to near confluence and then treated as follows: 10 ng/ml BMP4 + 4 µM CHIR991021 (Miltenyi Biotec) for 48 hours and then 5 µM Wnt C-59 (Selleckchem) for 48 hours, in RPMI 1640 medium containing 213 µg/ml L-ascorbic acid 2-phosphate, 500 µg/ml recombinant human albumin (A9731 Sigma-Aldrich), 100 pg/ml bFGF (Miltenyi Biotec) + 1:100 000 ITS-X (Thermo Fisher), or in RPMI 1640 + insulin-free B27. Cells were then maintained in RPMI + bFGF and ITS-X until day 11 of differentiation when they were dissociated with TrypLE Select (Thermo Fisher) and transferred to suspension in ultra-low attachment plates (Corning, #3471) for 3 days. For suspension culture and plating, cells were maintained in cardiomyocyte medium (CM): DMEM/F12 + 0.25% Bovine Serum Albumin + 1 mM Creatine, 5 mM Taurine, 0.5 mM L-carnitine, 50 µg/ml α-MTG, 50 µg/ml AA-2P, 1x Trace Elements A, B and C (Corning), 1x Chemically Defined Lipids (Thermo Fisher), 1x Glutamax, 0.5x pen/strep and 1 µM Thiazovivin (Stemgent), plus the addition of 1% FCS. On day 14 of differentiation the aggregated cardiomyocytes were purified using a MACS column based PSC-Derived Cardiomyocyte Isolation Kit (Miltenyi, #130-110-188) and replated on Matrigel-coated plastic. After two days the medium was changed to a maturation promoting formula: CM (as above) + 5 nM T3 + 5 nM dexamethasone + 20 µM palmitic acid (conjugated to fatty-acid free BSA) + 0.25% FBS, as supported by our previous work (Birket et al., 2015). Unless otherwise stated, reagents were from Sigma-Aldrich. Cardiomyocytes were analysed between day 35 and day 60 of differentiation and later dissociations were performed with TrypLE Select (Thermo Fisher).

CRISPR-Cas9 gene editing

We initially attempted to directly convert the *GLA* c.658C>T using a sgRNA targeting this site and supplying a ssDNA donor harbouring the correction flanked by 60nt homology. However, despite high incidence of NHEJ no sequence correction was detected (measured by TIDE/TIDER analysis (Brinkman et al., 2014); data not shown). We thus adopted a selection based CRISPR strategy. First, ribonucleoprotein (RNP) complexes of

CRISPR-Cas9 targeting sequences flanking exon 5, along with a targeting vector, were delivered into Fabry2 iPSCs using an Amaxa 2b nucleofector (program B-16). The crRNA sequences were as follows: 5'-ACAGATTGAACGTCTCCATA-3' and 5'-TATGTGAGAGTACTTAGAGC-3'. RNP complexes were made by complexing 120 pmol cr:tracRNA duplex (IDT) with 67 pmol S.p. Cas9 Nuclease V3 (IDT). 120 pmol carrier DNA was included as an electroporation enhancer (IDT cat # 1075915).

The targeting vector incorporated a 5' homology arm of 801 bp and a 3' homology arm of 750 bp flanking corrected exon 5 (pUC57-5'H-loxP-SA-2A-Puro-loxP-correctExon-3'H). 48h after electroporation, iPSCs were selected with 1 µg/ml puromycin for 48h, then electroporated with 1 µg of Cre expression vector (pCAG-Cre-IRES2-GFP; Addgene plasmid #26646), FACS sorted for GFP expression and directly cloned. Expanded clones were screened by PCR, taking advantage of a unique intronic primer site introduced 3' of exon 5, and sequenced. All analyzed clones were correctly targeted.

To generate *GLA* knockout hPSC lines, MAN13 hESCs and con 3 iPSCs were electroporated as above using a crRNA sequence targeting exon 1 (5'-TTGGCAAGGACGCCTACCAT-3'), which was found to induce in-del mutations with 60-80% efficiency. Cells were cloned by serial dilution and screened by sequencing. Clones with frameshift mutations and undetectable α-gal A expression by Western blot were used for subsequent experiments: MAN13 hESCs (*GLA* c.121_125delTACCA) and con3_iPSCs (*GLA* c. 123delC).

Lentiviral transduction

SCARB2 was overexpressed by lentivirus produced using a pLenti CMV/TO Puro DEST vector (Addgene plasmid #17452). The empty vector, or vector with a GFP insert, were used as controls. Lentiviral particles were concentrated using ultracentrifugation and used to transduce cardiomyocytes at the time of their purification on day 14. Transduced cells were selected with 0.75 µg/ml puromycin and then cultured as normal. Conditioned medium was collected 5-8 days after transduction of cardiomyocytes or 293FT cells.

Gb3 quantification

Measurements were performed using mass spectrometry as previously described (Itier et al., 2014).

Flow cytometry

Differentiated cultures were dissociated using TrypLE Select and labelled with a primary biotin-conjuated anti-CD172a (SIRPA) antibody (Miltenyi-biotec; clone REA144) and then an anti-biotin-VioBright FITC secondary antibody (Miltenyi-biotec; #130-104-514). For TMRM measurements, cells were equilibrated with 2.5nM TMRM overnight before dissociation and measurement. Measurements were performed on a Miltenyi VYB.

Immunocytochemistry and cell size determination

Dissociated iPSC-CMs differentiated for ~40 days were seeded at single cell density on glass coverslips micropatterned with 20 µm wide gelatin lines as previously described (Birket et al., 2015). 4 days later cells

were fixed with 4% paraformaldehyde for 20 mins, permeablized with 0.1% Triton-X, blocked with 1% BSA + 0.4% horse serum for 1 hour, and then labelled overnight at 4°C with primary antibody. Antibodies were as follows: anti-α-actinin (Sigma; A7811), anti-MLC-2V (Proteintech; 10906-1-AP) and anti-myomesin-3 (Proteintech; 17692-1-AP). Imaging was performed with a 60x objective. Individual separated cardiomyocytes were identified based on α-actinin labelling and then MLC-2V or myomesin-3 labelling was also imaged and recorded. Cell area was calculated using a thresholding mask based on α-actinin labelling.

Electrophysiology

Dissociated iPSC-CMs differentiated for 30 days were seeded at single cell density on glass coverslips (Corning, #354086) coated with fibronectin (Sigma, #F0895) and used for measurement 3-8 days later. Patch-clamp experiments were performed at 37°C and cells were continuously perfused with an external solution containing (in mM): 140 NaCl, 5 KCl, 1 $CaCl_2$, 1 $MgCl_2$, 10 glucose, 10 HEPES. pH was set to 7.4 with NaOH 1M. The patch pipettes were pulled from thick-walled borosilicate glass capillaries (Harvard Apparatus, Edenbridge, UK) on a DMZ-universal electrode puller (Zeitz-Instruments GmbH). Electrode impedance was 4–6 MOhms when filled with an internal solution containing (in mM): 110 Kaspartate, 20 KCl, 1 $MgCl_2$, 0.1 Na-GTP, 5 Mg-ATP, 5 phosphocreatine, 1 EGTA, 10 HEPES. pH was adjusted to 7.2 with KOH 1 M. Data were all corrected for liquid junction potentiel (15.4 mV). Ionic currents and action potentials were recorded on isolated spontaneously beating CMs in the whole-cell configuration of the patch-clamp technique using digidata 1550/Multiclamp 700B (Molecular Devices, Sunnyvale, CA, USA) for data amplification and acquisition. Data were acquired at 10 KHz and low-pass filtered at 5 kHz using Clampex software (pClamp 10.5, Molecular Devices). The same following protocol was applied to all CMs: upon seal formation and following patch membrane break, a 50 ms test pulse from a holding potential of -80 to 0 mV was applied in voltage–clamp mode to record Na^+-current maximal amplitude. The patch was then switched to current–clamp mode and spontaneous action potentials (APs) were recorded for 1 min at resting potential without injecting any current.

For currents and AP analysis, Clampfit software was used (pClamp 10.5, Molecular Devices). Na^+-current density was obtained by normalizing Na^+-current maximal amplitude to the cell capacitance and expressed in pA/pF. For AP analysis, common electrophysiological characteristics such as action potential duration at 90% of repolarization (APD90), action potential amplitude and maximum diastolic potential (MDP) were calculated.

Calcium imaging

Dissociated iPSC-CMs differentiated for 30 days were seeded at a density of 5 x 10^4 cells/well in black walled clear bottom 96-well plates (Costar, #655946) coated with Matrigel and used for measurement 3-8 days later. The Cal-520 calcium assay was performed as outlined in the product information sheet provided with the Hamamatsu FDSS *µCell* (Hamamatsu Photonics, Hamamatsu, Japan). Briefly, stock solution of Cal-520 dye was

prepared at 5 mM in DMSO. Assay buffer was made from Hank's Balanced Saline Solution (HBSS) buffered with 20 mM HEPES. Powerload™ concentrate was used to enhance the dye solubility. Cells were incubated in 5 µM of Cal-520 dye for 60 minutes to 90 minutes at 37°C, washed twice with warm dye-free assay buffer and then imaged in 80 µL per well of warm assay buffer. Fluorescence measurements were performed into FDSS *µCell* at 37°C. The microplate was illuminated at 470 nm and fluorescence was measured at 540 nm using the high-speed acquisition mode (100 Hz). The caffeine response was assessed by injecting 10 µl of caffeine at 50 mM using the 96-well dispenser head, resulting in a final concentration of 10 mM. Data acquisition was performed using FDSS*v3* software and analyzed using the WaveForm Analysis, software (Hamamatsu Photonics, Hamamatsu, Japan). Fluorescence measurements are represented as relative fluorescence unit (RFU). For each experiment, spontaneous calcium transients were analyzed over 2 minutes. Frequency, basal fluorescence (F), amplitude (ΔF/F), peak width at 90% of recovery (PWD-90%), corrected peak width (corrected PWD-90%, calculated by Fridericia's formula and rising slope (30%-70%) were measured (exemplified in **Fig. S1**).

Proteome sample preparation

Dissociated iPSC-CMs differentiated for 35 days were pelleted and lysed in a buffer of 1% SDS, 0.25 u/mL benzonase and protease inhibitors for 30 min at 37°C. Protein concentration was measured with BCA (Thermo BCA Protein Assay Kit). Extracted protein was then loaded on a 4-12% SDS-page gel (approx. 12 µg protein per lane) for approx. 3 min. Reduction/alkylation/digestion steps were then performed in gel. Gel slices were subjected to two washing steps with a mixture of 50 mM ammonium carbonate buffer/ACN (50/50), then to reduction by 30 min incubation at 56°C with 20 mM DTT, followed by two more washing steps. Proteins were subjected to alkylation for 30 min in presence of 40 mM iodoacetamide, followed by two washing steps. After the addition of 0.01 µg/µL trypsin, gels slices were incubated overnight at 37°C. The peptide mixture solution was then acidified by addition of formic acid and filtered using a 0.2 µm membrane before injection.

Secretome sample preparation

Cardiomyocytes were washed 3 times and then incubated in FCS- and BSA-free medium for conditioning and collection. The medium composition was: phenol red-free DMEM/F12 + 1:100 000 ITS-X + 1 mM Creatine, 5 mM Taurine, 0.5 mM L-carnitine, 50 µg/ml α-MTG, 50 µg/ml AA-2P, 1x Trace Elements A-B-C, 1x Chemically Defined Lipids, 1x Glutamax, 0.5x pen/strep. Conditioned medium was collected daily for up to 5 days, centrifuged at 1000g for 10 min to remove debris and frozen at -80°C.

Enrichment of soluble proteins was performed using NanoZeolites obtained from NanoScape AG; Germany. Adsorption of proteins on the surface was carried out by incubating the conditioned medium with NanoZeolite beads for 90 min at 4°C. After centrifugation at 16000g for 20 min, 4°C, pellets were washed twice with 100 mM ammonium carbonate buffer. Proteins bound to the NanoZeolite beads were reduced with 10 mM DTT,

0.05% AALS in 50 mM ammonium carbonate buffer at 56°C for 30 min and subsequently alkylated by addition of 20 mM iodoacetamid and 30 min incubation step at room temperature. Over-alkylation was limited by adding 20 mM DTT. Trypsin was then added at 0.01 µg/µL, followed by an overnight incubation at 37°C. After digestion AALS was precipitated in presence of 1% TFA and filtration performed at 0.2 µm.

For Western blotting, protein from 3ml of conditioned medium was precipitated using ice cold acetone (1:4), incubated for 1 h, 4°C, pelleted at 3500G for 30 min, 4°C, resuspended in loading buffer, heated at 95°C for 5 min and centrifuged again to remove debris.

Liquid chromatography-mass spectrometry

Peptides were separated on a nanoacquity nanoLC (Waters) coupled to an Orbitrap Fusion mass spectrometer (Thermo) via a nanoelectrospray source. Peptides were loaded on a trap column (Symetry C18 100A 180 µm x 2 cm 5 µm, Waters) and separated on a BEH C18 column (Peptide BEH-C18 130A, 1.7 µm, 75 µm x 250 mm column temperature: 40°C, Waters) by a 140 min gradient from 5% to 85% ACN (+0.1% formic acid) at a flow rate 0.3 µl/min. The Fusion was operated in a data dependent mode with a survey scan range of 325-1200 m/z and a resolution of 120 000 at m/z 200. Selection of precursors was performed using the top speed mode and subjected to high energy collision in the HCD cell at a normalized collision energy of 23. Fragmentation spectra were acquired in the trap in the rapid mode scan rate. Dynamic exclusion of sequenced peptides was set to 50 s to reduce repeated peptide sequencing. Threshold for ion injection time and ion target were set to 50 ms and 2E5 for the survey scans and 35 ms and 1E4 for the MS/MS scans respectively.

Label free quantification

MaxQuant software (version 1.5.3.8) was used to analyse MS raw files. MS/MS spectra were searched against the human Uniprot FASTA database (version February 2017). Cysteine carbamidomethylation was applied as fixed and N-terminal acetylation, pyroQ and methionine oxidations as variable modifications. Enzyme specificity was set to Trypsin/P with a maximum of 3 missed cleavages. A false discovery rate (FDR) of 1% was applied at the peptide and protein level. Normalization of samples intensities was performed via Maxquant label free quantification algorithm (MaxLFQ).

Proteomics data analysis

Data analysis and visualization was performed using the Perseus software (version 1.5.5.3) and then using R/Bioconductor tools. Proteins identified with ≥1 unique peptide and positive gene identification were used for quantification. Logarithmic transformation was applied, and the median of injections was calculated. Missing values were imputed with the mean of the minima of all data. Protein identifications were required to have more than 50% valid values in at least one group of biological replicates.

Heatmaps and hierarchical clustering plots were generated using the 'pheatmap' package. Limma analysis was performed contrasting control and Fabry protein sets with >1 unique peptide with the following arguments for the topTable() function: adjust="BH", lfc = 0.585 and p.value = 0.1 (Ritchie et al., 2015). Outputs were also visualised using the volcanoplot() function. A filtering approach to identify differentially abundant proteins was also applied by first calculating the difference in expression of each Fabry sample compared to the mean of all the control samples in that group. Proteins with ≥0.5 log2 fold-change for both Fabry lines individually and an adjusted p-value <0.05 using all samples (one-sample t-test; μ = 0) were selected. Each sample was considered as an independent replicate. The lower p-value used for filtering was used to prioritize the candidates to a sensible number. Proteins with more than one missing value in the higher expressed group were discounted, after inspection. Classification as plasma protein and presence of signal peptide was performed using the Plasma Proteome Database (http://www.plasmaproteomedatabase.org/) and SignalP (http://www.cbs.dtu.dk/services/SignalP/) via the Human Protein Atlas (https://www.proteinatlas.org/). Protein-protein interaction network analysis was performed using STRING (https://string-db.org).

Western blot

Dissociated cell pellets were lysed in RIPA buffer + protease inhibitors (Thermo Scientific; #78440) + 80U/ml DNase I (Sigma, #D4527) + 5mM EDTA, sonicated briefly and then centrifuged to remove cell debris. 10 or 30 µg of cell lysate was heated at 95°C in loading buffer, loaded onto a 4-20% SDS-polyacrylamide gel (Biorad; #5678094) and transferred to a PVDF membrane using a Trans-Blot Turbo Transfer Pack (Biorad; #170-4157). Membranes were blocked in 5% milk in TBS-T and incubated in primary antibody overnight at 4°C. Antibodies and their dilutions were as follows: Anti-LIMP-2 (Abcam ab176317; 1:1000), anti-HSP70-2 (Abcam ab108416; 1:1000), anti-calsequestrin 2 (Proteintech 18422-1-AP; 1:1000), anti-GCase (internal; 1:500), anti-LAMP-2 (Santa-Cruz Sc-18822; 1:500), anti-GLA (Abcam ab168341; 1:1000), anti-CTSF (Abcam ab200650; 1:500), anti-collagen I (SouthernBiotech 1310-01; 1:1000), anti-collagen IV (Novocastra NCL-COLL-IV; 1:1000), anti-GAPDH (Abcam; 1:3000). HRP-conjugated secondary antibodies were as follows: anti-mouse IgG(H+L) HRP (Thermo Fisher #32230; 1:10 000) and anti-rabbit IgG(H+L) HRP (Thermo Fisher #32460; 1:10 000).

References

Birket, M.J., Ribeiro, M.C., Kosmidis, G., Ward, D., Leitoguinho, A.R., van de Pol, V., Dambrot, C., Devalla, H.D., Davis, R.P., Mastroberardino, P.G., et al. (2015). Contractile Defect Caused by Mutation in MYBPC3 Revealed under Conditions Optimized for Human PSC-Cardiomyocyte Function. Cell Rep. *13*, 733.

Brinkman, E.K., Chen, T., Amendola, M., and van Steensel, B. (2014). Easy quantitative assessment of genome editing by sequence trace decomposition. Nucleic Acids Res. *42*, e168–e168.

Itier, J.-M., Ret, G., Viale, S., Sweet, L., Bangari, D., Caron, A., Le-Gall, F., Bénichou, B., Leonard, J., Deleuze, J.-F., et al. (2014). Effective clearance of GL-3 in a human iPSC-derived cardiomyocyte model of Fabry disease. J. Inherit. Metab. Dis. *37*, 1013–1022.

Mi, H., Huang, X., Muruganujan, A., Tang, H., Mills, C., Kang, D., and Thomas, P.D. (2017). PANTHER version 11: expanded annotation data from Gene Ontology and Reactome pathways, and data analysis tool enhancements. Nucleic Acids Res. *45*, D183–D189.

Ritchie, M.E., Phipson, B., Wu, D., Hu, Y., Law, C.W., Shi, W., and Smyth, G.K. (2015). limma powers differential expression analyses for RNA-sequencing and microarray studies. Nucleic Acids Res. *43*, e47.

Ye, J., Bates, N., Soteriou, D., Grady, L., Edmond, C., Ross, A., Kerby, A., Lewis, P.A., Adeniyi, T., Wright, R., et al. (2017). High quality clinical grade human embryonic stem cell lines derived from fresh discarded embryos. Stem Cell Res. Ther. *8*, 128.
