# Supplementary material for: A Human Stem Cell Model of Fabry Disease Implicates LIMP-2 Accumulation in Cardiomyocyte Pathology
Source: Stem Cell Reports. 2019 Aug 1;13(2):380–93. doi: 10.1016/j.stemcr.2019.07.004 (PMC6700557; doi:10.1016/j.stemcr.2019.07.004)
Supplement: Document S2. Article plus Supplemental Information [file mmc5.pdf]

Stem Cell Reports
Article
OPEN ACCESS

A Human Stem Cell Model of Fabry Disease Implicates LIMP-2 Accumulation in Cardiomyocyte Pathology

Matthew J. Birket,[1,2,*] Sophie Raibaud,[4] Miriam Lettieri,[2] Antony D. Adamson,[2] Valerie Letang,[4] Pauline Cervello,[4] Nicolas Redon,[4] Gwenaelle Ret,[1] Sandra Viale,[1] Bing Wang,[5] Bruno Biton,[4] Jean-Claude Guillemot,[4] Vincent Mikol,[4] John P. Leonard,[6] Neil A. Hanley,[2,3] Cecile Orsini,[1] and Jean-Michel Itier[1,*]

[1]Sanofi, Translational Sciences Unit, Sanofi, 13 quai Jules Guesdes, 94400 Vitry-sur-Seine, France
[2]Faculty of Biology, Medicine and Health, Manchester Academic Health Sciences Centre, The University of Manchester, Oxford Road, Manchester M13 9PT, UK
[3]Endocrinology Department, Manchester University NHS Foundation Trust, Grafton Street, Manchester M13 9WU, UK
[4]Sanofi, Translational Sciences Unit, Avenue Pierre Brossolette, 91380 Chilly-Mazarin, France
[5]Sanofi, GBD-Analytical R&D, 211 Second Avenue, Waltham, MA 02451, USA
[6]Sanofi, Rare Disease Science Unit, 153 Second Avenue, Waltham, MA 02451, USA
*Correspondence: matthew.birket@manchester.ac.uk (M.J.B.), jean-michel.itier@sanofi.com (J.-M.I.)
https://doi.org/10.1016/j.stemcr.2019.07.004

SUMMARY

Here, we have used patient-derived induced pluripotent stem cell (iPSC) and gene-editing technology to study the cardiac-related molecular and functional consequences of mutations in *GLA* causing the lysosomal storage disorder Fabry disease (FD), for which heart dysfunction is a major cause of mortality. Our *in vitro* model recapitulated clinical data with FD cardiomyocytes accumulating GL-3 and displaying an increased excitability, with altered electrophysiology and calcium handling. Quantitative proteomics enabled the identification of >5,500 proteins in the cardiomyocyte proteome and secretome, and revealed accumulation of the lysosomal protein LIMP-2 and secretion of cathepsin F and HSPA2/HSP70-2 in FD. Genetic correction reversed these changes. Overexpression of LIMP-2 directly induced the secretion of cathepsin F and HSPA2/HSP70-2, implying causative relationship, and led to massive vacuole accumulation. In summary, our study has revealed potential new cardiac biomarkers for FD, and provides valuable mechanistic insight into the earliest pathological events in FD cardiomyocytes.

INTRODUCTION

Fabry disease (FD) is an inherited lysosomal storage disorder (LSD) caused by mutations in the *GLA* gene on the X chromosome, leading to the deficiency of α-galactosidase A (α-gal A). FD is rare, but LSDs as a group have an incidence of approximately 1:5,000–1:10,000, and may share overlapping pathological mechanisms (Ferraz et al., 2014; Platt, 2014). FD is characterized by the progressive intracellular accumulation of globotriaosylceramide (GL-3) throughout the body, particularly in the vascular tree, nervous system, kidney, and heart (Nagueh, 2014). Most patients develop cardiac involvement, with common manifestations including left ventricular hypertrophy, arrhythmias and conduction disturbances (Linhart and Elliott, 2007). With disease progression, extensive myocardial fibrosis and impaired left ventricular function can develop, and cardiac disease has become the main cause of mortality in FD (Mehta et al., 2009). Enzyme replacement therapy has provided a major therapeutic advance. However, in the heart, clearance of GL-3 from cardiomyocytes (CMs) has not been demonstrated, in contrast to the robust clearance from endothelial cells (Eng et al., 2001; Thurberg et al., 2009).

A major obstacle for advancing therapy for patients with FD is the knowledge gap between the direct molecular consequences of α-gal A deficiency in CMs and the cascade of events driving disease in the heart; the inaccessibility of CMs from patients precludes adequate investigation of these events, especially at early stages. Whether intrinsic myocardial dysfunction drives disease progression and is a primary cause of the electrophysiological and contractile disturbances remains unclear, but is supported by the following evidence: electrocardiogram abnormalities such as a shorter P wave duration, PR interval, and QRS complex indicate an acceleration in both atrial and ventricular conduction in early-stage FD (Namdar et al., 2011), implying that GL-3 accumulation may alter cellular conductive properties; and mechanical defects have been reported in a rare example where patient CMs have been available for *in vitro* investigation (Chimenti et al., 2008).

Recently, our group reported the generation of induced pluripotent stem cells (iPSCs) from patients with FD carrying non-sense mutations in *GLA* (Itier et al., 2014). This model presents the possibility, for the first time, to systematically study the consequences of α-gal A deficiency in CMs at the molecular and functional level and to identify new disease biomarkers. Biomarkers could provide valuable direction for mechanistic investigation and help elucidate the pathological cascades in FD; systemic biomarkers could have further utility for monitoring disease status, response to therapy, and for identifying patients at high risk of cardiac complications.

 © 2019 The Authors.
This is an open access article under the CC BY license (http://creativecommons.org/licenses/by/4.0/).

Using a carefully standardized iPSC cardiac differentiation model, here we provide quantitative data at proteome scale on the cellular phenotype of CMs carrying *GLA* mutations (Fabry CMs). We have identified functional differences in these cells that are consistent with clinical data, and discovered several novel cellular and secreted protein biomarkers. The accumulation of LIMP-2, a lysosomal protein with suggested roles in heart disease, was a robust consequence of α-gal A deficiency, and could drive protein secretion, implying a fundamental role in FD pathology. These new data support the exciting potential of this model for delivering translational benefit for patients with FD.

RESULTS

Functional and Structural Characterization of Control and Fabry iPSC-Derived CMs Reveals Increased Excitability in Fabry CMs

iPSCs derived from two patients with FD carrying mutations in *GLA* (Fabry1, c.458G > A; and Fabry2, c.658C > T) that cause classic FD associated with <1% normal α-gal A activity (Altarescu et al., 2001; Maki et al., 2004) were subjected to a cardiac differentiation protocol in monolayer format along with three unrelated wild-type controls.

Robust CM generation (>40% SIRPA^{+}) was achieved for all control and disease lines using small molecules targeting the WNT pathway, combined with early BMP4 addition. CMs were enriched using a suspension culture step (Nguyen et al., 2014), and then purified by magnetic column/antibody-bead conjugates and returned to a monolayer; final purity was >95% by SIRPA labeling (Figure 1A; Video S1). α-Gal A deficiency was confirmed in Fabry CMs: by day 28 GL-3 levels were ∼5 times the control level and Fabry CMs were unable to clear exogenous GL-3 as control cells could (Figure 1B). Somewhat surprisingly, all CMs developed strong MLC-2V expression, indicating a universal ventricular-like identity (Figures 1C–1E). As further evidence of authentic CM differentiation, M lines (marked by myomesin-3) were frequently present alternating with Z bands (marked by α-actinin) when imaged on micro-patterned lines (Figure 1D), with this myomesin-3 labeling pattern evident in >80% of control CMs and >90% of Fabry CMs (Figure 1E). CM hypertrophy, a late cardiac phenotype in Fabry patients, was not evident in Fabry CMs at day 45 by 2D single-cell area (Figure 1F) or capacitance (Figure S1).

To gain insight into the functional consequences of *GLA* mutations and the propensity for increased conduction and arrhythmias in patients, electrophysiological and calcium-handling measurements were performed on spontaneously active CMs. Results are shown in Figure 2 and Tables S1 and S2. Fabry CMs displayed evidence of increased excitability, with an increased upstroke velocity (dV/dT max) and a slightly higher spontaneous action potential (AP) frequency coupled to a shorter AP duration (Figures 2A–2C). The maximum diastolic potential (MDP) was higher. Importantly, the peak sodium current density (I_{Na}) was also increased in Fabry CMs; a frequency- and MDP-independent parameter. Consistent with the AP data, calcium transients in Fabry CMs had increased amplitude (ΔF/F), increased rising slope (30%–70%), and decreased peak width duration (corrected for frequency) (Figures 2D and 2E; Table S2). An increased sarcoplasmic reticulum (SR) capacity in Fabry CMs was indicated by the greater caffeine-induced amplitude.

Overall, these data demonstrate that our panel of control and Fabry iPSC lines generate CMs with comparably well-developed structural properties, but Fabry CMs lack α-gal A activity, accumulate GL-3, and develop an increased electrical excitability and a gain of function in calcium handling. This *in vitro* model is therefore a powerful platform for identifying early FD-related biomarkers driven by the primary dysfunction in these cells.

Identification of Fabry Disease Protein Biomarkers in CMs including LIMP-2

To perform a global unbiased biomarker discovery screen, purified CMs (day 35) from our panel of iPSCs were used for liquid chromatography-mass spectrometry (Figure 3A). More than 4,400 proteins were detected (≥1 unique peptide each; false discovery rate <1%) in a screen of three control lines in duplicate and the two Fabry lines in triplicate, with individual protein levels measured over ∼5 orders of magnitude (Figure 3B; complete data in Table S3). As confirmation of the accuracy of CM differentiation and cell purity across the tested samples, five of the six most highly abundant proteins (by average across all samples) were cardiac sarcomere components: myosin heavy chain 6 (MYH6), titin (TTN), cardiac actin (ACTC1), myosin MYH7, and α-actinin (ACTN2) (Figure 3B). Moreover, the levels of these and other sarcomeric proteins were not significantly different between control and Fabry samples (Figures S2A and S2B). To assess differential expression, we first performed an analysis with limma (Ritchie et al., 2015) using a control versus Fabry model. At a cutoff of ≥0.5 log2 fold-change and adjusted p value <0.1 (Benjamini-Hochberg) we identified nine differentially expressed proteins (Figure 3C). Compared with controls, lysosomal-specific proteins including lysosomal membrane protein 2 (SCARB2/LIMP-2), glucocerebrosidase (GBA), and galactocerebrosidase (GALC) were upregulated in Fabry CMs; also upregulated were heat shock-related 70 kDa protein 2 (HSPA2/HSP70-2), GMP reductase 1 (GMPR), and DNA-methyltransferase 3A (DNMT3A). Fumarylacetoacetate hydrolase domain-containing protein

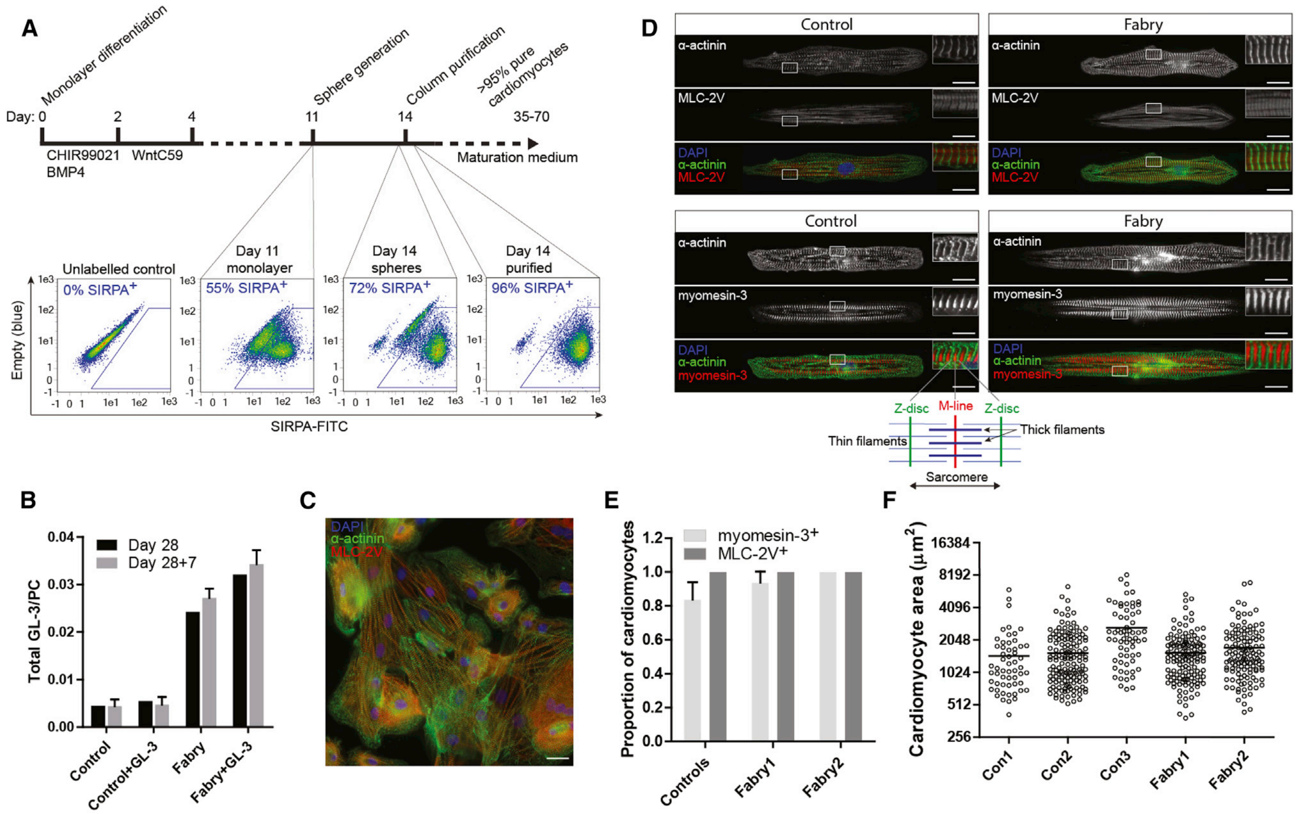

Figure 1. CM Generation from Control and Fabry iPSCs, and Their Structural Characterization

(A) Cardiomyocyte differentiation and purification protocol, with FACS plots of SIRPA immunostaining (typical examples) in a differentiated population at day 11, after sphere generation, and again after column purification at day 14.

(B) Levels of GL-3, normalized to phosphatidylcholine (PC) content, in control and Fabry CMs at day 28 cultured ±1 μM exogenous GL-3, and in the same populations 7 days later with the exogenous GL-3 removed (day 28 + 7). Bars represent mean ± SD from two independent experiments/differentiations.

(C) Typical immunostaining of α-actinin and MLC-2V in a purified population of iPSC-CMs returned to monolayer culture.

(D) Typical immunostaining of α-actinin (Z-disc), MLC-2V, and myomesin-3 (M-line) of single control and Fabry1 CMs aligned on micropatterned glass. The schematic below highlights the sarcomeric structural organization evident from this labeling.

(E) The total proportion of single cells expressing myomesin-3 or MLC-2V in an organized pattern. Note that all measured cells expressed MLC-2V. Bars represent mean ± SD from three independent experiments/differentiations (>30 cells quantified per experiment).

(F) Single CM 2D area measured using fixed immunolabelled cells. The horizontal line signifies the mean. Data were collected over three independent experiments/differentiations. Scale bar, 20 μm.

See also Figure S1.

2A (FADH2A), low-density lipoprotein receptor, and mitochondrial endonuclease G (ENDOG) were among the downregulated proteins. ENDOG was previously shown to regulate mitochondrial function, lipid metabolism, and hypertrophy in CMs (McDermott-Roe et al., 2011). Loading with the cationic fluorescent dye TMRM indicated that Fabry CMs may indeed have a lower mitochondrial membrane potential than controls (Figure S2C), which could indicate a mitochondrial functional deficiency, although mtDNA-encoded proteins were not significantly decreased at day 35 (Figure S2D). The enrichment of lysosomal-associated proteins in this list is significant (Fisher's exact test, $p < 0.01$) when considering that only 179 of these 4,464 proteins have been associated with lysosomes in the literature (Brozzi et al., 2013). Interestingly, LIMP-2 is the lysosomal trafficking receptor for GBA, but published data suggest that it may have additional roles in the heart and in lysosomal biogenesis, making it a particularly interesting functional candidate (Reczek et al., 2007; Schroen et al., 2007).

To increase the power of this approach, and overcome limitations in parallel sample processing by mass spectrometry, we examined internal ratio differences in protein levels against the control mean so that the number of

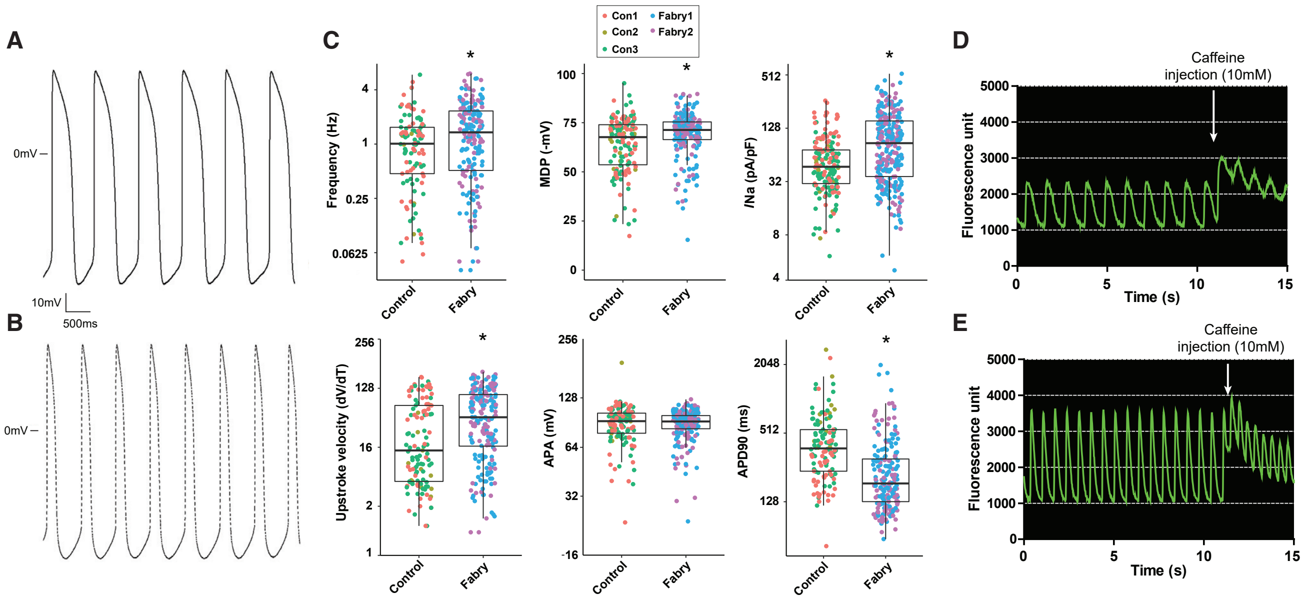

Figure 2. Electrophysiology and Calcium-Handling Measurements in Control and Fabry CMs

(A and B) Typical action potential (AP) traces in control (A) and Fabry CMs (B).

(C) Quantification of electrophysiological parameters in spontaneously active cells. Points represent individual cells from three control lines and two Fabry lines. Boxes indicate the median ± quartiles and whiskers show the range minus outliers. MDP, maximum diastolic potential; I_{Na}, peak sodium current density; APA, action potential amplitude; and APD90, action potential duration at 90% repolarization. Statistical significance was calculated using an unpaired t test, *p < 0.05.

(D and E) Typical calcium transients (Cal-520 fluorescence) and response to caffeine injection (where indicated) in control (D) and Fabry CMs (E). Data were collected over four independent experiments/differentiations for electrophysiology and two for calcium imaging.

See also Figure S1.

replicates could be increased. Data from a smaller-scale mass spectrometry run were added (Table S3), giving five independent replicates per group in total. A total of 4,915 proteins were detected in the second run, giving a combined 5,178 proteins in total, 4,201 proteins being common to both runs. This list of 4,201 proteins was filtered for proteins with ≥0.5 log2 fold-change for both Fabry lines individually against controls and an adjusted p value <0.05 (one-sample t test; $\mu = 0$), which identified 53 up- and 19 downregulated proteins (Table S4). Seven of the nine previously identified candidates also qualified by these criteria (SCARB2/LIMP-2, GBA, GALC, HSPA2/HSP70-2, DNMT3A, FADH2A, and ENDOG), further enhancing their validity as Fabry biomarkers. Figure 3D shows the proteins with ≥2 fold-change in a heatmap. A decrease in GLA/α-gal A was revealed in this list, peptides for which were almost completely absent in Fabry CMs as expected. Genome ontology showed that the "glycosylceramide catabolic process" was the most significantly enriched biological process, and that "lysosomal lumen" the most significant cellular component from this list of 72 proteins (Tables S5 and S6). However, many other non-lysosomal proteins also emerged, including three glutathione S-transferases and three additional heat shock proteins (HSPs), which were all increased in Fabry CMs (GSTM1, 7.9-fold; GSTM4, 1.5-fold; GSTT2B, 5.1-fold; HSPB2, 1.4-fold; HSPB3, 1.7-fold; and HSPA4L, 2.1-fold). The SR calcium-buffering protein, calsequestrin 2 (CASQ2), and the mitochondrial fatty acid transporter, carnitine O-palmitoyltransferase 1 (CPT1B), were also increased by 3.0- and 2.4-fold, respectively. Protein-protein interaction (PPI) network analysis using STRING identified 34 edges (putative PPIs) between these 72 differentially expressed proteins (PPI enrichment p value <0.01) (Figure 3F).

To validate a subset of these potential Fabry biomarkers with interesting roles in lysosomes, heat shock response, and calcium handling, western blots were performed using independent cell lysates from CMs at 35–70 days of differentiation, along with normal human heart tissue sample for reference. The later time points were included to assess the impact of continued cell maturation and pathogenesis. An upregulation of SCARB2/LIMP-2, GBA, and HSPA2/HSP70-2 was observed in both Fabry lines beyond 35 days of differentiation (Figures 4A and 4B). LIMP-2 and GBA were not increased in Fabry iPSCs before differentiation (Figures 4C and 4D), whereas HSP70-2 already showed increased abundance, most notably in Fabry1 iPSCs. However, another iPSC clone from this patient

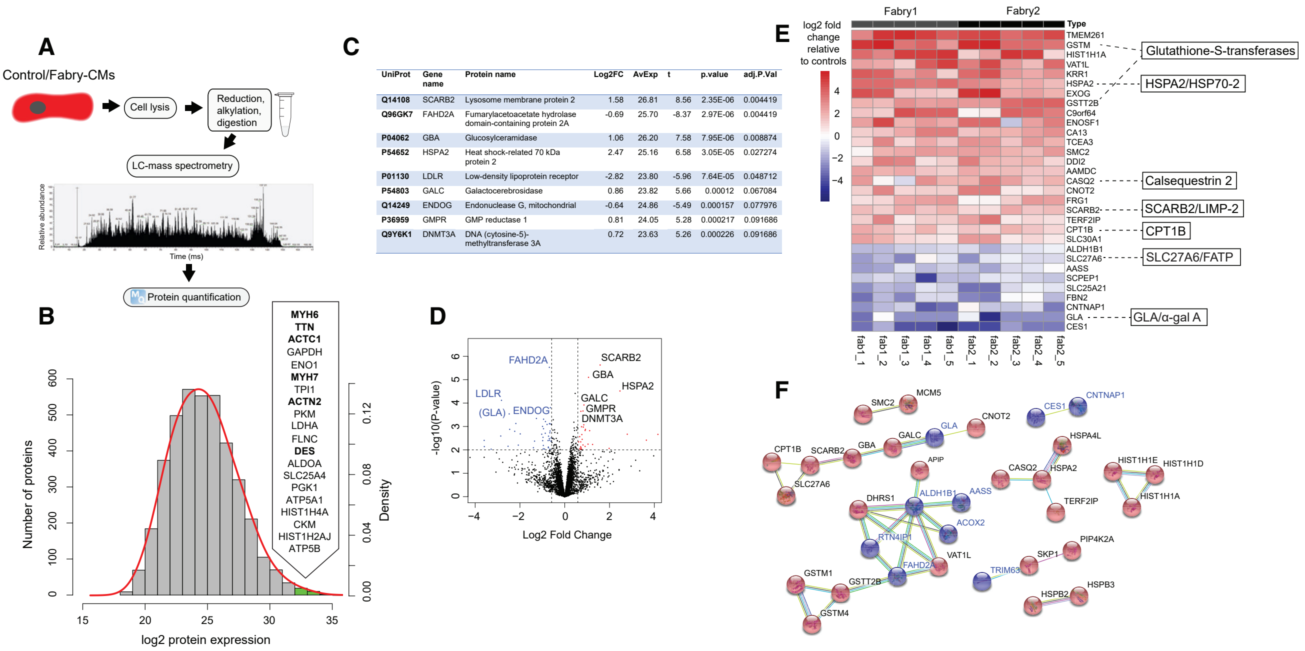

UniProt	Gene name	Protein name	Log2FC	AvExp	t	p.value	adj.P.Val
**Q14108**	SCARB2	Lysosome membrane protein 2	1.58	26.81	8.56	2.35E-06	0.004419
**Q96GK7**	FAHD2A	Fumarylacetoacetate hydrolase domain-containing protein 2A	-0.69	25.70	-8.37	2.97E-06	0.004419
**P04062**	GBA	Glucosylceramidase	1.06	26.20	7.58	7.95E-06	0.008874
**P54652**	HSPA2	Heat shock-related 70 kDa protein 2	2.47	25.16	6.58	3.05E-05	0.027274
**P01130**	LDLR	Low-density lipoprotein receptor	-2.82	23.80	-5.96	7.64E-05	0.048712
**P54803**	GALC	Galactocerebrosidase	0.86	23.82	5.66	0.00012	0.067084
**Q14249**	ENDOG	Endonuclease G, mitochondrial	-0.64	24.86	-5.49	0.000157	0.077976
**P36959**	GMPR	GMP reductase 1	0.81	24.05	5.28	0.000217	0.091686
**Q9Y6K1**	DNMT3A	DNA (cytosine-5)-methyltransferase 3A	0.72	23.63	5.26	0.000226	0.091686

Figure 3. Proteomic Analysis of Control and Fabry CMs

(A) Schematic showing the workflow performed for proteome analysis using liquid chromatography-mass spectrometry.

(B) Histogram showing the distribution of all detected proteins by their average expression level across all samples (control and Fabry). The red line shows the fitted normal density curve. The top 20 most highly expressed proteins (green bars) are listed in order, with sarcomeric proteins highlighted in bold.

(C) "TopTable" output from limma of differentially expressed proteins. Log2FC, log2 fold-change of Fabry samples compared with controls; AvExp, average expression across all samples as log2 value; t, t statistic; adj.P.Val, p value with Benjamini-Hochberg adjustment.

(D) Volcano plot showing the results of the limma analysis with significantly upregulated proteins in black and downregulated proteins highlighted in blue. GLA is also highlighted for reference.

(E) Heatmap showing protein expression relative to controls in five lysates from each Fabry line, from independent differentiations, for proteins $\geq$2-fold changed up or down by average in both lines. Some particularly interesting candidates are highlighted to the right.

(F) Results of STRING analysis showing potential protein-protein interactions, as run on the 72 differentially expressed proteins in Table S4. Up- and downregulated proteins in Fabry CMs are shown in red and blue, respectively.

See also Figure S2.

with lower baseline HSP70-2 levels (Figure S3A) still showed an increase relative to controls after differentiation (Figures 4E and S3B). Elevated levels of LIMP-2 and GBA in Fabry CMs were not explained by increased lysosomal biogenesis, as LAMP-2 was not increased and lysosomes were not more numerous by immunocytochemistry (Figures 4A, 4B, and 4F). CASQ2 expression was clearly maturation dependent, but when detectable at later time points was increased in Fabry CMs, consistent with the enhanced calcium handling of Fabry CMs shown above. Overall, our western blot data strongly support the quantitative accuracy of the proteomics data.

Secretome Analysis Identifies Secreted Fabry Biomarkers including Cathepsin F

To identify biomarkers differentially secreted by Fabry CMs, mass spectrometry was performed on proteins isolated from CM-conditioned medium (Figure 5A). A total of seven samples from each Fabry line were compared with samples from four control lines across three mass spectrometry runs; each sample from an independent differentiation and experiment. After subtraction of potential matrix-derived (Matrigel) contaminants, a total of 3,013 proteins were identified ($\geq$1 unique peptide each; false discovery rate <1%), which spanned ~5 orders of magnitude in abundance like the cell proteome (Figure 5B; complete data in Table S3). A list of the top 20 most highly abundant proteins across all control and Fabry secretomes is shown in Figure 5B, and was made up largely of extracellular matrix and associated proteins. Proteins were searched by databases for known plasma proteins (Nanjappa et al., 2014), and proteins with a signal peptide destined toward the secretory pathway (Petersen et al., 2011), using the Human Protein Atlas (Uhlén et al., 2015). The total secretome was enriched for plasma proteins (50% versus 35% in the proteome) and proteins with signal peptides (24% versus 9% in

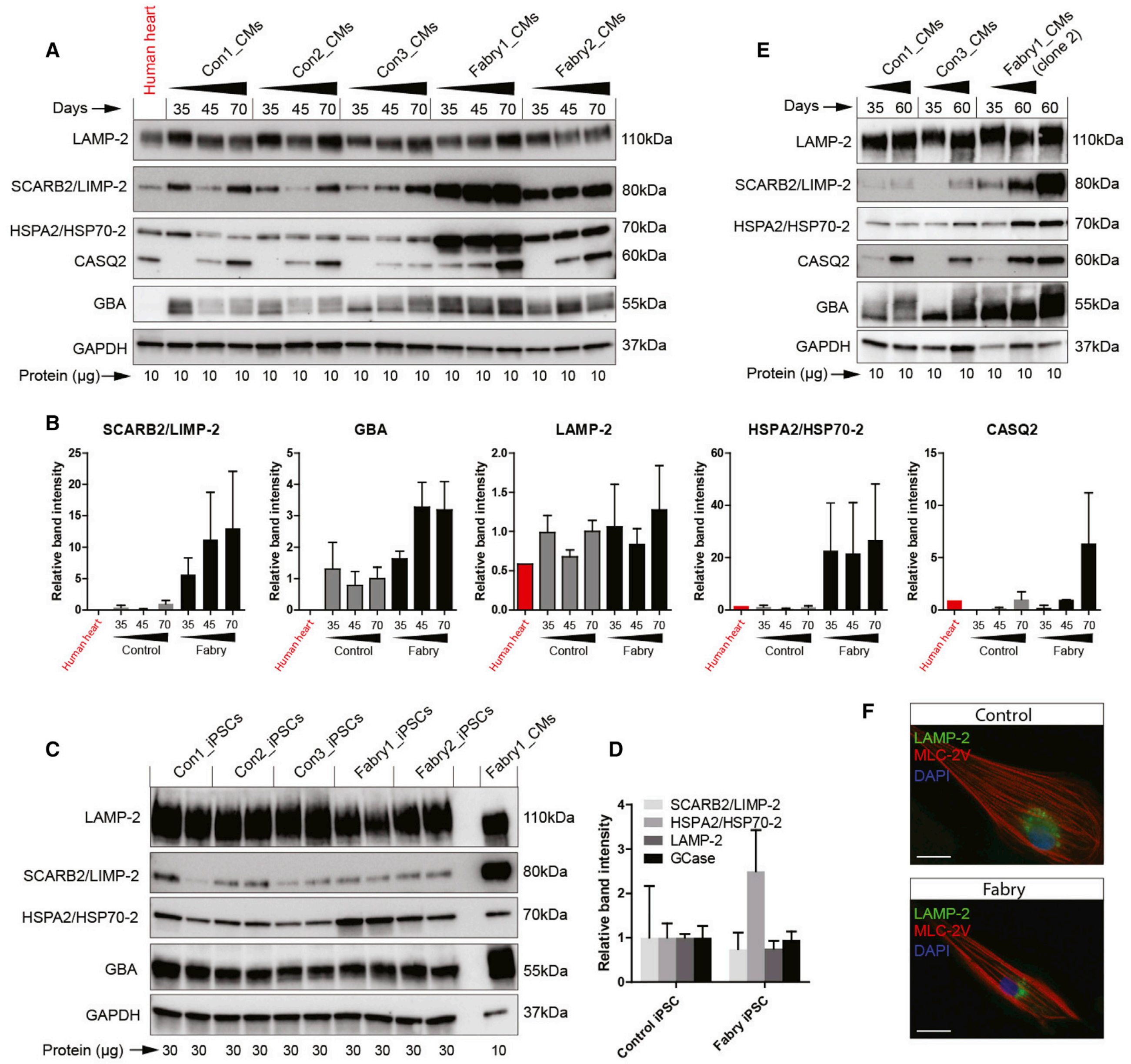

Figure 4. Validation of Candidate Fabry Protein Biomarkers by Western Blot

(A) Western blots of control and Fabry CMs taken at three time points from three differentiations. Protein from a human heart lysate was loaded in the first lane.

(B) Quantification of western blots in (A) grouped as control and Fabry, calculated as relative band density normalized to GAPDH levels.

(C) Western blots of undifferentiated control and Fabry iPSCs with independent duplicates. A Fabry CM population is shown in the far right lane as reference.

(D) Quantification of the iPSC western blots in (C), calculated as relative band density normalized to GAPDH levels.

(E) Western blots showing the levels of the candidate Fabry biomarkers in CMs derived from a second clone of Fabry1 iPSCs at two time points of differentiation.

(F) Immunocytochemistry showing typical LAMP-2 and MLC-2V staining in control and Fabry CMs. Bar data indicate the mean ± SD. Scale bar, 20 µm.

See also Figure S3.

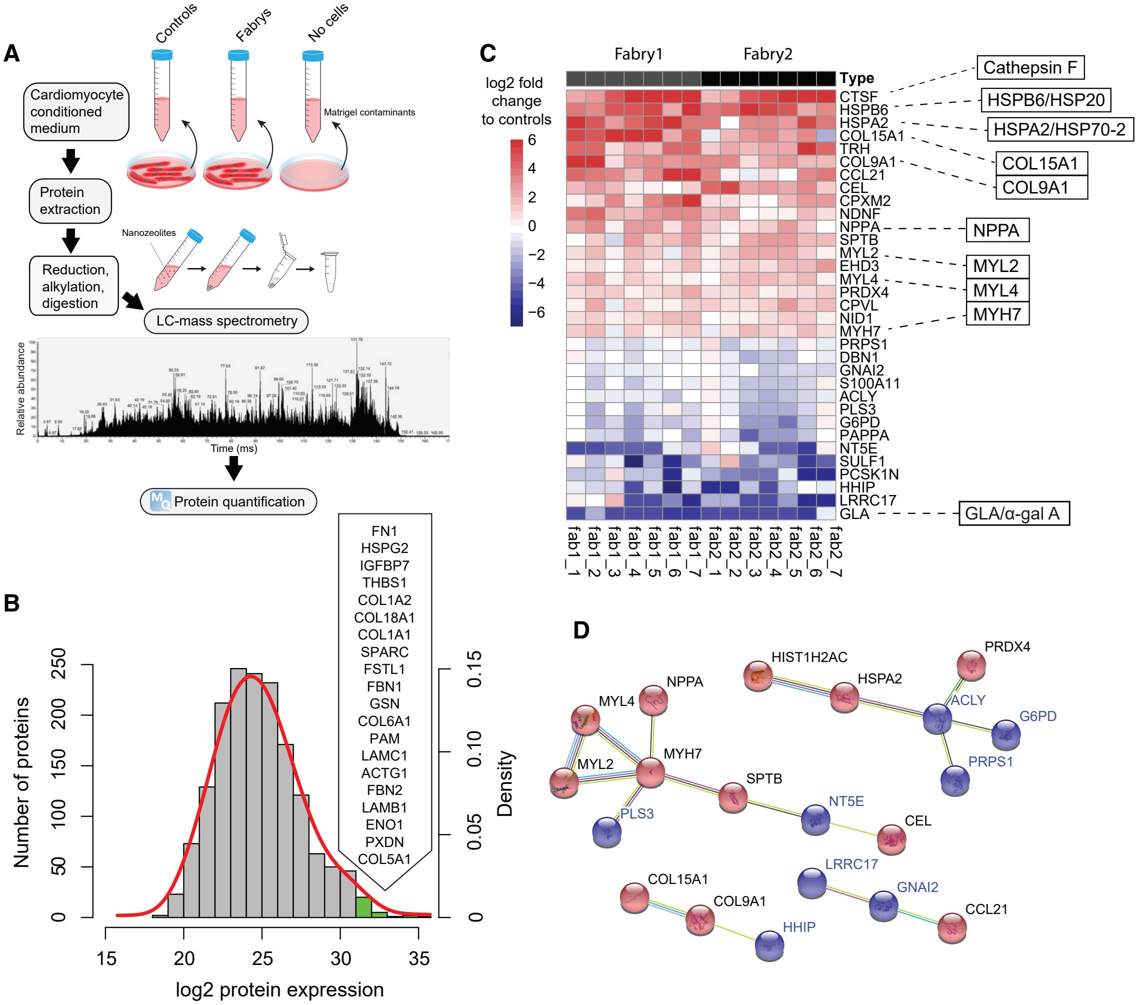

Figure 5. Secretome Analysis of Control and Fabry CMs

(A) Schematic showing the workflow for protein isolation and secretome analysis using liquid chromatography-mass spectrometry from CM-conditioned medium.

(B) Histogram showing the distribution of all detected proteins by their average expression level across all samples (control and Fabry). The red line shows the fitted normal density curve. The top 20 most highly expressed proteins (green bars) are listed in order.

(C) Heatmap showing protein expression relative to controls in seven lysates from each Fabry line, from independent differentiations, of proteins >1 log2 fold changed up or down in both lines by average. Some particularly interesting candidates are highlighted to the right.

(D) Results of STRING analysis showing potential protein-protein interactions, as run on the 34 differentially expressed proteins in Table S7. Up- and downregulated proteins in Fabry CMs are shown in red and blue, respectively.

See also Figure S4.

the proteome). A total of 718 proteins were uniquely identified in the secretome, 58% of which had a signal peptide. Enrichment was also seen for known cardiac secreted proteins such as atrial natriuretic peptide (NPPA) and natriuretic peptide B, which were both at levels above the 90th percentile in the secretome but barely detectable in the cell proteome. Together, these observations provide high confidence that this is a bona fide CM secretome.

To identify differentially abundant proteins between control and Fabry secretomes, the same multi-experiment filtering approach was performed as for the proteome: ≥0.5 log2 fold-change for both Fabry lines individually against controls and an adjusted p value <0.05 (one-sample t test; $\mu = 0$). This identified 20 up- and 14 downregulated proteins (Table S7). Figure 5C shows the proteins with ≥2 fold-change in a heatmap. Importantly, the most downregulated protein in Fabry CM secretomes was GLA/α-gal A, which interestingly was more easily detectable in control secretomes than in the cell proteomes. The most highly overrepresented protein in Fabry

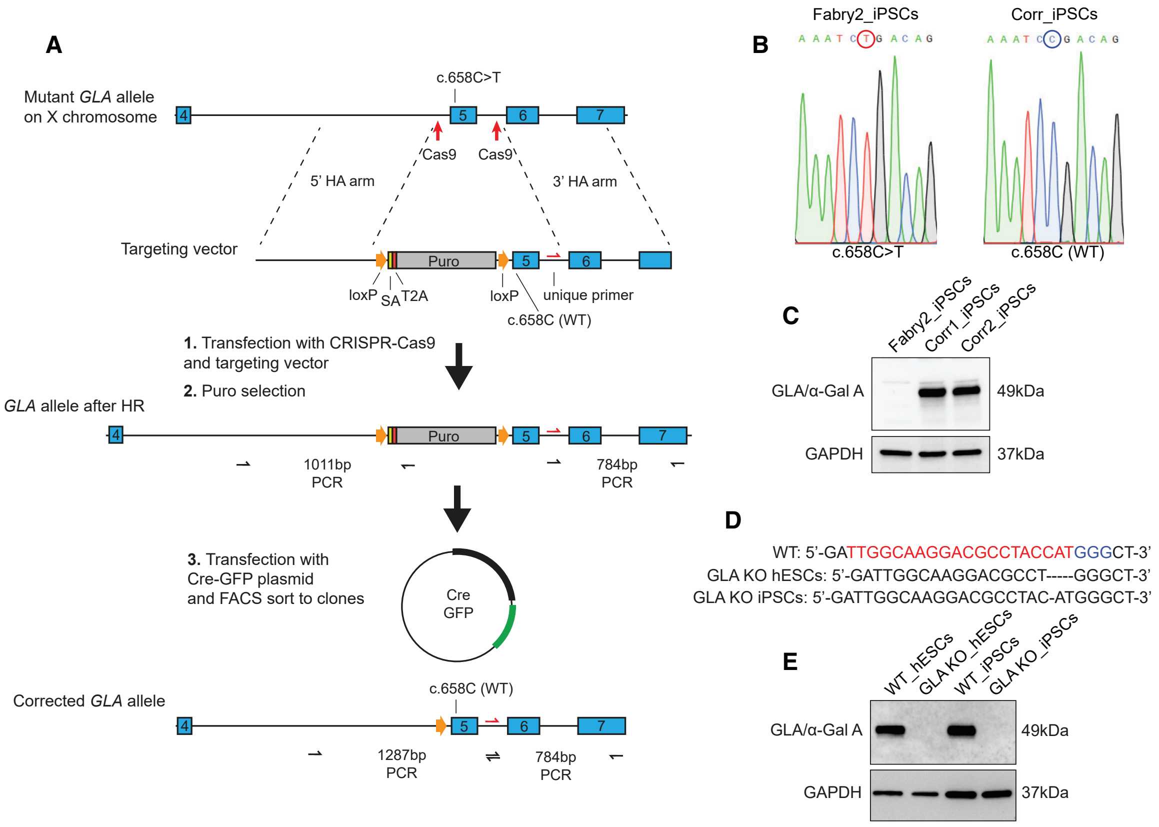

Figure 6. Genetic Correction of *GLA* Point Mutation and Generation of *GLA* Knockouts

(A) Schematic showing the strategy used to correct the *GLA* c.658C > T mutation and the three main steps: CRISPR-mediated knockin, selection, and removal of selection. Successful homologous recombination and Cre-mediated excision of the selection cassette could be confirmed using the PCR amplicons shown.

(B) Sequence chromatograms across nucleotide c.658 showing successful correction of the mutation.

(C) Western blot showing restoration of α-gal A expression in two sequence-corrected clones.

(D) In-del mutations in *GLA* introduced into wild-type hESCs and iPSCs for gene knockout. The red and blue nucleotides signify the crRNA and PAM sequences, respectively.

(E) Western blot showing complete knockout of α-gal A expression as a result of the mutations in (D).

CM secretomes was the lysosomal cysteine protease cathepsin F (CTSF), which was increased by 30-fold. Also highly overrepresented was the heart-enriched and cardioprotective HSPs, HSPB6/HSP20 (22-fold) (Fan and Kranias, 2011) and HSP70-2 (18-fold). HSP70-2 is therefore not only elevated in Fabry CMs but also secreted at higher levels. Other notable proteins that were increased included the known heart disease biomarker NPPA (2.7-fold); three cardiac myosins (MYL2, MYL4, and MYH7); and two collagen molecules (COL15A1 and COL9A1). PPI network analysis of the differentially expressed secretome proteins identified several putative interactions (Figure 5D), and combining this with the cell proteome list generated a large network with 82 edges (PPI enrichment $p < 0.01$), with ATP citrate synthase (ACLY) forming a putative hub node (Figure S4). ACLY is responsible for production of extramitochondrial acetyl-coA and has important roles in nutrient catabolism and lipid biosynthesis (Pinkosky et al., 2017).

GLA Mutation Correction and Gene Knockout Support the Validity of Fabry Biomakers

To assess the impact of α-gal A deficiency in CMs more directly, we took two strategies. Firstly, to correct the *GLA* point mutation in one of the Fabry iPSC lines, and secondly to generate in-del mutations in *GLA* in wild-type hESC/iPSC lines to create gene knockouts. To correct the c.658C > T mutation in Fabry2 iPSCs we used a selection strategy (Figure 6A). Because *GLA* is expressed in hPSCs we incorporated a splice acceptor and puromycin resistance gene, together with wild-type exon 5, into the *GLA* gene, so that successfully targeted cells could be selected. After Cre-mediated excision of the selection cassette and cell cloning, full-length wild-type *GLA* expression was restored

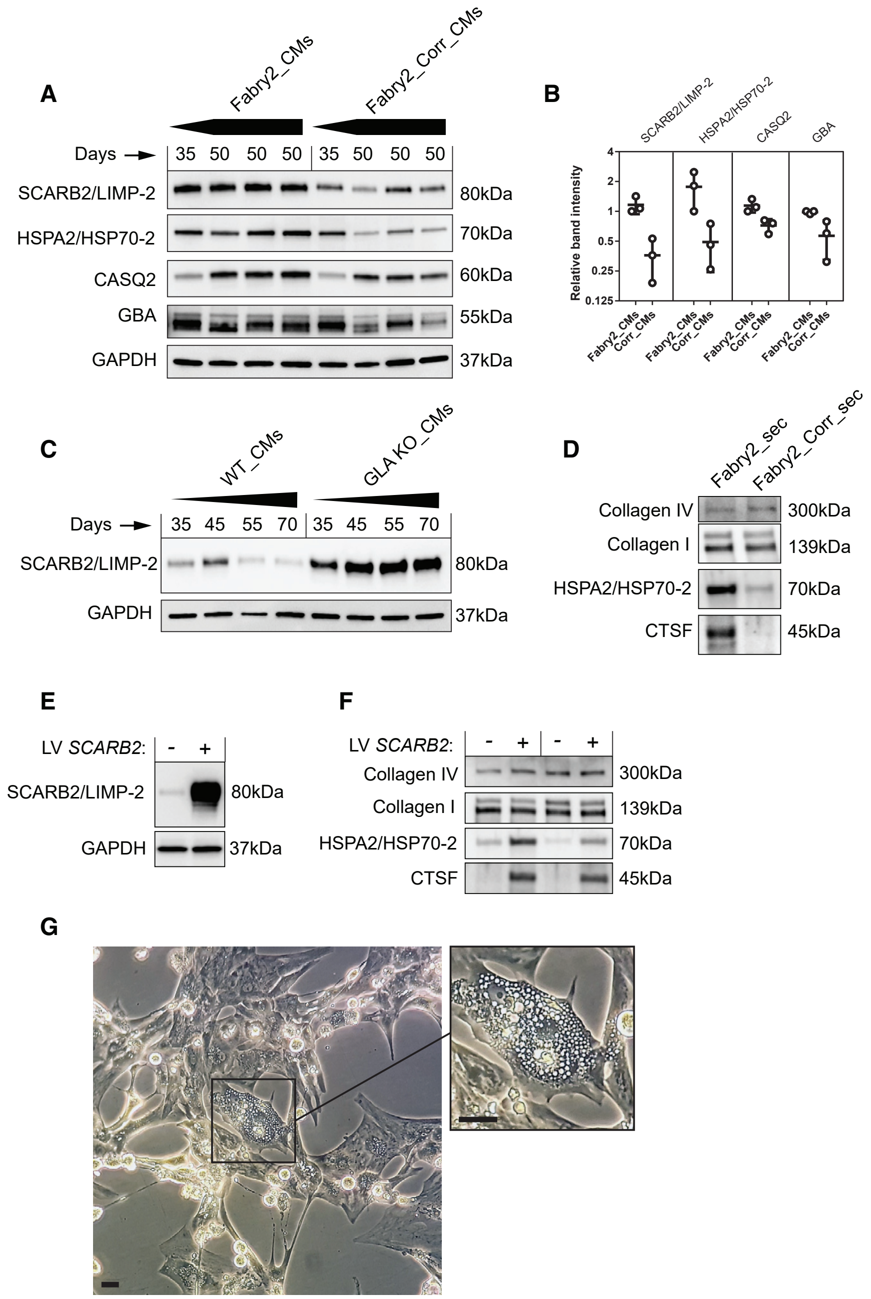

Figure 7. Validation of Biomarkers Using Isogenic Controls and Impact of LIMP-2 Accumulation

(A) Western blots of Fabry and gene-corrected CMs taken at two points from four independent differentiations.

(B) Quantification of western blots in (A). Error bars show SD.

(C) Western blot of wild-type CMs derived from hESCs and *GLA* knockouts taken at different points from four independent differentiations.

(D) Western blot of secretome samples from Fabry- and corrected CM-conditioned medium.

(E) Western blot showing overexpression of LIMP-2 in wild-type CMs by lentivirus.

(F) Western blot of secretome samples from two independent experiments showing the impact of LIMP-2 overexpression on protein secretion.

(G) Image of purified CMs overexpressing LIMP-2 by lentivirus. The high magnification image highlights the ubiquitous vacuoles in one particular cell. Scale bar, 100 μm.

See also Figure S5.

in all analyzed clones (Figure 6B). Two were expanded and the restoration of α-gal A protein expression was confirmed (Figure 6C). *GLA*/α-gal A knockout clones were successfully derived from MAN13 hESCs (c.121_125delTACCA) and con3 iPSCs (c.123delC) (Figures 6D and 6E).

Following cardiac differentiation of these isogenic lines, the four protein biomarkers previously validated by western blot were assessed over time. Correction of the *GLA* c.658C > T mutation diminished the levels of all these markers when quantified at day 50 (Figures 7A and 7B). In hESC-CMs, *GLA*/α-gal A knockout cells showed a similar marker profile to the Fabry CMs (Figures S5A and S5B), with LIMP-2 accumulation being the most dramatic change (Figure 7C). LIMP-2 accumulation was further confirmed in iPSC-CMs with *GLA* knockout (Figure S5C), suggesting that this is a robust response to α-gal A deficiency in CMs. *SCARB2* mRNA levels were unchanged, demonstrating that accumulation is not due to increased gene expression (Figure S5D). Secretion of CTSF and HSP70-2 were assessed by western blotting and were both found to be markedly decreased after gene correction (Figure 7D).

LIMP-2 Accumulation Drives the Secretion of Cathepsin F and HSP70-2

As LIMP-2 is known to have an important role in endolysosome biogenesis, we investigated whether LIMP-2 accumulation alone could drive the secretion of these proteins. Measurements after LIMP-2 overexpression in wild-type CMs or 293FT cells confirmed this hypothesis (Figures 7E, 7F, and S5). Collagen I and IV levels were unaffected and no increased cell death was observed. Light microscopy revealed an increase in vacuole formation in LIMP-2 overexpressing CMs, a feature of late-stage Fabry cells (Figure 7G and Video S2) (Chimenti et al., 2008), although these were not observed in the unmanipulated Fabry CMs within the studied time frame.

DISCUSSION

Many disease phenotypes are cell-type specific and must be investigated in the appropriate cellular context. Here, we used patient-derived iPSCs to study the consequences of *GLA* mutations in CMs as a model of FD. Although Fabry CMs were comparable with controls in their size and structural maturity, developing uniform MLC-2V expression and sarcomere M lines, they accumulated GL-3 and showed evidence of increased electrophysiological excitability. Moreover, a proteomics-based screen identified FD-specific changes in specific proteins involved in lysosomal function and heat shock response, presenting potential new disease biomarkers. The accumulation of LIMP-2 was the most significant difference and was found to directly promote the secretion of the lysosomal protease CTSF and HSP70-2, suggesting that α-gal A deficiency causes major changes to endolysosomal pathways. Our data support the concept that this hPSC model can reveal important early molecular changes in FD.

Heart disease is the main cause of death in patients with FD, with the accumulation of lysosomal GL-3 in the heart frequently resulting in the progressive development of hypertrophy and arrhythmia. Increased conduction velocity in atrial and ventricular CMs has been proposed as one possible cause of arrhythmic risk in FD (Namdar, 2016). Ion handling in endothelial cells and neurons has been reported to be affected by GL-3 accumulation (Choi et al., 2014, 2015; Park et al., 2011); however, data on Fabry CMs were lacking. Peak sodium current density is an important determinant of myocardial conduction velocity (King et al., 2013). Here, we show that Fabry CMs display an enhanced I_{Na} relative to controls, and an increased dV/dT max, suggesting enhanced sodium channel function, as well as displaying a higher spontaneous AP frequency and a shorter AP duration. Enhanced function was equally observed at the level of calcium handling. Imbalanced glycosphingolipid levels may therefore lead to alterations in ion channel expression, or more likely to changes in their trafficking or turnover, resulting in direct effects on CM electrophysiology, with potential implications for arrhythmia.

Human PSC technology makes it possible to study heart diseases in the appropriate cells at a genome- and proteome-wide level. However, in contrast to transcriptomic data on PSC CMs, which are now ubiquitous, there has been limited published proteome-level data on purified populations of these cells. The composition of the CM secretome is even less well defined, even though cardiac-derived secreted proteins, or cardiomyokines, may have important roles in cell-cell communication in heart disease (Doroudgar and Glembotski, 2011), and are of special interest concerning the potential paracrine therapeutic effects of human PSC CMs after transplantation into the diseased heart (Tachibana et al., 2017). In this study, using well-characterized and carefully purified iPSC-CMs, we report the quantification by mass spectrometry of >5,000 cellular proteins, and a further ∼700 proteins uniquely identified in their secretomes. These datasets will be valuable resources for the research community and benchmarks for future studies.

Several of the proteins that we found to be differentially expressed/abundant in Fabry CMs or in the extracellular space have known roles in other LSDs (including Gaucher disease, SCARB2/LIMP-2 and GBA; Krabbe disease, GALC; and ceroid lipofuscinosis, CTSF), but had never been implicated in FD. Our data also implicate HSPs in the cellular response to α-gal A deficiency in CMs, specifically family members HSPB2, HSPB3, HSPA4L, HSPB6/HSP20, and HPS70-2, which may have cardioprotective roles. HSPs are a group of chaperones important for maintaining normal cell metabolism and function under stress. HSP70-2, a member of the highly conserved HSP70 family (Daugaard et al., 2007), was increased in Fabry CMs, and was also secreted at higher levels. Interestingly, HSP70 treatment has been shown to reverse lysosomal pathology in models of LSDs including the murine model of FD ($Gla^{-/-}$) (Kirkegaard et al., 2016). Whereas HSP70-2 has been suggested to lack the lysosome stabilization activity of HSP70, recombinant HSP70-2 was also reported to be similarly taken up into the lysosomal lumen (Kirkegaard et al., 2010). Also increased in Fabry secretomes was HSPB6/HSP20, which is a cardioprotective and secreted HSP known to be upregulated in CMs by stress (Chu et al., 2004; Martin et al., 2014; Zhang et al., 2012). Overall, our data support a possible role for these HSPs in an adaptive response to α-gal A deficiency and as potential new biomarkers in FD.

In addition to specific HSPs, proteomics analysis of the Fabry CM secretome identified CTSF as being highly enriched. The secretion of cathepsins has been documented

previously, and these proteins may have important extracellular roles (Fonovic and Turk, 2014; Öörni et al., 2004). Enhanced secretion may be due to impaired lysosomal targeting, lysosomal leakage, or increased active secretion in Fabry CMs. Of relevance, certain cathepsins have been shown to be upregulated in plasma of patients with the LSD Gaucher disease (Moran et al., 2000). Although mechanisms for the active secretion of CTSF, HSPB6, and HSP70-2 have not yet been specifically defined, extensive work has been published describing the active secretion of HSP70 in response to heat shock, which may be of important relevance (Mambula and Calderwood, 2006). Specifically, HSP70 release has been shown to involve transit through an endolysosomal compartment containing cathepsin D, and can be inhibited by lysosomotropic agents, similar to interleukin-1β (Andrei et al., 1999). Enhanced exocytosis of HSP- and CTSF-containing vesicles in Fabry CMs is therefore an interesting possibility.

LIMP-2 was the most significant biomarker in our proteome analysis of Fabry CMs, and was found to accumulate through a post-transcriptional mechanism. LIMP-2 is the lysosomal trafficking receptor for GBA; however, LIMP-2 has also been shown at intercalated disc structures in the heart, and its expression in CMs found to correlate with mechanical stress and to play a crucial role in CM hypertrophic response (Schroen et al., 2007). Of further relevance to FD, LIMP-2 has been shown to be a powerful regulator of endosome and lysosome biogenesis and capable to inducing the formation of intracellular vacuoles (Kuronita et al., 2002). Similar structures are widely reported in Fabry cells *in situ*, although typically enclosing concentric lamellar structures (Pieroni et al., 2003). Although the Fabry CMs in this study did not clearly developed such vacuoles, other publications have noted these *in vitro* (Itier et al., 2014; Kuramoto et al., 2018). Nevertheless, overexpression of LIMP-2 clearly induced this phenotype.

With an apparent role in endolysosomal organization, we tested whether LIMP-2 accumulation might be a primary mediator of HSP70-2 and CTSF secretion. Our data support this idea, as secretion of both proteins could be enhanced by overexpressing LIMP-2 in wild-type CMs. Whether this has an adaptive role in FD in potentially aiding the clearance of GL-3, or has pathological consequences, will be important to investigate. In CMs, any changes in trafficking or vesicle secretion/exocytosis could have significant consequences for ion channel density at the plasma membrane, and therefore CM electrophysiology. Recent data suggesting that exosome production might be increased in *GLA* knockout CMs also merit further study in this context (Song et al., 2019).

In the observed experimental time frame of the current study, CM hypertrophy was not observed. This is contrary to what has been reported by some (Chien et al., 2016; Chou et al., 2017), but not all studies investigating FD iPSC-derived CMs (Kuramoto et al., 2018). By contrast, the accumulation of GL-3 has been universally reported. It will therefore be important to investigate by what mechanism this primary dysfunction can lead to hypertrophy, and whether LIMP-2, which has shown to be essential for adaptive hypertrophy in a rat model of hypertension, plays a role in this (Schroen et al., 2007). The isogenic lines and genetic tools presented here will facilitate these future investigations.

In summary, our study has shown the power of the iPSC model to reveal early functional changes and the development of a distinctive biomarker expression profile in FD CMs. These biomarkers may be of utility in drug screening and in elucidating the earliest pathological events and cascades in FD cells. Quantification in patient plasma and urine samples will be an important next step toward validating their relevance in patients. Heterogeneity is a major feature of FD, and personalized risk stratification for arrhythmias in patients, for example, through biomarker profiling, might help cardiologists judge the need for cardioverter defibrillator implantation. Inclusion of patients with FD in large-scale sequencing efforts, such as the UK's 100,000 Genomes Project (Genomics England, 2017), will provide further opportunity for stratification by enabling the study of genetic variation in the putative genes and pathways implicated in the heart, correlated to clinical status. The wealth of new data reported here may have implications beyond FD, given that some of the principal candidates, including LIMP-2, are suggested to be involved in other heart diseases and may therefore represent common players acting in CM pathology. A better understanding of these mechanisms will no doubt accelerate the development of more effective and increasingly personalized therapies for patients.

EXPERIMENTAL PROCEDURES

See Supplemental Information for full experimental procedures.

hPSC Culture and Cardiac Differentiation

The derivation of iPSC lines from two male patients with FD carrying non-sense mutations in *GLA* was described previously (Itier et al., 2014). The patient fibroblasts were obtained from the Coriell Institute for Medical Research. In this article, clone GM00107 (c.485G > A; W162X) is referred to as Fabry1 and clone GM00881 (c.658C > T; R220X) as Fabry2. Four healthy male control iPSC lines were also derived internally from skin fibroblasts using the same protocol. MAN13 hESCs were kindly provided by the Kimber lab (Ye et al., 2017). MAN13 hESCs are a UK Stem Cell Bank registered line and this study was performed in full compliance with the laws, regulations, and procedures that govern hESC use in the UK. The study conducted is in full compliance with Sanofi guidelines on the use of hESCs. All experiments

involving hESCs were performed at The University of Manchester. hPSCs were maintained in mTeSR medium (Stem Cell Technologies). Cardiac differentiation was induced as follows: 10 ng/mL BMP4 + 4 μM CHIR991021 (Miltenyi Biotec) for 48 h and then 2 μM Wnt C-59 (Selleckchem) for 48 h. At day 11, cells were transferred to suspension for 3 days. For suspension culture and plating, cells were maintained in CM medium. See Supplemental Information. CMs were purified using a MACS column-based PSC-Derived Cardiomyocyte Isolation Kit (Miltenyi, no. 130-110-188) and replated on Matrigel-coated plastic. After 2 days the medium was changed to a maturation-promoting formula containing 5 nM T3 + 5 nM dexamethasone + 20 μM palmitic acid (conjugated to fatty acid-free BSA) + 0.25% FBS, as supported by our previous work (Birket et al., 2015). CMs were analyzed between days 35 and 70 of differentiation.

CRISPR-Cas9 Gene Editing

The *GLA* c.658C > T mutation was corrected by delivering ribonucleoprotein (RNP) complexes of CRISPR-Cas9 targeting sequences flanking exon 5, along with a targeting vector into Fabry2 iPSCs by nucleofection. RNP complexes were made by complexing cr:tracRNA duplex (IDT) with S.p. Cas9 Nuclease V3 (IDT). The targeting vector incorporated a 5′ homology arm of 801 bp and a 3′ homology arm of 750 bp flanking corrected exon 5 (pUC57-5′H-loxP-SA-2A-Puro-loxP-correctExon-3′H). After 48 h of electroporation, iPSCs were selected with puromycin for 48 h, then electroporated with a Cre expression vector (pCAG-Cre-IRES2-GFP; Addgene plasmid no. 26646), FACS sorted for GFP expression, and directly cloned. Expanded clones were screened by PCR, taking advantage of a unique intronic primer site introduced 3′ of exon 5, and sequenced. All analyzed clones were correctly targeted.

To generate *GLA* knockout hPSC lines, MAN13 hESCs and con 3 iPSCs were electroporated as above using a crRNA sequence targeting exon 1 (5′ TTGGCAAGGACGCCTACCAT 3′), which was found to induce in-del mutations with 60%–80% efficiency. Cells were cloned and screened by sequencing. Clones with frameshift mutations and undetectable α-gal A expression by western blot were used for subsequent experiments: MAN13 hESCs (*GLA* c.121_125delTACCA) and con3_iPSCs (*GLA* c. 123delC).

Immunocytochemistry and Cell Size Determination

Dissociated iPSC-CMs differentiated for ~40 days were seeded at single-cell density on glass coverslips micro-patterned with 20-μm-wide gelatin lines as described previously (Birket et al., 2015). Four days later cells were fixed, permeablized, and labeled. Antibodies were as follows: anti-α-actinin (Sigma; A7811), anti-MLC-2V (Proteintech; 10906-1-AP), and anti-myomesin-3 (Proteintech; 17692-1-AP). Individual separated CMs were identified based on α-actinin labeling and then MLC-2V or myomesin-3 labeling was also imaged and recorded. Cell area was calculated using a thresholding mask based on α-actinin labeling.

Electrophysiology and Calcium Imaging

Dissociated iPSC-CMs differentiated for 30 days were seeded at single-cell density on fibronectin-coated glass coverslips and used for measurement 3–8 days later. Patch-clamp experiments were performed at 37°C and cells were continuously perfused with Tyrode's buffer. Only spontaneously active single cells were measured. Calcium imaging was performed on monolayers of iPSC-CMs using the Cal-520 assay as outlined in the product information sheet provided with the Hamamatsu FDSS *μCell* (Hamamatsu Photonics, Hamamatsu, Japan). Assay buffer was made from Hank's balanced saline solution buffered with 20 mM HEPES. The caffeine response was assessed by injecting 10 μL of caffeine at 50 mM resulting in a final concentration of 10 mM. Fluorescence measurements are represented as relative fluorescence unit. For each experiment, spontaneous calcium transients were analyzed over 2 min. Frequency, basal fluorescence (F), amplitude (ΔF/F), peak width at 90% of recovery (PWD-90%), corrected peak width (corrected PWD-90%), calculated by Fridericia's formula and rising slope (30%–70%) were measured (exemplified in Figure S1).

Proteomics

See Supplemental Information.

Western Blot

Ten or 30 μg of cell lysate, or secretome protein isolate, was loaded onto a 4%–20% SDS-PAGE gel (Bio-Rad, no. 5678094) and transferred to a PVDF membrane using a Trans-Blot Turbo Transfer Pack (Bio-Rad, no. 170–4157). Membranes were blocked in 5% milk in TBS-T and incubated in primary antibody overnight at 4°C. Antibodies and their dilutions were as follows: Anti-LIMP-2 (Abcam, ab176317; 1:1,000), anti-HSP70-2 (Abcam, ab108416; 1:1,000), anti-calsequestrin 2 (Proteintech, 18422-1-AP; 1:1,000), anti-GCase (internal; 1:500), anti-LAMP-2 (Santa-Cruz, Sc-18822; 1:500), anti-GLA (Abcam, ab168341; 1:1,000), anti-CTSF (Abcam, ab200650; 1:500), anti-collagen I (SouthernBiotech, 1310-01; 1:1,000), anti-collagen IV (Novocastra, NCL-COLL-IV; 1:1,000), anti-GAPDH (Abcam; 1:3,000). Horseradish peroxidase (HRP)-conjugated secondary antibodies were as follows: anti-mouse IgG(H + L) HRP (Thermo Fisher Scientific, no. 32230; 1:10,000) and anti-rabbit IgG(H + L) HRP (Thermo Fisher Scientific, no. 32460; 1:10,000).

Lentiviral Transduction

SCARB2 was overexpressed by lentivirus produced using a pLenti CMV/TO Puro DEST vector (Addgene plasmid no. 17452). The empty vector, or vector with a GFP insert, were used as controls. Lentiviral particles were concentrated using ultracentrifugation and used to transduce CMs at the time of their purification on day 14. Transduced cells were selected with 0.75 μg/mL puromycin and then cultured as normal. Conditioned medium was collected 5–8 days after transduction of CMs or 293FT cells.

SUPPLEMENTAL INFORMATION

Supplemental Information can be found online at https://doi.org/10.1016/j.stemcr.2019.07.004.

AUTHOR CONTRIBUTIONS

Conceptualization, M.J.B., J.M.I., and C.O.; Investigation, M.J.B., S.R., A.D.A., V.L., P.C., N.R., and B.B.; Writing – Original Draft, M.J.B.; Writing – Review & Editing, M.J.B., N.A.H., C.O., and

J.M.I.; Resources, M.L., G.R., S.V., B.W., and N.A.H.; Supervision, B.B., J.C.G., V.M., J.P.L., N.A.H., C.O., and J.M.I.

ACKNOWLEDGMENTS

We thank Dr Veeresha Yaligara, Dr Michel Didier, and Dr Oliva Petra for useful discussions. M.J.B. was supported by a Presidential Fellowship at The University of Manchester. N.H. was supported by Wellcome grants 088566 and 097820, and MRC grants MR/000638/1 and MR/S036121/1. M.L. was supported by a BHF PhD Studentship.

Received: March 6, 2018
Revised: July 4, 2019
Accepted: July 5, 2019
Published: August 1, 2019

REFERENCES

Altarescu, G., Goldfarb, L., Park, K.-Y., Kaneski, C., Jeffries, N., Litvak, S., Nagle, J., and Schiffmann, R. (2001). Identification of fifteen novel mutations and genotype–phenotype relationship in Fabry disease. Clin. Genet. *60*, 46–51.

Andrei, C., Dazzi, C., Lotti, L., Torrisi, M.R., Chimini, G., and Rubartelli, A. (1999). The secretory route of the leaderless protein interleukin 1 beta involves exocytosis of endolysosome-related vesicles. Mol. Biol. Cell *10*, 1463–1475.

Birket, M.J., Ribeiro, M.C., Kosmidis, G., Ward, D., Leitoguinho, A.R., van de Pol, V., Dambrot, C., Devalla, H.D., Davis, R.P., Mastroberardino, P.G., et al. (2015). Contractile defect caused by mutation in MYBPC3 revealed under conditions optimized for human PSC-cardiomyocyte function. Cell Rep. *13*, 733.

Brozzi, A., Urbanelli, L., Luc Germain, P., Magini, A., and Emiliani, C. (2013). hLGDB: a database of human lysosomal genes and their regulation. Database (Oxford) *2013*. https://doi.org/10.1093/database/bat024.

Chien, Y., Chien, C.-S., Chiang, H.-C., Huang, W.-L., Chou, S.-J., Chang, W.-C., Chang, Y.-L., Leu, H.-B., Chen, K.-H., Wang, K.-L., et al. (2016). Interleukin-18 deteriorates Fabry cardiomyopathy and contributes to the development of left ventricular hypertrophy in Fabry patients with GLA IVS4+919 G>A mutation. Oncotarget *7*, 87161–87179.

Chimenti, C., Hamdani, N., Boontje, N.M., DeCobelli, F., Esposito, A., Bronzwaer, J.G.F., Stienen, G.J.M., Russo, M.A., Paulus, W.J., Frustaci, A., et al. (2008). Myofilament degradation and dysfunction of human cardiomyocytes in Fabry disease. Am. J. Pathol. *172*, 1482–1490.

Choi, L., Vernon, J., Kopach, O., Minett, M.S., Mills, K., Clayton, P.T., Meert, T., and Wood, J.N. (2015). The Fabry disease-associated lipid Lyso-Gb3 enhances voltage-gated calcium currents in sensory neurons and causes pain. Neurosci. Lett. *594*, 163–168.

Choi, S., Kim, J.A., Na, H.-Y., Cho, S.-E., Park, S., Jung, S.-C., and Suh, S.H. (2014). Globotriaosylceramide induces lysosomal degradation of endothelial $K_{Ca}3.1$ in Fabry disease. Arterioscler. Thromb. Vasc. Biol. *34*, 81–89.

Chou, S.-J., Yu, W.-C., Chang, Y.-L., Chen, W.-Y., Chang, W.-C., Chien, Y., Yen, J.-C., Liu, Y.-Y., Chen, S.-J., Wang, C.-Y., et al. (2017). Energy utilization of induced pluripotent stem cell-derived cardiomyocyte in Fabry disease. Int. J. Cardiol. *232*, 255–263.

Chu, G., Egnaczyk, G.F., Zhao, W., Jo, S.-H., Fan, G.-C., Maggio, J.E., Xiao, R.-P., and Kranias, E.G. (2004). Phosphoproteome analysis of cardiomyocytes subjected to β-adrenergic stimulation: identification and characterization of a cardiac heat shock protein p20. Circ. Res. *94*, 184–193.

Daugaard, M., Rohde, M., and Jäättelä, M. (2007). The heat shock protein 70 family: highly homologous proteins with overlapping and distinct functions. FEBS Lett. *581*, 3702–3710.

Genomics England. (2017). The 100,000 Genomes Project Protocol v3 (Genomics England) https://doi.org/10.6084/m9.figshare.4530893.v3.

Doroudgar, S., and Glembotski, C.C. (2011). The cardiomyokine story unfolds: investigating stress-induced protein secretion in the heart. Trends Mol. Med. *17*, 207–214.

Eng, C.M., Guffon, N., Wilcox, W.R., Germain, D.P., Lee, P., Waldek, S., Caplan, L., Linthorst, G.E., and Desnick, R.J.; International Collaborative Fabry Disease Study Group (2001). Safety and efficacy of recombinant human alpha-galactosidase A replacement therapy in Fabry's disease. N. Engl. J. Med. *345*, 9–16.

Fan, G.-C., and Kranias, E.G. (2011). Small heat shock protein 20 (HspB6) in cardiac hypertrophy and failure. J. Mol. Cell. Cardiol. *51*, 574–577.

Ferraz, M.J., Kallemeijn, W.W., Mirzaian, M., Herrera Moro, D., Marques, A., Wisse, P., Boot, R.G., Willems, L.I., Overkleeft, H.S., and Aerts, J.M. (2014). Gaucher disease and Fabry disease: new markers and insights in pathophysiology for two distinct glycosphingolipidoses. Biochim. Biophys. Acta *1841*, 811–825.

Fonovic, M., and Turk, B. (2014). Cysteine cathepsins and extracellular matrix degradation. Biochim. Biophys. Acta *1840*, 2560–2570.

Itier, J.-M., Ret, G., Viale, S., Sweet, L., Bangari, D., Caron, A., Le-Gall, F., Bénichou, B., Leonard, J., Deleuze, J.-F., et al. (2014). Effective clearance of GL-3 in a human iPSC-derived cardiomyocyte model of Fabry disease. J. Inherit. Metab. Dis. *37*, 1013–1022.

King, J.H., Huang, C.L.-H., and Fraser, J.A. (2013). Determinants of myocardial conduction velocity: implications for arrhythmogenesis. Front. Physiol. *4*, 154.

Kirkegaard, T., Roth, A.G., Petersen, N.H.T., Mahalka, A.K., Olsen, O.D., Moilanen, I., Zylicz, A., Knudsen, J., Sandhoff, K., Arenz, C., et al. (2010). Hsp70 stabilizes lysosomes and reverts Niemann–Pick disease-associated lysosomal pathology. Nature *463*, 549–553.

Kirkegaard, T., Gray, J., Priestman, D.A., Wallom, K.-L., Atkins, J., Olsen, O.D., Klein, A., Drndarski, S., Petersen, N.H.T., Ingemann, L., et al. (2016). Heat shock protein-based therapy as a potential candidate for treating the sphingolipidoses. Sci. Transl. Med. *8*, 355ra118.

Kuramoto, Y., Naito, A.T., Tojo, H., Sakai, T., Ito, M., Shibamoto, M., Nakagawa, A., Higo, T., Okada, K., Yamaguchi, T., et al. (2018). Generation of Fabry cardiomyopathy model for drug screening using induced pluripotent stem cell-derived cardiomyocytes from a female Fabry patient. J. Mol. Cell. Cardiol. *121*, 256–265.

Kuronita, T., Eskelinen, E.-L., Fujita, H., Saftig, P., Himeno, M., and Tanaka, Y. (2002). A role for the lysosomal membrane protein LGP85 in the biogenesis and maintenance of endosomal and lysosomal morphology. J. Cell Sci. *115*, 4117–4131.

Linhart, A., and Elliott, P.M. (2007). The heart in Anderson-Fabry disease and other lysosomal storage disorders. Heart *93*, 528–535.

Maki, N., Komatsuda, A., Wakui, H., Oyama, Y., Kodama, T., Ohtani, H., Kigawa, A., Aiba, N., Imai, H., Motegi, M., et al. (2004). A nonsense mutation (R220X) in the alpha-galactosidase A gene causes typical Fabry disease in both genders. Clin. Nephrol. *61*, 185–190.

Mambula, S.S., and Calderwood, S.K. (2006). Heat shock protein 70 is secreted from tumor cells by a nonclassical pathway involving lysosomal endosomes. J. Immunol. *177*, 7849–7857.

Martin, T.P., Currie, S., and Baillie, G.S. (2014). The cardioprotective role of small heat-shock protein 20. Biochem. Soc. Trans. *42*, 270–273.

McDermott-Roe, C., Ye, J., Ahmed, R., Sun, X.-M., Serafin, A., Ware, J., Bottolo, L., Muckett, P., Canas, X., Zhang, J., et al. (2011). Endonuclease G is a novel determinant of cardiac hypertrophy and mitochondrial function. Nature *478*, 114–118.

Mehta, A., Clarke, J.T.R., Giugliani, R., Elliott, P., Linhart, A., Beck, M., and Sunder-Plassmann, G.; FOS Investigators (2009). Natural course of Fabry disease: changing pattern of causes of death in FOS - Fabry outcome survey. J. Med. Genet. *46*, 548–552.

Moran, M.T., Schofield, J.P., Hayman, A.R., Shi, G.P., Young, E., and Cox, T.M. (2000). Pathologic gene expression in Gaucher disease: up-regulation of cysteine proteinases including osteoclastic cathepsin K. Blood *96*, 1969–1978.

Nagueh, S.F. (2014). Anderson-Fabry disease and other lysosomal storage disorders. Circulation *130*, 1081–1090.

Namdar, M. (2016). Electrocardiographic changes and arrhythmia in Fabry disease. Front. Cardiovasc. Med. *3*, 7.

Namdar, M., Steffel, J., Vidovic, M., Brunckhorst, C.B., Holzmeister, J., Lüscher, T.F., Jenni, R., and Duru, F. (2011). Electrocardiographic changes in early recognition of Fabry disease. Heart *97*, 485–490.

Nanjappa, V., Thomas, J.K., Marimuthu, A., Muthusamy, B., Radhakrishnan, A., Sharma, R., Ahmad Khan, A., Balakrishnan, L., Sahasrabuddhe, N.A., Kumar, S., et al. (2014). Plasma Proteome Database as a resource for proteomics research: 2014 update. Nucleic Acids Res. *42*, D959–D965.

Nguyen, D.C., Hookway, T.A., Wu, Q., Jha, R., Preininger, M.K., Chen, X., Easley, C.A., Spearman, P., Deshpande, S.R., Maher, K., et al. (2014). Microscale generation of cardiospheres promotes robust enrichment of cardiomyocytes derived from human pluripotent stem cells. Stem Cell Reports *3*, 260–268.

Öörni, K., Sneck, M., Brömme, D., Pentikäinen, M.O., Lindstedt, K.A., Mäyränpää, M., Aitio, H., and Kovanen, P.T. (2004). Cysteine protease cathepsin F is expressed in human atherosclerotic lesions, is secreted by cultured macrophages, and modifies low density lipoprotein particles in vitro. J. Biol. Chem. *279*, 34776–34784.

Park, S., Kim, J.A., Joo, K.Y., Choi, S., Choi, E.-N., Shin, J.-A., Han, K.-H., Jung, S.-C., and Suh, S.H. (2011). Globotriaosylceramide leads to KCa3.1 channel dysfunction: a new insight into endothelial dysfunction in Fabry disease. Cardiovasc. Res. *89*, 290–299.

Petersen, T.N., Brunak, S., von Heijne, G., and Nielsen, H. (2011). SignalP 4.0: discriminating signal peptides from transmembrane regions. Nat. Methods *8*, 785–786.

Pieroni, M., Chimenti, C., Ricci, R., Sale, P., Russo, M.A., and Frustaci, A. (2003). Early detection of Fabry cardiomyopathy by tissue Doppler imaging. Circulation *107*, 1978–1984.

Pinkosky, S.L., Groot, P.H.E., Lalwani, N.D., and Steinberg, G.R. (2017). Targeting ATP-citrate lyase in hyperlipidemia and metabolic disorders. Trends Mol. Med. *23*, 1047–1063.

Platt, F.M. (2014). Sphingolipid lysosomal storage disorders. Nature *510*, 68–75.

Reczek, D., Schwake, M., Schröder, J., Hughes, H., Blanz, J., Jin, X., Brondyk, W., Van Patten, S., Edmunds, T., and Saftig, P. (2007). LIMP-2 is a receptor for lysosomal mannose-6-phosphate-independent targeting of β-glucocerebrosidase. Cell *131*, 770–783.

Ritchie, M.E., Phipson, B., Wu, D., Hu, Y., Law, C.W., Shi, W., and Smyth, G.K. (2015). Limma powers differential expression analyses for RNA-sequencing and microarray studies. Nucleic Acids Res. *43*, e47.

Schroen, B., Leenders, J.J., van Erk, A., Bertrand, A.T., van Loon, M., van Leeuwen, R.E., Kubben, N., Duisters, R.F., Schellings, M.W., Janssen, B.J., et al. (2007). Lysosomal integral membrane protein 2 is a novel component of the cardiac intercalated disc and vital for load-induced cardiac myocyte hypertrophy. J. Exp. Med. *204*, 1227.

Song, H.-Y., Chien, C.-S., Yarmishyn, A.A., Chou, S.-J., Yang, Y.-P., Wang, M.-L., Wang, C.-Y., Leu, H.-B., Yu, W.-C., Chang, Y.-L., et al. (2019). Generation of GLA-knockout human embryonic stem cell lines to model autophagic dysfunction and exosome secretion in Fabry disease-associated hypertrophic cardiomyopathy. Cells *8*. https://doi.org/10.3390/cells8040327.

Tachibana, A., Santoso, M.R., Mahmoudi, M., Shukla, P., Wang, L., Bennett, M., Goldstone, A.B., Wang, M., Fukushi, M., Ebert, A., et al. (2017). Paracrine effects of the pluripotent stem cell-derived cardiac myocytes salvage the injured myocardium. Circ. Res. *121*, e22–e36.

Thurberg, B.L., Fallon, J.T., Mitchell, R., Aretz, T., Gordon, R.E., and O'Callaghan, M.W. (2009). Cardiac microvascular pathology in Fabry disease. Circulation *119*, 2561–2567.

Uhlén, M., Fagerberg, L., Hallström, B.M., Lindskog, C., Oksvold, P., Mardinoglu, A., Sivertsson, Å., Kampf, C., Sjöstedt, E., Asplund, A., et al. (2015). Proteomics. Tissue-based map of the human proteome. Science *347*, 1260419.

Ye, J., Bates, N., Soteriou, D., Grady, L., Edmond, C., Ross, A., Kerby, A., Lewis, P.A., Adeniyi, T., Wright, R., et al. (2017). High quality clinical grade human embryonic stem cell lines derived from fresh discarded embryos. Stem Cell Res. Ther. *8*, 128.

Zhang, X., Wang, X., Zhu, H., Kranias, E.G., Tang, Y., Peng, T., Chang, J., and Fan, G.-C. (2012). Hsp20 functions as a novel cardiokine in promoting angiogenesis via activation of VEGFR2. PLoS One *7*, e32765.

Stem Cell Reports, Volume *13*

Supplemental Information

A Human Stem Cell Model of Fabry Disease Implicates LIMP-2 Accumulation in Cardiomyocyte Pathology

Matthew J. Birket, Sophie Raibaud, Miriam Lettieri, Antony D. Adamson, Valerie Letang, Pauline Cervello, Nicolas Redon, Gwenaelle Ret, Sandra Viale, Bing Wang, Bruno Biton, Jean-Claude Guillemot, Vincent Mikol, John P. Leonard, Neil A. Hanley, Cecile Orsini, and Jean-Michel Itier

Figure S1

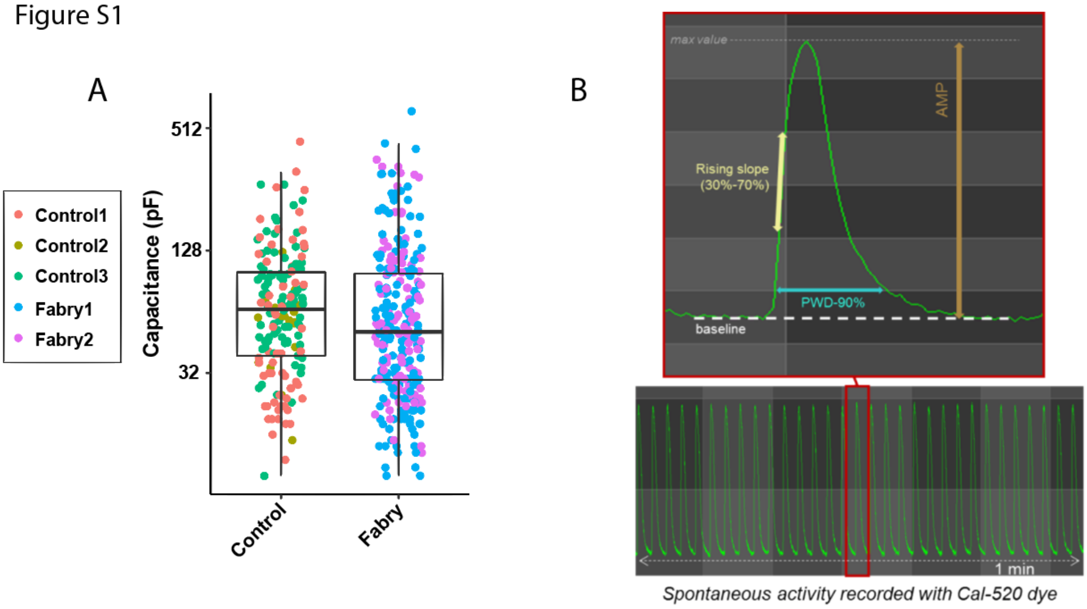
A
Control1
Control2
Control3
Fabry1
Fabry2
Capacitance (pF)
512
128
32
Control
Fabry
B
max value
AMP
Rising slope
(30%-70%)
PWD-90%
baseline
1 min
Spontaneous activity recorded with Cal-520 dye

Figure S2

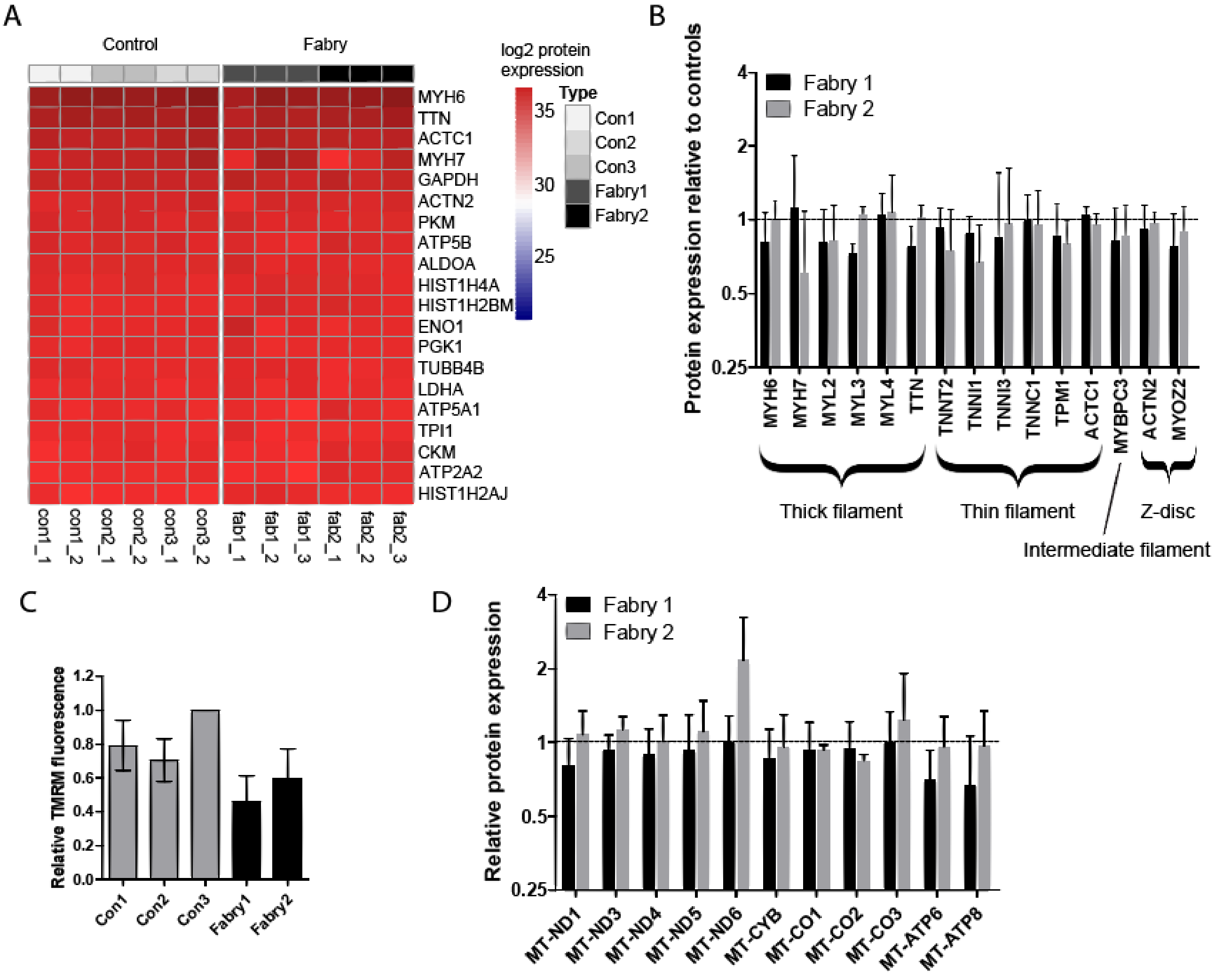

A
Control
Fabry
log2 protein expression
35
30
25
Type
Con1
Con2
Con3
Fabry1
Fabry2
MYH6
TTN
ACTC1
MYH7
GAPDH
ACTN2
PKM
ATP5B
ALDOA
HIST1H4A
HIST1H2BM
ENO1
PGK1
TUBB4B
LDHA
ATP5A1
TPI1
CKM
ATP2A2
HIST1H2AJ
con1_1
con1_2
con2_1
con2_2
con3_1
con3_2
fab1_1
fab1_2
fab1_3
fab2_1
fab2_2
fab2_3
B
Protein expression relative to controls
Fabry 1
Fabry 2
MYH6
MYH7
MYL2
MYL3
MYL4
TTN
TNNT2
TNNI1
TNNI3
TNNC1
TPM1
ACTC1
MYBPC3
ACTN2
MYOZ2
Thick filament
Thin filament
Z-disc
Intermediate filament
C
Relative TMRM fluorescence
Con1
Con2
Con3
Fabry1
Fabry2
D
Relative protein expression
Fabry 1
Fabry 2
MT-ND1
MT-ND3
MT-ND4
MT-ND5
MT-ND6
MT-CYB
MT-CO1
MT-CO2
MT-CO3
MT-ATP6
MT-ATP8

Figure S3

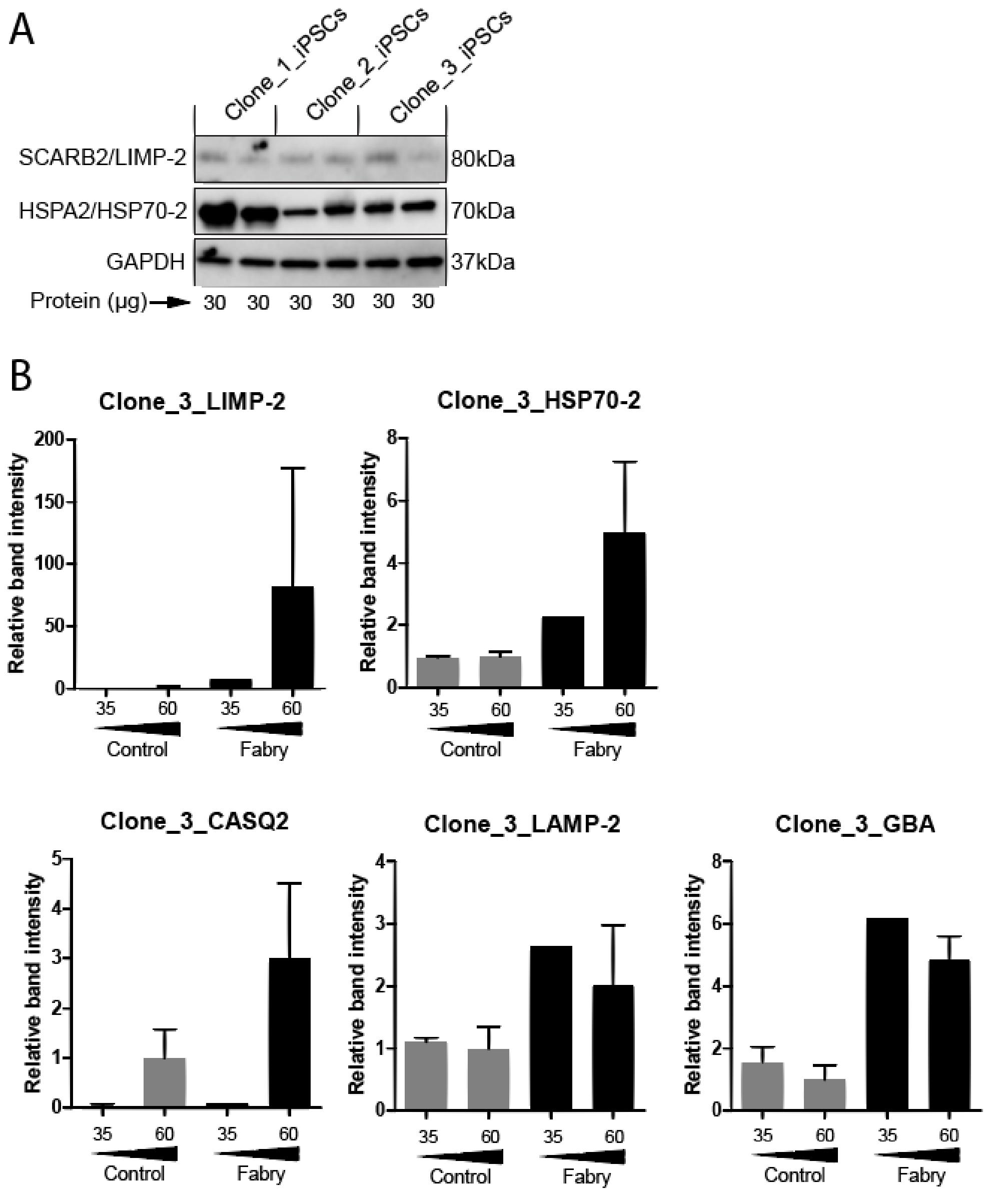

A
Clone_1_iPSCs
Clone_2_iPSCs
Clone_3_iPSCs
SCARB2/LIMP-2
80kDa
HSPA2/HSP70-2
70kDa
GAPDH
37kDa
Protein (µg)
30 30 30 30 30 30
B
Clone_3_LIMP-2
Clone_3_HSP70-2
Clone_3_CASQ2
Clone_3_LAMP-2
Clone_3_GBA
Relative band intensity
35 60 35 60
Control
Fabry

Figure S4

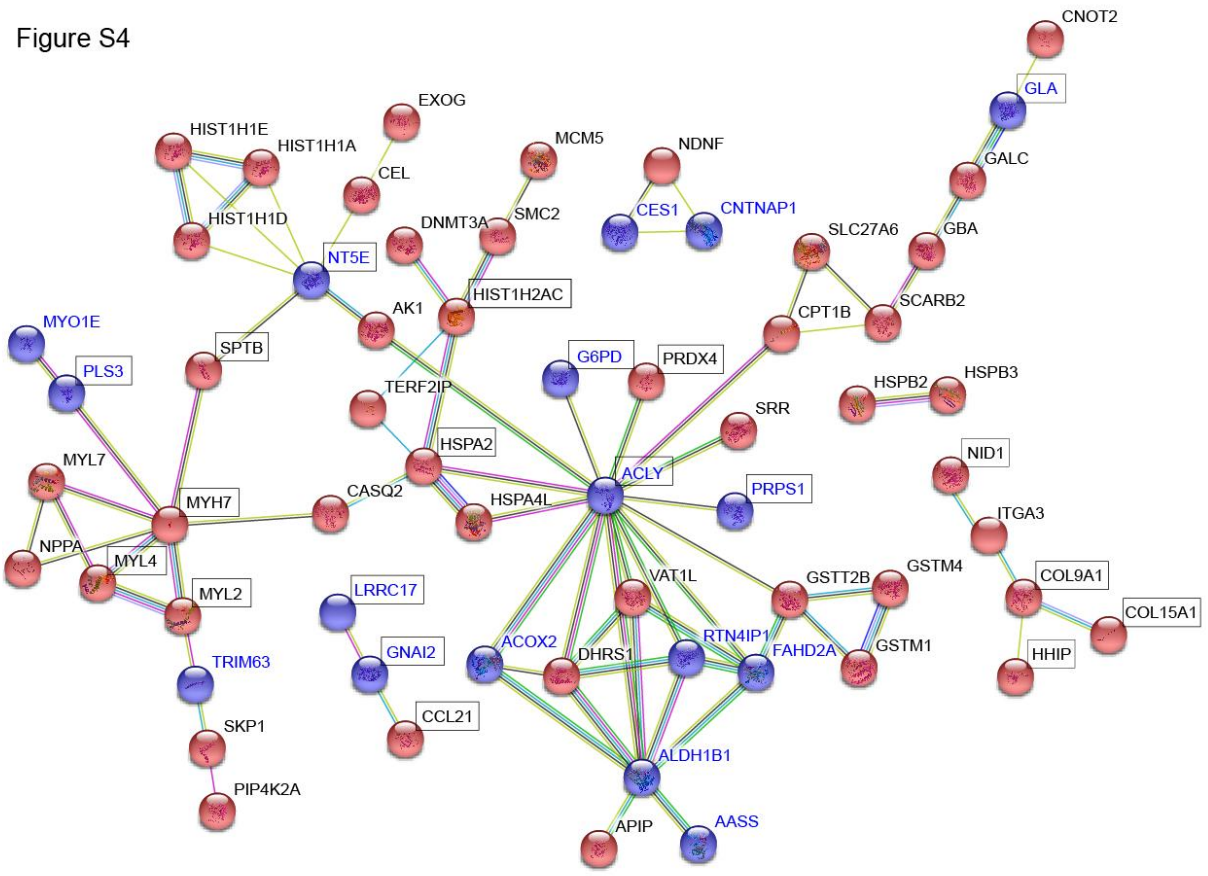
CNOT2
GLA
GALC
GBA
SCARB2
SLC27A6
CPT1B
EXOG
CEL
HIST1H1E
HIST1H1A
HIST1H1D
NT5E
MCM5
SMC2
DNMT3A
HIST1H2AC
AK1
NDNF
CES1
CNTNAP1
MYO1E
PLS3
SPTB
TERF2IP
G6PD
PRDX4
SRR
HSPB2
HSPB3
HSPA2
ACLY
PRPS1
NID1
ITGA3
MYL7
MYH7
CASQ2
HSPA4L
NPPA
MYL4
MYL2
VAT1L
GSTT2B
GSTM4
COL9A1
COL15A1
LRRC17
GNAI2
ACOX2
DHRS1
RTN4IP1
FAHD2A
GSTM1
HHIP
TRIM63
CCL21
SKP1
ALDH1B1
PIP4K2A
APIP
AASS

Figure S5

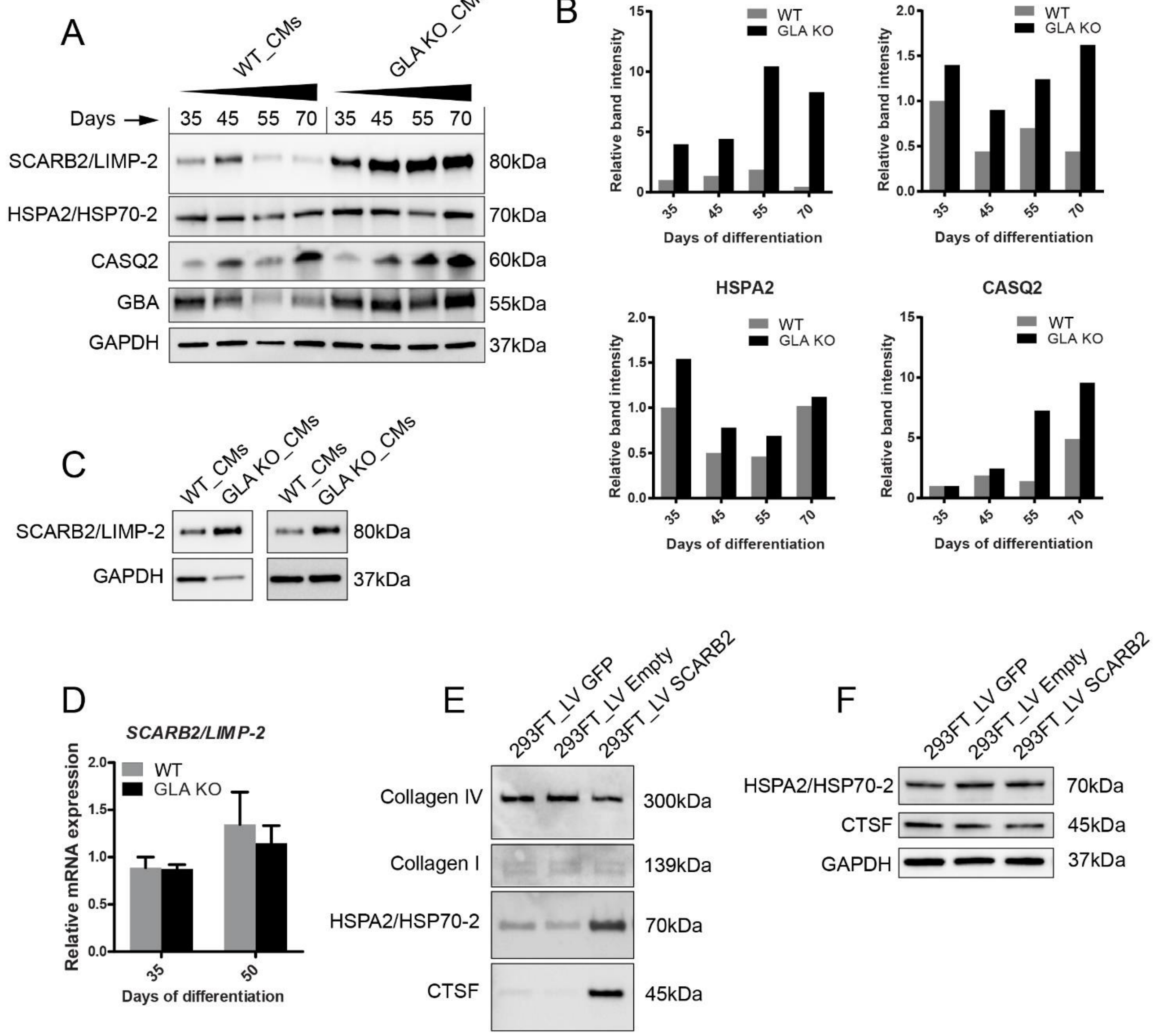

A
WT_CMs
GLA KO_CMs
Days
35 45 55 70 35 45 55 70
SCARB2/LIMP-2
80kDa
HSPA2/HSP70-2
70kDa
CASQ2
60kDa
GBA
55kDa
GAPDH
37kDa
B
LIMP-2
GBA
HSPA2
CASQ2
WT
GLA KO
Relative band intensity
Days of differentiation
C
WT_CMs
GLA KO_CMs
SCARB2/LIMP-2
80kDa
GAPDH
37kDa
D
SCARB2/LIMP-2
Relative mRNA expression
Days of differentiation
E
293FT_LV GFP
293FT_LV Empty
293FT_LV SCARB2
Collagen IV
300kDa
Collagen I
139kDa
HSPA2/HSP70-2
70kDa
CTSF
45kDa
F
HSPA2/HSP70-2
70kDa
CTSF
45kDa
GAPDH
37kDa

Figure S1. Cardiomyocyte capacitance and calcium transient measurements. A) Capacitance measurements of single cardiomyocytes from 3 control and 2 Fabry iPSC lines. Boxes indicate the median ± quartiles and whiskers show the range minus outliers. B) Example Cal-520 imaging measurements and quantification of calcium transient parameters. PWD-90% = peak width at 90% of recovery, and AMP = amplitude (ΔF/F).

Figure S2. Extended proteome and functional analysis. A) Heatmap of the top 20 most highly expressed proteins by average abundance. B) Relative levels of sarcomeric proteins in Fabry-CMs compared to controls. The sarcomeric location of each protein is highlighted below. C) Normalized TMRM fluorescence after probe equilibration. D) Relative levels of mtDNA-encoded proteins in Fabry-CMs compared to controls. Bars show mean ± s.d. for 5 independent biological replicates for each cell line.

Figure S3. Extended Western blots and analyses. A) Western blots of three different iPSC clones derived from Fabry1 patient cells. Clone 1 is used throughout the rest of the study. B) Quantification of Western blots in **Figure 4E** as control and Fabry ordered by differentiation time, calculated as relative band density normalized to GAPDH levels. Each lysate was produced from an independent experiment. Bars show mean ± s.d.

Figure S4. Results of STRING analysis, showing potential protein-protein-interactions, as run on the 113 differentially expressed cellular and secreted proteins from **Table S3** and **Table S4**. Up- and Down-regulated proteins in Fabry-CMs are shown in red and blue respectively. Proteins differentially expressed in the secretome are highlighted in squares.

Figure S5. Extended Western blots from *GLA* KO hPSC-CMs and LIMP-2 overexpressing cells. A) Western blots of MAN13 control (WT_CMs) and paired *GLA* knockout (GLA KO_CMs) hESC-derived CMs taken at four timepoints from a single differentiation. B) Quantification of Western blots in 'A'. C) Western blots of con3 (WT_CMs) and paired *GLA* knockout iPSC-derived CMs (day 35) from two separate experiments. D) *SCARB2* mRNA expression in MAN13 control and paired *GLA* knockout hESC-derived CMs, taken at two timepoints through a single differentiation. E) Western blot of secretome samples from two independent experiments/transductions showing the impact of LIMP-2 overexpression on protein secretion by 293FT cells. F) Western blot of 293FT cells showing no impact of LIMP-2 overexpression on cellular marker expression.

Table S1.

	Controls (n=136)	Fabry (n=213)
**MDP (mV)**	-63.6±1.2	-69.1±0.8*
**dV/dt_{max} (V/s)**	41.6±4.5	64.6±4.1*
**APA (mV)**	89.9±2.1	88.9±1.3
**APD_{90} (ms)**	476±35	276±19*
**Frequency (Hz)**	1.2±0.1	1.6±0.1*
**Capacitance (Cm)**	80.3±4.8	79.3±4.9
***I*Na (pA/pF)**	60.3±3.5	108.7±5.5*

Table S2.

	Controls (n = 8)	Fabry (n = 8)
**Frequency (Hz)**	0.8±0.1	0.9±0.2
**Peak width duration 90% (ms)**	830±8	581±7
**Corrected peak width duration 90% (ms)**	777±4	556±3
**Rising slope (30-70%)**	20±2	57±2
**Baseline fluorescence (F)**	995±7	917±7
**Amplitude (ΔF/*F*)**	2.2±0.1	3.2±0.4
**Caffeine-induced amplitude (ΔF/*F*)**	2.7±0.4	3.9±0.4

Table S4.

			Log2 FC				
**Uniprot**	**Protein name**	**Gene name**	**Fabry1**	**Fabry2**	**AvExp**	**p.value**	**adj.P.Val**
Q96GE9	Transmembrane protein 261	TMEM261	4.17	4.10	21.11	1.18E-08	4.97E-05
P09488	Glutathione S-transferase Mu 1	GSTM1	3.84	4.05	21.24	9.44E-07	0.000991
Q02539	Histone H1.1	HIST1H1A	3.97	2.67	22.36	8.16E-05	0.009957
Q9HCJ6	Synaptic vesicle membrane protein VAT-1 homolog-like	VAT1L	2.73	2.91	23.57	3.65E-05	0.006139
Q13601	KRR1 small subunit processome component homolog	KRR1	2.68	2.92	20.41	7.67E-06	0.002479
P54652	Heat shock-related 70 kDa protein 2	HSPA2	3.33	2.09	25.79	1.58E-06	0.001325
Q9Y2C4	Nuclease EXOG, mitochondrial	EXOG	2.32	3.09	21.59	0.000557	0.022275
P0CG30	Glutathione S-transferase theta-2B	GSTT2B	2.03	3.10	24.49	2.87E-06	0.001471
Q5T6V5	UPF0553 protein C9orf64	C9orf64	2.77	2.17	22.45	0.000777	0.025588
Q7L5Y1	Mitochondrial enolase superfamily member 1	ENOSF1	2.49	2.06	19.92	0.001314	0.033058
Q8N1Q1	Carbonic anhydrase 13	CA13	2.07	1.86	22.39	1.79E-05	0.003694
O75764	Transcription elongation factor A protein 3	TCEA3	1.46	2.04	21.14	5.55E-05	0.007746
O95347	Structural maintenance of chromosomes protein 2	SMC2	1.58	1.93	20.44	1.90E-05	0.003694
Q5TDH0	Protein DDI1 homolog 2	DDI2	1.65	1.71	23.28	0.000422	0.019693
Q9H7C9	Mth938 domain-containing protein	AAMDC	1.60	1.48	21.99	3.49E-07	0.000732
O14958	Calsequestrin-2	CASQ2	1.14	1.91	27.71	0.002653	0.045901
Q9NZN8	CCR4-NOT transcription complex subunit 2	CNOT2	1.30	1.49	20.41	0.002426	0.044918
Q14331	Protein FRG1	FRG1	1.55	1.14	20.32	0.00116	0.030633
Q14108	Lysosome membrane protein 2	SCARB2	1.48	1.21	27.31	4.90E-06	0.001915
Q9NYB0	Telomeric repeat-binding factor 2-interacting protein 1	TERF2IP	1.25	1.43	20.24	0.002794	0.046567
Q96LJ7	Dehydrogenase/reductase SDR family member 1	DHRS1	1.68	0.94	21.09	0.000228	0.014663
Q92523	Carnitine O-palmitoyltransferase 1, muscle isoform	CPT1B	1.27	1.12	25.82	1.70E-05	0.003694
Q9UBX1	Cathepsin F	CTSF	0.77	1.61	20.80	0.001077	0.029489
Q9Y6M5	Zinc transporter 1	SLC30A1	1.09	1.23	21.43	0.002451	0.044918
P16402	Histone H1.3	HIST1H1D	1.49	0.75	23.62	0.000982	0.028905
O95757	Heat shock 70 kDa protein 4L	HSPA4L	1.53	0.57	23.73	0.000372	0.018275
P33992	DNA replication licensing factor MCM5	MCM5	1.40	0.61	25.43	0.002254	0.043628
P05114	Non-histone chromosomal protein HMG-14	HMGN1	1.24	0.71	19.43	0.000352	0.017998
P46937	Transcriptional coactivator YAP1	YAP1	0.87	1.06	22.17	0.000112	0.010898
Q53HC9	Protein TSSC1	TSSC1	1.05	0.88	21.42	0.002837	0.046689
Q9GZT4	Serine racemase	SRR	0.99	0.83	24.84	3.05E-05	0.005329
P54252	Ataxin-3	ATXN3	0.87	0.89	19.88	1.93E-05	0.003694
P29558	RNA-binding motif, single-stranded-interacting protein 1	RBMS1	1.00	0.76	21.49	0.000193	0.013737
Q01449	Myosin regulatory light chain 2, atrial isoform	MYL7	1.03	0.71	27.95	0.001927	0.040819
P04062	Glucosylceramidase	GBA	0.69	0.96	26.67	8.77E-05	0.010235

Q96FV2	Secernin-2	SCRN2	0.79	0.78	24.37	0.000151	0.012343
Q9H008	Phospholysine phosphohistidine inorganic pyrophosphate phosphatase	LHPP	0.92	0.65	23.22	0.002051	0.041287
P00167	Cytochrome b5	CYB5A	0.79	0.74	24.41	0.000157	0.012431
Q12988	Heat shock protein beta-3	HSPB3	0.88	0.63	24.91	0.00238	0.044421
Q96E39	RNA binding motif protein, X-linked-like-1	RBMXL1	0.87	0.64	23.17	0.000735	0.024936
P10412	Histone H1.4	HIST1H1E	0.98	0.50	27.81	0.001939	0.040819
Q9HA64	Ketosamine-3-kinase	FN3KRP	0.84	0.64	23.16	0.00023	0.014663
Q8TE77	Protein phosphatase Slingshot homolog 3	SSH3	0.81	0.67	20.37	3.00E-05	0.005329
Q05084	Islet cell autoantigen 1	ICA1	0.66	0.80	21.97	0.00014	0.012009
Q03013	Glutathione S-transferase Mu 4	GSTM4	0.58	0.88	25.07	0.000715	0.024806
P48426	Phosphatidylinositol 5-phosphate 4-kinase type-2 alpha	PIP4K2A	0.78	0.64	23.31	4.79E-05	0.006934
P63208	S-phase kinase-associated protein 1	SKP1	0.66	0.73	22.41	0.000235	0.014746
P54803	Galactocerebrosidase	GALC	0.77	0.62	24.16	0.000121	0.011511
Q9HBK9	Arsenite methyltransferase	AS3MT	0.65	0.73	24.81	2.48E-06	0.001471
Q9Y6K1	DNA (cytosine-5)-methyltransferase 3A	DNMT3A	0.65	0.51	23.83	0.000525	0.021418
Q7Z7K0	COX assembly mitochondrial protein homolog	CMC1	0.57	0.57	22.56	0.002471	0.044918
P00568	Adenylate kinase isoenzyme 1	AK1	0.51	0.60	28.90	0.000445	0.020309
Q16082	Heat shock protein beta-2	HSPB2	0.52	0.53	26.85	0.000173	0.012774
Q9BXW7	Cat eye syndrome critical region protein 5	CECR5	-0.54	-0.50	27.00	0.001837	0.040402
Q14249	Endonuclease G, mitochondrial	ENDOG	-0.72	-0.51	24.95	0.000108	0.010898
Q12965	Unconventional myosin-Ie	MYO1E	-0.54	-0.71	22.16	5.72E-05	0.007746
Q99424	Peroxisomal acyl-coenzyme A oxidase 2	ACOX2	-0.69	-0.64	22.05	0.000266	0.015963
Q86VU5	Catechol O-methyltransferase domain-containing protein 1	COMTD1	-0.58	-0.85	22.47	9.51E-05	0.010575
Q8WWV3	Reticulon-4-interacting protein 1, mitochondrial	RTN4IP1	-0.61	-0.87	24.57	3.15E-06	0.001471
Q969Q1	E3 ubiquitin-protein ligase TRIM63	TRIM63	-0.52	-1.01	22.53	0.000344	0.017859
**Q96GX9**	Methylthioribulose-1-phosphate dehydratase	APIP	-0.82	-0.73	23.54	0.000461	0.020309
**Q96GK7**	Fumarylacetoacetate hydrolase domain-containing protein 2A	FAHD2A	-0.73	-0.82	26.08	6.23E-07	0.000872
**P26006**	Integrin alpha-3	ITGA3	-0.69	-1.17	24.53	0.000469	0.020309
**P30837**	Aldehyde dehydrogenase X, mitochondrial	ALDH1B1	-1.34	-1.03	26.61	6.70E-06	0.002345
**Q9Y2P4**	Long-chain fatty acid transport protein 6	SLC27A6	-1.44	-1.17	26.53	0.001958	0.040819
**Q9UDR5**	Alpha-aminoadipic semialdehyde synthase, mitochondrial	AASS	-1.64	-1.09	25.38	0.00017	0.012774
**Q9HB40**	Retinoid-inducible serine carboxypeptidase	SCPEP1	-1.95	-1.16	21.23	0.000731	0.024936
**Q9BQT8**	Mitochondrial 2-oxodicarboxylate carrier	SLC25A21	-1.59	-1.76	20.43	0.000468	0.020309
**P35556**	Fibrillin-2	FBN2	-1.88	-1.61	25.84	0.000866	0.026645
**P78357**	Contactin-associated protein 1	CNTNAP1	-2.41	-1.35	24.00	0.000744	0.024993
**P06280**	Alpha-galactosidase A	GLA	-2.13	-2.59	19.60	0.000324	0.017347
**P23141**	Liver carboxylesterase 1	CES1	-3.68	-3.27	20.87	2.70E-06	0.001471

Table S5.

GO biological process complete	Homo sapiens - REFLIST (21042)	Fabry_set (72)	Fabry_set (expected)	Fabry_set (over/under)	Fabry_set (fold Enrichment)	Fabry_set (P-value)
glycosylceramide catabolic process (GO:0046477)	6	3	0.02	+	> 100	1.18E-02
metabolic process (GO:0008152)	9949	54	34.04	+	1.59	1.35E-02
Unclassified (UNCLASSIFIED)	3561	2	12.18	-	< 0.2	0.00E+00

Table S6.

GO cellular component complete	Homo sapiens - REFLIST (21042)	Fabry_set (72)	Fabry_set (expected)	Fabry_set (over/under)	Fabry_set (fold Enrichment)	Fabry_set (P-value)
lysosomal lumen (GO:0043202)	93	5	0.32	+	15.71	2.48E-02
mitochondrion (GO:0005739)	1731	19	5.92	+	3.21	5.35E-03
cytoplasm (GO:0005737)	11364	57	38.88	+	1.47	1.08E-02
intracellular part (GO:0044424)	14239	64	48.72	+	1.31	3.57E-02
Unclassified (UNCLASSIFIED)	2449	3	8.38	-	0.36	0.00E+00

Table S7.

UniProt	Protein name	Gene name	Log2 FC Fabry1	Log2 FC Fabry2	AvExp	p_value	adj.P.Val
**Q9UBX1**	Cathepsin F	CTSF	5.08	4.79	25.06	5.29E-07	0.000436
**O14558**	Heat shock protein beta-6	HSPB6	4.81	4.16	21.48	7.44E-07	0.000436
**P54652**	Heat shock-related 70 kDa protein 2	HSPA2	5.11	3.17	21.57	1.37E-06	0.000483
**P39059**	Collagen alpha-1(XV) chain	COL15A1	5.98	1.47	22.52	0.001189	0.043607
**P20396**	Pro-thyrotropin-releasing hormone	TRH	3.07	2.96	28.36	1.48E-05	0.004347
**P20849**	Collagen alpha-1(IX) chain	COL9A1	3.65	2.25	25.93	2.51E-05	0.006315
**O00585**	C-C motif chemokine 21	CCL21	4.32	1.37	23.98	0.000866	0.033867
**P19835**	Bile salt-activated lipase	CEL	1.14	3.12	29.08	0.000226	0.018114
**Q8N436**	Inactive carboxypeptidase-like protein X2	CPXM2	2.80	1.39	26.21	0.000931	0.035611
**Q8TB73**	Protein NDNF	NDNF	2.92	1.04	26.90	0.000128	0.017251
**Q93077**	Histone H2A type 1-C	HIST1H2AC	2.33	1.46	22.96	0.000563	0.029984
**P01160**	Natriuretic peptides A	NPPA	2.09	0.98	30.49	0.000167	0.017251
**P11277**	Spectrin beta chain, erythrocytic	SPTB	1.19	1.70	24.13	0.000487	0.028591
**P10916**	Myosin regulatory light chain 2, ventricular/cardiac muscle isoform	MYL2	0.97	1.38	28.25	0.000206	0.017892
**Q9NZN3**	EH domain-containing protein 3	EHD3	0.75	1.40	23.77	0.000267	0.01947
**P12829**	Myosin light chain 4	MYL4	0.97	1.13	29.87	0.000158	0.017251
**Q13162**	Peroxiredoxin-4	PRDX4	1.16	0.83	23.27	1.32E-06	0.000483
**Q9H3G5**	Probable serine carboxypeptidase CPVL	CPVL	0.66	1.31	27.91	0.000574	0.029984
**P14543**	Nidogen-1	NID1	1.11	0.57	30.64	0.000175	0.017251
**P12883**	Myosin-7	MYH7	0.70	0.92	30.83	0.001367	0.047176
**P60891**	Ribose-phosphate pyrophosphokinase 1	PRPS1	-0.78	-0.62	23.82	0.001496	0.049663
**Q16643**	Drebrin	DBN1	-0.53	-0.95	24.70	0.001313	0.046801
**P04899**	Guanine nucleotide-binding protein G(i) subunit alpha-2	GNAI2	-0.63	-0.86	23.88	4.26E-05	0.009374
**P31949**	Protein S100-A11	S100A11	-0.87	-0.94	27.48	0.000772	0.031604
**P53396**	ATP-citrate synthase	ACLY	-0.75	-1.10	26.19	0.000211	0.017892
**P13797**	Plastin-3	PLS3	-0.97	-1.24	27.69	0.000975	0.036496
**P11413**	Glucose-6-phosphate 1-dehydrogenase	G6PD	-1.59	-1.60	25.12	0.000286	0.01947
**Q13219**	Pappalysin-1	PAPPA	-1.33	-1.86	27.65	0.000417	0.025335
**P21589**	5-nucleotidase	NT5E	-3.50	-1.88	21.19	0.000687	0.031
**Q8IWU6**	Extracellular sulfatase Sulf-1	SULF1	-3.00	-2.44	22.75	0.000715	0.031478
**Q9UHG2**	ProSAAS	PCSK1N	-1.91	-3.58	20.59	0.000176	0.017251
**Q96QV1**	Hedgehog-interacting protein	HHIP	-2.47	-3.05	22.14	0.000684	0.031
**Q8N6Y2**	Leucine-rich repeat-containing protein 17	LRRC17	-2.45	-4.07	22.08	0.000283	0.01947
**P06280**	Alpha-galactosidase A	GLA	-4.52	-4.27	20.51	5.39E-09	9.48E-06

Table S1. Action potential parameters and sodium current of single spontaneously active cardiomyocytes. Data are mean ± SEM; n = number of cells; MDP = maximal diastolic potential; dV/dt_{max} = maximal upstroke velocity; APA = action potential amplitude; APD_{90} = action potential duration at 90% repolarization; *I*Na = peak sodium current normalized by capacitance. * $p < 0.05$ unpaired t-test.

Table S2. Calcium transient parameters and response to caffeine. Data are mean ± SEM; n = number of experimental wells measured.

Table S3. Complete cardiomyocyte proteome and secretome data in excel format. Tab 1 ('Exp1_proteome') shows data from the first cell proteomics experiment and tab 2 ('Exp2_proteome') shows data from the second experiment. Tab 3 ('Exp1_secretome') shows data from the first secretome experiment, tab 4 ('Exp2_secretome') shows data from the second secretome experiment and tab 5 ('Exp3_secretome') shows data from the third secretome experiment. Protein levels are shown as log2 values. AvExp = the mean expression across all samples in the run. For sample names, con = control lines, and fab = Fabry lines, and the number before the underscore indicates the cell line and the number after indicates the biological replicate.

Table S4. Differentially expressed proteins from complete cardiomyocyte proteome data identified using a filtering approach. Log2FC = log2 fold-change of Fabry samples (mean of 5 biological replicates for each line) compared to controls; AvExp = average expression across all samples as log2 value; t = t statistic; adj.P.Val = p value with Benjamini-Hochberg adjustment.

Table S5. Gene ontology enrichment analysis 2: cellular component overrepresentation test results. The 72 differentially expressed proteins from **Table S4** were analysed using PANTHER (Mi et al., 2017) to test for cellular component enrichment against the whole genome.

Table S6. Gene ontology (GO) enrichment analysis 1: biological process overrepresentation test results. The 72 differentially expressed proteins from **Table S4** were analysed using PANTHER (Mi et al., 2017) to test for process enrichment against the whole genome.

Table S7. Differentially expressed proteins from complete cardiomyocyte secretome data identified using a filtering approach. Log2FC = log2 fold-change of Fabry samples compared to controls (mean of 7 biological replicates for each line); AvExp = average expression across all samples as log2 value; t = t statistic; adj.P.Val = p value with Benjamini-Hochberg adjustment.

Extended Experimental Procedures

hPSC culture and cardiac differentiation

The derivation of iPSC lines from two male Fabry disease patients carrying non-sense mutations in *GLA* was previously described (Itier et al., 2014). The patient fibroblasts were obtained from the Coriell Institute for Medical Research. In this article, clone GM00107 (c.485G>A; W162X) is referred to as Fabry 1 and clone GM00881 (c.658C>T; R220X) as Fabry 2. Four healthy male control iPSC lines were also derived internally from skin fibroblasts using the same protocol. MAN13 hESCs were kindly provided by the Kimber lab (Ye et al., 2017). MAN13 hESCs are a UK Stem Cell Bank registered line and this study was performed in full compliance with the laws, regulations and procedures that govern hESC use in the UK. The study conducted is in full compliance with Sanofi guidelines on the use of hESCs. hPSC lines were maintained in mTeSR medium (Stem Cell Technologies), on Matrigel (Corning) and passaged weekly using ReLeSR (Stem Cell Technologies). For cardiac differentiation cultures were expanded for 6 days to near confluence and then treated as follows: 10 ng/ml BMP4 + 4 µM CHIR991021 (Miltenyi Biotec) for 48 hours and then 5 µM Wnt C-59 (Selleckchem) for 48 hours, in RPMI 1640 medium containing 213 µg/ml L-ascorbic acid 2-phosphate, 500 µg/ml recombinant human albumin (A9731 Sigma-Aldrich), 100 pg/ml bFGF (Miltenyi Biotec) + 1:100 000 ITS-X (Thermo Fisher), or in RPMI 1640 + insulin-free B27. Cells were then maintained in RPMI + bFGF and ITS-X until day 11 of differentiation when they were dissociated with TrypLE Select (Thermo Fisher) and transferred to suspension in ultra-low attachment plates (Corning, #3471) for 3 days. For suspension culture and plating, cells were maintained in cardiomyocyte medium (CM): DMEM/F12 + 0.25% Bovine Serum Albumin + 1 mM Creatine, 5 mM Taurine, 0.5 mM L-carnitine, 50 µg/ml α-MTG, 50 µg/ml AA-2P, 1x Trace Elements A, B and C (Corning), 1x Chemically Defined Lipids (Thermo Fisher), 1x Glutamax, 0.5x pen/strep and 1 µM Thiazovivin (Stemgent), plus the addition of 1% FCS. On day 14 of differentiation the aggregated cardiomyocytes were purified using a MACS column based PSC-Derived Cardiomyocyte Isolation Kit (Miltenyi, #130-110-188) and replated on Matrigel-coated plastic. After two days the medium was changed to a maturation promoting formula: CM (as above) + 5 nM T3 + 5 nM dexamethasone + 20 µM palmitic acid (conjugated to fatty-acid free BSA) + 0.25% FBS, as supported by our previous work (Birket et al., 2015). Unless otherwise stated, reagents were from Sigma-Aldrich. Cardiomyocytes were analysed between day 35 and day 60 of differentiation and later dissociations were performed with TrypLE Select (Thermo Fisher).

CRISPR-Cas9 gene editing

We initially attempted to directly convert the *GLA* c.658C>T using a sgRNA targeting this site and supplying a ssDNA donor harbouring the correction flanked by 60nt homology. However, despite high incidence of NHEJ no sequence correction was detected (measured by TIDE/TIDER analysis (Brinkman et al., 2014); data not shown). We thus adopted a selection based CRISPR strategy. First, ribonucleoprotein (RNP) complexes of

CRISPR-Cas9 targeting sequences flanking exon 5, along with a targeting vector, were delivered into Fabry2 iPSCs using an Amaxa 2b nucleofector (program B-16). The crRNA sequences were as follows: 5'-ACAGATTGAACGTCTCCATA-3' and 5'-TATGTGAGAGTACTTAGAGC-3'. RNP complexes were made by complexing 120 pmol cr:tracRNA duplex (IDT) with 67 pmol S.p. Cas9 Nuclease V3 (IDT). 120 pmol carrier DNA was included as an electroporation enhancer (IDT cat # 1075915).

The targeting vector incorporated a 5' homology arm of 801 bp and a 3' homology arm of 750 bp flanking corrected exon 5 (pUC57-5'H-loxP-SA-2A-Puro-loxP-correctExon-3'H). 48h after electroporation, iPSCs were selected with 1 µg/ml puromycin for 48h, then electroporated with 1 µg of Cre expression vector (pCAG-Cre-IRES2-GFP; Addgene plasmid #26646), FACS sorted for GFP expression and directly cloned. Expanded clones were screened by PCR, taking advantage of a unique intronic primer site introduced 3' of exon 5, and sequenced. All analyzed clones were correctly targeted.

To generate *GLA* knockout hPSC lines, MAN13 hESCs and con 3 iPSCs were electroporated as above using a crRNA sequence targeting exon 1 (5'-TTGGCAAGGACGCCTACCAT-3'), which was found to induce in-del mutations with 60-80% efficiency. Cells were cloned by serial dilution and screened by sequencing. Clones with frameshift mutations and undetectable α-gal A expression by Western blot were used for subsequent experiments: MAN13 hESCs (*GLA* c.121_125delTACCA) and con3_iPSCs (*GLA* c. 123delC).

Lentiviral transduction

SCARB2 was overexpressed by lentivirus produced using a pLenti CMV/TO Puro DEST vector (Addgene plasmid #17452). The empty vector, or vector with a GFP insert, were used as controls. Lentiviral particles were concentrated using ultracentrifugation and used to transduce cardiomyocytes at the time of their purification on day 14. Transduced cells were selected with 0.75 µg/ml puromycin and then cultured as normal. Conditioned medium was collected 5-8 days after transduction of cardiomyocytes or 293FT cells.

Gb3 quantification

Measurements were performed using mass spectrometry as previously described (Itier et al., 2014).

Flow cytometry

Differentiated cultures were dissociated using TrypLE Select and labelled with a primary biotin-conjuated anti-CD172a (SIRPA) antibody (Miltenyi-biotec; clone REA144) and then an anti-biotin-VioBright FITC secondary antibody (Miltenyi-biotec; #130-104-514). For TMRM measurements, cells were equilibrated with 2.5nM TMRM overnight before dissociation and measurement. Measurements were performed on a Miltenyi VYB.

Immunocytochemistry and cell size determination

Dissociated iPSC-CMs differentiated for ~40 days were seeded at single cell density on glass coverslips micropatterned with 20 µm wide gelatin lines as previously described (Birket et al., 2015). 4 days later cells

were fixed with 4% paraformaldehyde for 20 mins, permeablized with 0.1% Triton-X, blocked with 1% BSA + 0.4% horse serum for 1 hour, and then labelled overnight at 4°C with primary antibody. Antibodies were as follows: anti-α-actinin (Sigma; A7811), anti-MLC-2V (Proteintech; 10906-1-AP) and anti-myomesin-3 (Proteintech; 17692-1-AP). Imaging was performed with a 60x objective. Individual separated cardiomyocytes were identified based on α-actinin labelling and then MLC-2V or myomesin-3 labelling was also imaged and recorded. Cell area was calculated using a thresholding mask based on α-actinin labelling.

Electrophysiology

Dissociated iPSC-CMs differentiated for 30 days were seeded at single cell density on glass coverslips (Corning, #354086) coated with fibronectin (Sigma, #F0895) and used for measurement 3-8 days later. Patch-clamp experiments were performed at 37°C and cells were continuously perfused with an external solution containing (in mM): 140 NaCl, 5 KCl, 1 $CaCl_2$, 1 $MgCl_2$, 10 glucose, 10 HEPES. pH was set to 7.4 with NaOH 1M. The patch pipettes were pulled from thick-walled borosilicate glass capillaries (Harvard Apparatus, Edenbridge, UK) on a DMZ-universal electrode puller (Zeitz-Instruments GmbH). Electrode impedance was 4–6 MOhms when filled with an internal solution containing (in mM): 110 Kaspartate, 20 KCl, 1 $MgCl_2$, 0.1 Na-GTP, 5 Mg-ATP, 5 phosphocreatine, 1 EGTA, 10 HEPES. pH was adjusted to 7.2 with KOH 1 M. Data were all corrected for liquid junction potentiel (15.4 mV). Ionic currents and action potentials were recorded on isolated spontaneously beating CMs in the whole-cell configuration of the patch-clamp technique using digidata 1550/Multiclamp 700B (Molecular Devices, Sunnyvale, CA, USA) for data amplification and acquisition. Data were acquired at 10 KHz and low-pass filtered at 5 kHz using Clampex software (pClamp 10.5, Molecular Devices). The same following protocol was applied to all CMs: upon seal formation and following patch membrane break, a 50 ms test pulse from a holding potential of -80 to 0 mV was applied in voltage–clamp mode to record Na^+-current maximal amplitude. The patch was then switched to current–clamp mode and spontaneous action potentials (APs) were recorded for 1 min at resting potential without injecting any current.

For currents and AP analysis, Clampfit software was used (pClamp 10.5, Molecular Devices). Na^+-current density was obtained by normalizing Na^+-current maximal amplitude to the cell capacitance and expressed in pA/pF. For AP analysis, common electrophysiological characteristics such as action potential duration at 90% of repolarization (APD90), action potential amplitude and maximum diastolic potential (MDP) were calculated.

Calcium imaging

Dissociated iPSC-CMs differentiated for 30 days were seeded at a density of 5 x 10^4 cells/well in black walled clear bottom 96-well plates (Costar, #655946) coated with Matrigel and used for measurement 3-8 days later. The Cal-520 calcium assay was performed as outlined in the product information sheet provided with the Hamamatsu FDSS *µCell* (Hamamatsu Photonics, Hamamatsu, Japan). Briefly, stock solution of Cal-520 dye was

prepared at 5 mM in DMSO. Assay buffer was made from Hank's Balanced Saline Solution (HBSS) buffered with 20 mM HEPES. Powerload™ concentrate was used to enhance the dye solubility. Cells were incubated in 5 µM of Cal-520 dye for 60 minutes to 90 minutes at 37°C, washed twice with warm dye-free assay buffer and then imaged in 80 µL per well of warm assay buffer. Fluorescence measurements were performed into FDSS *µCell* at 37°C. The microplate was illuminated at 470 nm and fluorescence was measured at 540 nm using the high-speed acquisition mode (100 Hz). The caffeine response was assessed by injecting 10 µl of caffeine at 50 mM using the 96-well dispenser head, resulting in a final concentration of 10 mM. Data acquisition was performed using FDSS*v3* software and analyzed using the WaveForm Analysis, software (Hamamatsu Photonics, Hamamatsu, Japan). Fluorescence measurements are represented as relative fluorescence unit (RFU). For each experiment, spontaneous calcium transients were analyzed over 2 minutes. Frequency, basal fluorescence (F), amplitude (ΔF/F), peak width at 90% of recovery (PWD-90%), corrected peak width (corrected PWD-90%, calculated by Fridericia's formula and rising slope (30%-70%) were measured (exemplified in **Fig. S1**).

Proteome sample preparation

Dissociated iPSC-CMs differentiated for 35 days were pelleted and lysed in a buffer of 1% SDS, 0.25 u/mL benzonase and protease inhibitors for 30 min at 37°C. Protein concentration was measured with BCA (Thermo BCA Protein Assay Kit). Extracted protein was then loaded on a 4-12% SDS-page gel (approx. 12 µg protein per lane) for approx. 3 min. Reduction/alkylation/digestion steps were then performed in gel. Gel slices were subjected to two washing steps with a mixture of 50 mM ammonium carbonate buffer/ACN (50/50), then to reduction by 30 min incubation at 56°C with 20 mM DTT, followed by two more washing steps. Proteins were subjected to alkylation for 30 min in presence of 40 mM iodoacetamide, followed by two washing steps. After the addition of 0.01 µg/µL trypsin, gels slices were incubated overnight at 37°C. The peptide mixture solution was then acidified by addition of formic acid and filtered using a 0.2 µm membrane before injection.

Secretome sample preparation

Cardiomyocytes were washed 3 times and then incubated in FCS- and BSA-free medium for conditioning and collection. The medium composition was: phenol red-free DMEM/F12 + 1:100 000 ITS-X + 1 mM Creatine, 5 mM Taurine, 0.5 mM L-carnitine, 50 µg/ml α-MTG, 50 µg/ml AA-2P, 1x Trace Elements A-B-C, 1x Chemically Defined Lipids, 1x Glutamax, 0.5x pen/strep. Conditioned medium was collected daily for up to 5 days, centrifuged at 1000g for 10 min to remove debris and frozen at -80°C.

Enrichment of soluble proteins was performed using NanoZeolites obtained from NanoScape AG; Germany. Adsorption of proteins on the surface was carried out by incubating the conditioned medium with NanoZeolite beads for 90 min at 4°C. After centrifugation at 16000g for 20 min, 4°C, pellets were washed twice with 100 mM ammonium carbonate buffer. Proteins bound to the NanoZeolite beads were reduced with 10 mM DTT,

0.05% AALS in 50 mM ammonium carbonate buffer at 56°C for 30 min and subsequently alkylated by addition of 20 mM iodoacetamid and 30 min incubation step at room temperature. Over-alkylation was limited by adding 20 mM DTT. Trypsin was then added at 0.01 µg/µL, followed by an overnight incubation at 37°C. After digestion AALS was precipitated in presence of 1% TFA and filtration performed at 0.2 µm.

For Western blotting, protein from 3ml of conditioned medium was precipitated using ice cold acetone (1:4), incubated for 1 h, 4°C, pelleted at 3500G for 30 min, 4°C, resuspended in loading buffer, heated at 95°C for 5 min and centrifuged again to remove debris.

Liquid chromatography-mass spectrometry

Peptides were separated on a nanoacquity nanoLC (Waters) coupled to an Orbitrap Fusion mass spectrometer (Thermo) via a nanoelectrospray source. Peptides were loaded on a trap column (Symetry C18 100A 180 µm x 2 cm 5 µm, Waters) and separated on a BEH C18 column (Peptide BEH-C18 130A, 1.7 µm, 75 µm x 250 mm column temperature: 40°C, Waters) by a 140 min gradient from 5% to 85% ACN (+0.1% formic acid) at a flow rate 0.3 µl/min. The Fusion was operated in a data dependent mode with a survey scan range of 325-1200 m/z and a resolution of 120 000 at m/z 200. Selection of precursors was performed using the top speed mode and subjected to high energy collision in the HCD cell at a normalized collision energy of 23. Fragmentation spectra were acquired in the trap in the rapid mode scan rate. Dynamic exclusion of sequenced peptides was set to 50 s to reduce repeated peptide sequencing. Threshold for ion injection time and ion target were set to 50 ms and 2E5 for the survey scans and 35 ms and 1E4 for the MS/MS scans respectively.

Label free quantification

MaxQuant software (version 1.5.3.8) was used to analyse MS raw files. MS/MS spectra were searched against the human Uniprot FASTA database (version February 2017). Cysteine carbamidomethylation was applied as fixed and N-terminal acetylation, pyroQ and methionine oxidations as variable modifications. Enzyme specificity was set to Trypsin/P with a maximum of 3 missed cleavages. A false discovery rate (FDR) of 1% was applied at the peptide and protein level. Normalization of samples intensities was performed via Maxquant label free quantification algorithm (MaxLFQ).

Proteomics data analysis

Data analysis and visualization was performed using the Perseus software (version 1.5.5.3) and then using R/Bioconductor tools. Proteins identified with ≥1 unique peptide and positive gene identification were used for quantification. Logarithmic transformation was applied, and the median of injections was calculated. Missing values were imputed with the mean of the minima of all data. Protein identifications were required to have more than 50% valid values in at least one group of biological replicates.

Heatmaps and hierarchical clustering plots were generated using the ‘pheatmap’ package. Limma analysis was performed contrasting control and Fabry protein sets with >1 unique peptide with the following arguments for the topTable() function: adjust=”BH”, lfc = 0.585 and p.value = 0.1 (Ritchie et al., 2015). Outputs were also visualised using the volcanoplot() function. A filtering approach to identify differentially abundant proteins was also applied by first calculating the difference in expression of each Fabry sample compared to the mean of all the control samples in that group. Proteins with ≥0.5 log2 fold-change for both Fabry lines individually and an adjusted p-value <0.05 using all samples (one-sample t-test; μ = 0) were selected. Each sample was considered as an independent replicate. The lower p-value used for filtering was used to prioritize the candidates to a sensible number. Proteins with more than one missing value in the higher expressed group were discounted, after inspection. Classification as plasma protein and presence of signal peptide was performed using the Plasma Proteome Database (http://www.plasmaproteomedatabase.org/) and SignalP (http://www.cbs.dtu.dk/services/SignalP/) via the Human Protein Atlas (https://www.proteinatlas.org/). Protein-protein interaction network analysis was performed using STRING (https://string-db.org).

Western blot

Dissociated cell pellets were lysed in RIPA buffer + protease inhibitors (Thermo Scientific; #78440) + 80U/ml DNase I (Sigma, #D4527) + 5mM EDTA, sonicated briefly and then centrifuged to remove cell debris. 10 or 30 μg of cell lysate was heated at 95°C in loading buffer, loaded onto a 4-20% SDS-polyacrylamide gel (Biorad; #5678094) and transferred to a PVDF membrane using a Trans-Blot Turbo Transfer Pack (Biorad; #170-4157). Membranes were blocked in 5% milk in TBS-T and incubated in primary antibody overnight at 4°C. Antibodies and their dilutions were as follows: Anti-LIMP-2 (Abcam ab176317; 1:1000), anti-HSP70-2 (Abcam ab108416; 1:1000), anti-calsequestrin 2 (Proteintech 18422-1-AP; 1:1000), anti-GCase (internal; 1:500), anti-LAMP-2 (Santa-Cruz Sc-18822; 1:500), anti-GLA (Abcam ab168341; 1:1000), anti-CTSF (Abcam ab200650; 1:500), anti-collagen I (SouthernBiotech 1310-01; 1:1000), anti-collagen IV (Novocastra NCL-COLL-IV; 1:1000), anti-GAPDH (Abcam; 1:3000). HRP-conjugated secondary antibodies were as follows: anti-mouse IgG(H+L) HRP (Thermo Fisher #32230; 1:10 000) and anti-rabbit IgG(H+L) HRP (Thermo Fisher #32460; 1:10 000).

References

Birket, M.J., Ribeiro, M.C., Kosmidis, G., Ward, D., Leitoguinho, A.R., van de Pol, V., Dambrot, C., Devalla, H.D., Davis, R.P., Mastroberardino, P.G., et al. (2015). Contractile Defect Caused by Mutation in MYBPC3 Revealed under Conditions Optimized for Human PSC-Cardiomyocyte Function. Cell Rep. *13*, 733.

Brinkman, E.K., Chen, T., Amendola, M., and van Steensel, B. (2014). Easy quantitative assessment of genome editing by sequence trace decomposition. Nucleic Acids Res. *42*, e168–e168.

Itier, J.-M., Ret, G., Viale, S., Sweet, L., Bangari, D., Caron, A., Le-Gall, F., Bénichou, B., Leonard, J., Deleuze, J.-F., et al. (2014). Effective clearance of GL-3 in a human iPSC-derived cardiomyocyte model of Fabry disease. J. Inherit. Metab. Dis. *37*, 1013–1022.

Mi, H., Huang, X., Muruganujan, A., Tang, H., Mills, C., Kang, D., and Thomas, P.D. (2017). PANTHER version 11: expanded annotation data from Gene Ontology and Reactome pathways, and data analysis tool enhancements. Nucleic Acids Res. *45*, D183–D189.

Ritchie, M.E., Phipson, B., Wu, D., Hu, Y., Law, C.W., Shi, W., and Smyth, G.K. (2015). limma powers differential expression analyses for RNA-sequencing and microarray studies. Nucleic Acids Res. *43*, e47.

Ye, J., Bates, N., Soteriou, D., Grady, L., Edmond, C., Ross, A., Kerby, A., Lewis, P.A., Adeniyi, T., Wright, R., et al. (2017). High quality clinical grade human embryonic stem cell lines derived from fresh discarded embryos. Stem Cell Res. Ther. *8*, 128.
